# Clinical Implications of Human Population Differences in Genome-Wide Rates of Functional Genotypes

**DOI:** 10.3389/fgene.2012.00211

**Published:** 2012-11-01

**Authors:** Ali Torkamani, Phillip Pham, Ondrej Libiger, Vikas Bansal, Guangfa Zhang, Ashley A. Scott-Van Zeeland, Ryan Tewhey, Eric J. Topol, Nicholas J. Schork

**Affiliations:** ^1^The Scripps Translational ScienceLa Jolla, CA, USA; ^2^Scripps HealthLa Jolla, CA, USA; ^3^Department of Molecular and Experimental Medicine, The Scripps Research InstituteLa Jolla, CA, USA

**Keywords:** clinical sequencing, congenital disease, whole genome sequencing, population genetics

## Abstract

There have been a number of recent successes in the use of whole genome sequencing and sophisticated bioinformatics techniques to identify pathogenic DNA sequence variants responsible for individual idiopathic congenital conditions. However, the success of this identification process is heavily influenced by the ancestry or genetic background of a patient with an idiopathic condition. This is so because potential pathogenic variants in a patient’s genome must be contrasted with variants in a reference set of genomes made up of other individuals’ genomes of the same ancestry as the patient. We explored the effect of ignoring the ancestries of both an individual patient and the individuals used to construct reference genomes. We pursued this exploration in two major steps. We first considered variation in the per-genome number and rates of likely functional derived (i.e., non-ancestral, based on the chimp genome) single nucleotide variants and small indels in 52 individual whole human genomes sampled from 10 different global populations. We took advantage of a suite of computational and bioinformatics techniques to predict the functional effect of over 24 million genomic variants, both coding and non-coding, across these genomes. We found that the typical human genome harbors ∼5.5–6.1 million total derived variants, of which ∼12,000 are likely to have a functional effect (∼5000 coding and ∼7000 non-coding). We also found that the rates of functional genotypes per the total number of genotypes in individual whole genomes differ dramatically between human populations. We then created tables showing how the use of comparator or reference genome panels comprised of genomes from individuals that do not have the same ancestral background as a patient can negatively impact pathogenic variant identification. Our results have important implications for clinical sequencing initiatives.

## Introduction

Whole genome sequencing (WGS) has enabled the search for inherited DNA sequence variants that are responsible for idiopathic diseases affecting a number of individuals (Biesecker et al., 2009; Lupski et al., 2010; Roach et al., 2010; Bainbridge et al., 2011; Worthey et al., 2011; Lyon and Wang, 2012). The strategy for identifying such variants is intuitive, as it involves two reasonable assumptions: first, that the responsible variants are unique to the individuals affected by the diseases and second, that these variants are likely to exhibit molecular effects pronounced enough to be captured by available bioinformatic analyses of those variants (Rope et al., 2011; Yandell et al., 2011). However, this strategy is not necessarily trivial to implement. For example, determining that a variant in the genome of a patient with an idiopathic condition is unique to that patient requires contrasting variants in that patient’s genome with variants observed on the genomes of a “reference” set of individuals. The reliability of this comparison directly impacts the ease and ultimate success of a search for a pathogenic variant, and differences in the ancestry of the individuals whose genomes are used in a reference set and the patient’s ancestry could influence reliability in pronounced ways. In addition, the reliability of the bioinformatics tools and other sources of information used to make claims about the functional impact of a variant are also crucial. Of course, many diseases, idiopathic or not, have a complex molecular basis even though they exhibit pronounced clinical phenotypic expressions and severe health consequences that on the surface appear to be due to a singular, rare or overtly monogenic genomic perturbation and thus may require a different strategy for identifying their genetic determinants (Biesecker et al., 2009; Gonzaga-Jauregui et al., 2012).

We considered the impact that the use of a reference set of genomes obtained from individuals who do not have the same ancestry as a patient would have on claims that a variant identified in a patient with an idiopathic condition is unique to that patient. We also considered the influence of the use of different bioinformatic tools on the identification of pathogenic variants when the ancestries of individuals used to construct a reference genome panel vary. We did this by exploring both allele frequency differences and per-genome number and rate of bioinformatically predicted functional variants – both coding and non-coding – across multiple global populations from a WGS perspective. The motivations for pursuing our study were straightforward: although many studies have explored the differences in the frequencies of specific genetic variants between populations in a way that has shed light on the contribution of those genetic differences to actual phenotype frequency differences between those same populations (Stephens et al., 1998; Evans et al., 2005), they have not necessarily addressed issues concerning the identification of pathogenic variations responsible for idiopathic conditions.

In this light, it is important to consider just how previous studies have fallen short of providing comprehensive insight into why searches for pathogenic variants must be sensitive to both patient and genome reference panel member ancestries and bioinformatics strategies for characterizing the functional effects of variants. A number of studies have explored the distant genealogical relationships between individuals in populations that exhibit phenotypic differences by either contrasting variant frequencies between those populations (Rosenberg et al., 2002; Conrad et al., 2006; Li et al., 2008), by assessing individual DNA sequence similarities among individuals within and between those populations (Nievergelt et al., 2007), or by investigating evidence for selection acting on variants based on population variant frequency differences,(Nielsen et al., 2007; Pickrell et al., 2009) but have not necessarily focused on the overt differences in the functional content of individual genomes, which is of great relevance to searches for pathogenic variants influencing idiopathic conditions.

Recent studies by Bustamante and colleagues are an exception, as they have considered differences in the frequencies of variants in coding elements with likely functional effects in ∼10,000 genes between European and African populations (Boyko et al., 2008; Lohmueller et al., 2008). Specifically, they found evidence for a greater proportion of homozygous, likely phenotypically impactful, non-synonymous coding Single Nucleotide Variants (ns cSNVs) in a sample of European American individuals compared to a sample of African American individuals. They further showed through simulation studies that the higher proportion of likely functional ns cSNVs in the European population was consistent with the effects of a historical bottleneck resulting from out-of-Africa migrations. The results of these studies, and further larger-scale surveys by the same group (Gravel et al., 2011), could also provide a partial explanation for overall phenotypic differences between African and European populations.

As important as the studies by Bustamante and colleagues and related studies are (Pelak et al., 2010; Casto and Feldman, 2011; Moore et al., 2011; Nelson et al., 2012; Tennessen et al., 2012), they have important limitations. First, many focus on specific variant types (e.g., SNVs) and, at least in the case of the focused surveys by Bustamante and colleagues, coding variants only (Boyko et al., 2008; Lohmueller et al., 2008; Gravel et al., 2011). Second, most studies do not consider the use of a broad array of functional prediction tools for the variants, but confine attention to a few tools. Third, many studies do not necessarily consider a whole genome perspective but rather focus on particular genomic regions or loci (e.g., exons or candidate genes). Fourth, of the WGS studies interrogating functional variant frequency and rate differences between individuals or groups of individuals that have been pursued to date, they do not exploit a common sequencing technology (Moore et al., 2011), focus on a single disease or do not necessarily consider the specific influence of ancestry on sources of genetic variation (Pelak et al., 2010). Fifth, they do not emphasize rates of variation on a per-individual whole genome basis but rather focus on contrasts involving population-level summary information regarding frequencies and rates of variants. Sixth, they focus on contrasts involving only a few populations and, in the context of an initial study (Lohmueller et al., 2008), consider samples of European and African American individuals who may harbor some degree of admixture.

We sought to overcome limitations of previous studies by exploring the differences in the genome-wide rates of non-ancestral or “derived” variants (i.e., those variants in the human lineage that deviate from the chimpanzee genome) that have a predicted or likely functional effect between 52 individuals from 10 different global populations. Pronounced differences in the functional content of genomes between individuals with different ancestries would suggest that reference panels for pathogenic searches must be made up of some individuals with same ancestry as the patient, or the claim that a variant is unique to a patient may be invalid. To assess this, we leveraged data generated by a single sequencing technology generated at an appropriate sequencing depth (∼60×). Our ultimate goal was to characterize differences in the standing variation in contemporary populations that may be of phenotypic relevance and ultimately impact searches for pathogenic variants in WGS analyses of individuals with idiopathic conditions. More precisely, based on our data and functional variant analyses, we assessed the impact of ignoring the origins of genomes used in reference panels created to facilitate the identification the causative variants for rare and idiopathic conditions. We did this in part by simulating genomes containing known pathogenic variants and then determining the likelihood of identifying that variant when the genomes of individuals with different genetic backgrounds are contrasted to a genome harboring the known pathogenic variant. As a byproduct of our investigation we have created one of the largest collections to date of putative human functional DNA sequence variants. In addition, our analyses further elucidate the breadth of functional variation in contemporary global human gene pool. We emphasize that most of our analyses considered both single nucleotide variants (SNVs) as well as small insertion and deletion variants (indels) in both coding and non-coding regions of the genome across individual genomes, although for some of our analyses we focused on coding variants only due to the lack of available contrasting data sets to our own.

Because there are many steps in the analyses we pursued to allow us to make conclusions about the need for sensitivity to genetic background and ancestry, as well as bioinformatic tool use, in the construction of reference panels exploited in searches for pathogenic variants influencing idiopathic conditions, we give a brief synopsis of each of these steps. The material presented in the Sections “Materials and Methods” and “Results” is ordered to follow these steps. We first obtained WGS data from individuals thought to have diverse ancestral backgrounds and verified this diversity using standard techniques. We then determined how the individual genomes differed from one another, essentially identifying variable or “variant” genomic positions, by contrasting each genome to the available human genome reference sequence, which is flawed for our purposes for the reasons to be provided, as well as the chimp genome, which is more appropriate for our purposes. We then characterized the likely functional impact of each variant position using what we believe is the most comprehensive set of bioinformatics tools assembled and applied in one setting to date. We then contrasted the predicted functional content across the genomes of individuals with different ancestries in terms of the absolute number of functional variants on each genome, the rate of functional variants per total number of individual genome variants, and the number of population-specific variants on each genome and within each population. Finally, we simulated settings in which we knew there was pathogenic variant on an individual simulated patient’s genome and then performed a bioinformatically guided search among all the variants on that genome using reference panels made up of genomes from individuals whose ancestries both matched and did not match the ancestry of the simulated patient.

## Materials and Methods

### Whole genome sequence data from the complete genomics, Inc., public domain repository

We obtained publicly available complete genome sequence data on 69 individuals of high quality (∼60× coverage, ∼97% bases called) produced by Complete Genomics, Inc. (CGI) by downloading these data from the company’s website^1^. The assembly of the genomes as well as variant calling for them has been described in the literature (Drmanac et al., 2010; Roach et al., 2010). We ultimately used the genotypes from the available “MasterVar Beta” files provided by CGI directly and did not consider additional filtering steps for the analysis of genotypes beyond those that went into the construction of the public domain files. The 69 individual genomes consisted of 22 individuals of Northern European ancestry (abbreviated here as CE for the CEPH or CEU HapMap Population (Consortium, 2005; Frazer et al., 2007), 10 individuals of Yoruban ancestry (YR), five individuals each of Mexican (ME), and African ancestry living in Dallas (AS), four individuals each of Japanese (JP), Han Chinese (CH), Italian (TS), East Indian (GI), Maasai Kenyan (MK), and Luhya Kenyan (LW) ancestry, and three individuals of Puerto Rican ancestry (PU). Thirteen CE individuals were the offspring of a couple of other CE individuals and were excluded from the analysis. One YR individual was the offspring of a YR couple and was excluded. The Puerto Rican individuals were a mother-father-offspring trio and were also excluded. We therefore ultimately considered 52 individuals from 10 different global populations in our analysis (the data sets used that included annotated variants are available from the authors). To show how some of our results apply to other data sources, we also leveraged sequence data available from the 1000 genomes project^2^. For one set of analyses, we considered an ancestry assessment-verified (see below) female European individual’s genome that was sequenced by CGI independently of the 69 genomes we obtained from the public domain.

### Ancestry assessment

**Figure A1:**
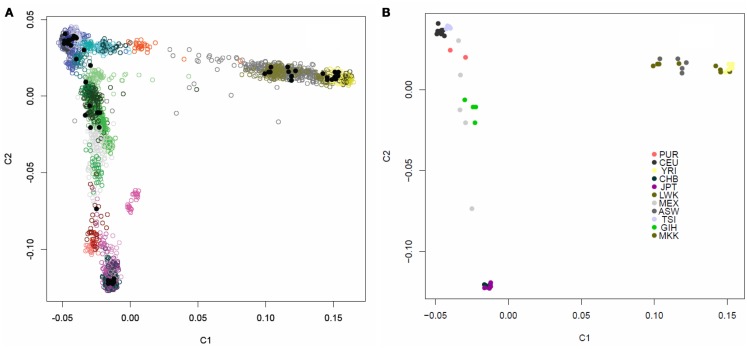
**Multidimensional scaling plot of the similarity of the 54 unrelated individuals with complete genome data**. **(A)** The 54 individuals (black dots) overlaid on 4,123 individuals of known ancestry based on 16,411 ancestry informative markers. The individuals with known ancestries were obtained from public repositories and are color coded by continent with shading indicating subpopulations within those continents (Blue, Europeans; Yellow, Yorubans; Purple, East Asians; Red: Native Americans; Green: Central Asians; Grey: African Americans). **(B)** Multidimensional scaling plot of the similarity of the content of 54 individuals with complete individual genomes without the overlay of other individuals. Color coding for these 54 individuals based on their known ancestries is given in the inset.

**Figure A2:**
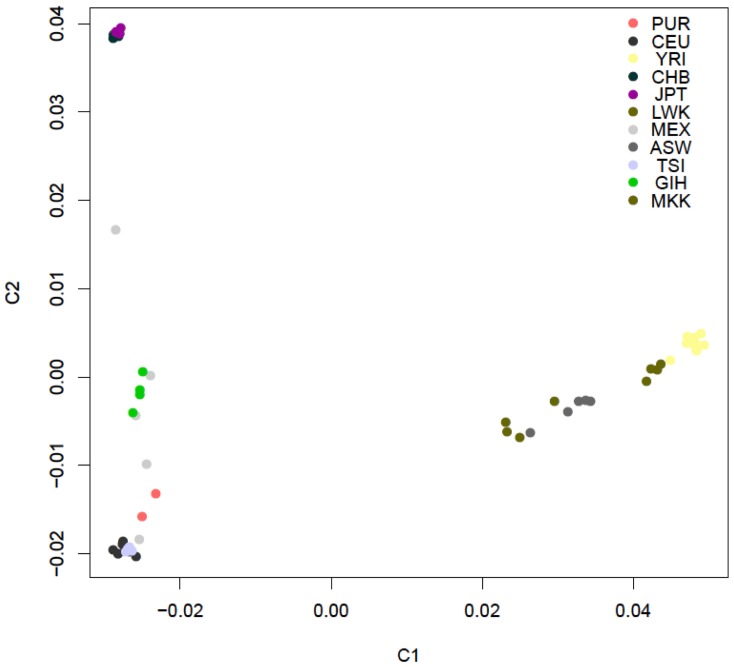
**PCA plot of the similarity of the 54 unrelated individuals with complete genome data based on 19,208,882 SNVs obtained from the complete sequencing data without regard to a reference panel of individuals with global ancestries (shading according to population is provided by the inset)**.

We assessed the genetic background similarity of the 69 individual genomes downloaded from the CGI website, in addition to the single independently sequenced European female’s genome, by constructing identity-by-state (IBS) allele sharing similarity matrices using 16,411 markers which had also been genotyped on 4,123 individuals in various public domain databases for whom ancestry was known. We also calculated IBS allele sharing matrices based on 19,208,882 variants determined from the WGS for the 52 individuals ultimately used on our analyses in addition to the parents in the Puerto Rican trio. We then applied multidimensional scaling (MDS) analysis to the sharing matrices to determine patterns in genetic background similarity of the individuals (Reich et al., 2008). Figure A1 in Appendix depicts the first two PCs for the allele sharing determined through the use of the 16,411 markers genotyped on the 4,123 reference individuals as well. Figure A2 in Appendix depicts the first two PCs the allele sharing matrix determined through the use of the 19,208,882 markers identified in the sequencing of the genomes of the 52 + 2 individuals. It is quite clear from these analyses and plots that the 52 individuals whose genomes we are studying have diverse ancestries that are consistent with the populations they are reported to represent. Additional analysis of the single European female’s genome sequenced independently of the 52 genomes verified her European ancestry (data not shown).

### Variant allele determination

To catalog all position-specific differences (i.e., variants) between the 52 genomes we considered two different strategies. We first compared each genome to the human genome reference (version hg18) and then determined the ancestral allele of each variant by comparing the genomes to the available chimp genome reference. We briefly describe each of these efforts below.

#### Human reference allele determination

We determined the sequence position of each variant site relative build hg18 of the human genome provided on the UCSC browser (Fujita et al., 2011). We did this for variant types we could determine from the CGI variant files in the public domain, including SNVs, small insertion and deletion variants and multinucleotide variants (i.e., small stretches of sequence where all the adjacent nucleotides present differ from the reference genome). We could thus determine the number and type of “non-reference” variants each of the 52 individual genomes we studied possessed. We did not consider large structural variations nor did we consider large copy number variants (CNVs) and other large repetitive element-based variants. The use of the human genome reference for assessing inter-population differences in the frequency and rate of functional variant is problematic since the available UCSC Genome Browser human genome reference (hg18) is constructed from DNA of European individuals. Thus, the frequency or “labeling” of nucleotides as variants that are “reference” or “non-reference” in other populations would be dictated by what is present on the genomes of individuals of European ancestry, if the human genome reference (hg18) is used. This can easily lead to interpretive biases regarding the relationships between populations and genomic differences between those populations (Hernandez et al., 2007; Boyko et al., 2008; Lohmueller et al., 2008). In addition, as considered in the Section “Discussion,” functional element determination based on single individual genomes or genomes from individuals with a unique ancestry is problematic due to structural differences in genomes that may impact the very definition of a functional element (Balasubramanian et al., 2011). Thus, we characterized variants as “non-reference” merely for the sake of consistency with the literature and to allow us to determine a reasonable and accepted approximation of the functional impact of the variants we observed in the 52 genomes.

#### Ancestral allele determination

We also determined the ancestral allele of each variant site using the PanTro2 build of the chimpanzee genome (Lohmueller et al., 2008). In essence, we determined which allele at a variant site among the 52 genomes we studied was present on the chimpanzee genome (i.e., the “ancestral” allele) and which was not (i.e., the “derived” allele). We determined ancestral alleles using alignment information between the PanTro2 build of the chimpanzee genome with the human genome (hg18) from the UCSC Genome Browser (Chiaromonte et al., 2002; Kent et al., 2003; Schwartz et al., 2003). When ancestral alleles could not be determined, we switched to alignments between the RheMac2 build of the Macaque genome with the human genome (hg18) and ignored positions when both alignments failed to reveal ancestral information. Ultimately, we pooled all non-reference variants (determined from the comparison to the human genome reference hg18 as described above) seen across individuals and determined whether these variants matched ancestral alleles. In such cases, these non-reference variants revealed that the deviation is actually in the human reference genome (hg18) and not the non-reference variant. Subsequently, all individuals that harbored the non-reference variant no longer carried the variant while all other individuals with the reference allele now contained a “derived” or non-ancestral variant.

Given information about which variants were reference/non-reference and ultimately ancestral or derived, we assigned, for each individual genome at each variant site the labels “reference” or “non-reference,” “ancestral” or “derived.” We then assigned additional genotype labels to each genome as, e.g., “homozygous derived,” “heterozygous,” or “homozygous ancestral” for all variant site positions for which we had ancestral allele information. With this information, we could determine derived variants (likely functional or not, see below for our assessment of the functional impact of variants) that were only observed on a single genome (genome-specific or “novel” variants within the context of our dataset), derived variants that were only seen among the genomes of individuals within a specific population (“population-specific” alleles or variants), as well as the overall and population-specific frequencies of the variants.

### Variant functional element mapping

As noted, we mapped all variants to the UCSC Genome Browser human reference genome, version hg18. Subsequently, we took all variant positions and determined their proximity to known genes and functional genomic elements using the available databases from the UCSC Genome Browser (Fujita et al., 2011). All transcripts of the nearest gene(s) were associated with a variant, and functional impact predictions (see below) were made independently for each transcript. If the variant fell within a known gene, its position within gene elements (e.g., exons, introns, untranslated regions, etc.) was recorded for functional impact predictions depending on the impacted gene element. All variants falling within an exon were analyzed for their impact on the amino acid sequence (e.g., synonymous, non-synonymous, non-sense, frameshift, in-frame, intercodon, etc.).

### Variant functional effect predictions and annotations

Once the genomic and functional element locations of each variant site were obtained, we leveraged a suite of bioinformatics techniques and programs to “score” the derived alleles (i.e., derived variant nucleotides) for their likely functional effect on the genomic element they resided in (Plumpton and Barnes, 2007). Derived variants were assessed for potential functional effects for the following categories: non-sense SNVs, frameshift structural variants, splicing change variants, probably damaging (PD) non-synonymous coding (nsc) SNVs, possibly damaging nscSNVs, protein motif damaging variants, transcription factor binding site (TFBS) disrupting variants, miRNA-BS disrupting variants, exonic splicing enhancer (ESE)-BS disrupting variants, and exonic splicing silencer (ESS)-BS disrupting variants. Details of the strategies and algorithms used, as well as the criteria for labeling a variant as “functional,” are provided in the Supplementary Material, but suffice it to say that the functional prediction algorithms we used exploit a wide variety of methodologies and resources to predict variant functional effects, including conservation of nucleotides, known biophysical properties of DNA sequence, DNA sequence determined protein and molecular structure, and DNA sequence motif or context pattern matching.

### Between and within population functional variant frequency and rate data analyses

We compared the frequencies and rates of functional and non-functional derived variants among the genomes of individuals with different ancestries in a few different settings. The methodologies associated with each of these settings are described briefly in isolation below.

#### General population comparisons

To compare frequencies and rates of different types of variants (reference or derived; predicted functional or predicted non-functional; coding, TFBS, etc.) across the 10 populations, graphical displays and linear regression techniques were used. For the regression analyses, simple dummy variables for each of the 10 ancestral populations were created (i.e., a value of 1.0 was assigned to an individual genome that belonged to aspecific ancestral population and 0.0 otherwise) and were used as independent variables in a regression analysis with either the absolute number of variants of a specific type on a genome, or the rate of that variant type per all of an individual’s genomic variants, as a dependent variable. For these comparisons, the YR (Yoruban) population was taken as a reference, such that the estimated regression coefficients reflect deviations from the YR population. We used Tukey’s “Honestly Significantly Different (HSD)” method for evaluating pairwise differences between individual populations for the different variant types from an analysis-of-variance (ANOVA). The HSD method allowed us to make appropriate statistical inferences given the number of pairwise population comparisons we made (Braun, 1994).

#### Homozygous variant comparisons

We also compared the frequency and rate of variants of the different types that were homozygous across the populations using regression methods analogous to those described above. We also considered graphical displays of the frequency and rate differences of homozygous variants across the populations.

#### Population-specific variant comparisons

We determined all the variants that were only found on genomes of individuals with ancestries associated with three major continental populations. We first combined the genomes from CE and TS subpopulations to form a European (EUR; *n* = 13) population, the JP and CH subpopulations to form an Asian (ASN; *n* = 8) population, and the YR, MK, and LW subpopulations to form an African (AFR; *n* = 17) population. We excluded the AS subpopulation from the formation of the African (AFR) population because that population represents African American individuals sampled from within the United States and therefore could reflect admixed individuals. We then determined the number of variants that were observed only within each population for each variant category, and both aggregated the total number and rate of such variants in each population also assessed the rate of such variants in each individual genome in each population. *z*-tests assessing the equality of these frequencies were performed. We also used regression analyses to assess differences between the frequency and rates of African, European, and Asian population-specific variants. The African population was used as a reference and dummy variables for European and Asian ancestry were constructed. Pearson’s correlation coefficients were calculated between rates of population-specific functional variants relative to all population-specific variants as well as relative to all variants.

### Simulation studies using known pathogenic variants

We assessed the impact of using inappropriately ancestry-matched reference panels in efforts to identify patient-specific pathogenic variants responsible for an idiopathic condition via simulation studies. These simulation studies leveraged both the data and insights associated with our assessment of global functional variant diversity involving the 52 CGI genomes. We first took 506 known Charcot-Marie-Tooth (CMT) syndrome causing variants from the OMIM database and computed their Polyphen2(Ramensky et al., 2002; Adzhubei et al., 2010) and SIFT scores (Ng and Henikoff, 2003; Kumar et al., 2009; or rather, technically, 1.0-SIFT score, which we will refer to as the “SIFT score”) and obtained their averages (average Polyphen2 score = 0.825, average SIFT score = 0.931, and average of the average value of the Polyphen2/SIFT scores = 0.878) as well as 567 known Cystic Fibrosis (CF) causing variants (average Polyphen2 score = 0.769, average SIFT score = 0.891, and average Polyphen2/SIFT score = 0.830) and “implanted” variants reflecting these scores in a European individual’s whole genome sequence variant list. Polyphen2 and SIFT are bioinformatics programs implementing procedures for determining the likely functional significance of non-synonymous coding SNVs and were including in the suite of programs we used to characterize the likely functional effect of variants (see above and Supplementary Material). This European individual was sequenced by Complete Genomics, Inc., in the same way as the 52 individuals taken from the CGI repository, but was not part of that panel of 52 individuals.

By placing known disease-causing coding variants among the other variants on this individual’s genome, we could see if we could identify them as likely pathogenic and disease-causative among all the other coding variants on that individual’s genome. This activity was pursued by comparing these coding variants on this individual’s genome to reference panel genomes made up of individual genomes from among the 52 CGI genomes we studied with the same and different ancestries. We pursued this using different bioinformatics functional prediction tools to assess their impact on pathogenic variant identification as well. We chose to explore CMT variants and CF variants since CMT variants act in a dominant fashion and CF variants act in a recessive fashion. We also chose to leverage an individual not sequenced along with the 52 CGI public domain genomes since the variants on this individual’s have not been deposited into dbSNP and other databases and thus many of them are not likely to have been studied by other groups.

We also implanted CMT and CF variants with the scores described above in the variant lists of a randomly chosen African (taken from the AS population, which could reflect African American ancestry), Mexican, East Indian, and Puerto Rican genomes from the total of the 69 individuals for which we had WGS data from the CGI repository. We determined the number of ns cSNVs (i.e., coding variants) that would be considered novel (i.e., patient-specific) among these individuals’ sets of variants with predicted functional scores from Polyphen2 (Ramensky et al., 2002; Adzhubei et al., 2010) SIFT,(Ng and Henikoff, 2003; Kumar et al., 2009), and the average Polyphen/SIFT score, greater than those associated with the implanted, known disease-causing CMT and CF mutations when compared to different reference panel genomes sets. These reference panel sets included the 1000 Genomes Project exome sequencing data (as of October 2011; Consortium, 2010; Gravel et al., 2011), both combined across all populations considered in the Project and for each of the European, Asian, and African variant sets individually. We also created reference sets for variants from all 52 individuals for which we had WGS data as well as eight randomly chosen Europeans, Asians, and Africans from these 52. Finally, we considered a combined reference variant set that included the 1000 Genomes data and the WGS data for the 52 individuals. We pursued these analyses by assuming that the CMT mutation was dominant and the CF mutation was recessive (i.e., for the CF mutation we considered as novel only homozygous genotypes not observed in the reference panels whereas for the CMT mutation we considered as novel any genotype that was not observed in the reference panels, homozygous, or heterozygous).

## Results

### Variant identification

From the 52 individual genomes we identified 24,277,549 “non-reference” variants that deviated from build hg18 of the human reference genome represented in the UCSC browser (Mangan et al., 2009; Fujita et al., 2011). This included 19374542 SNVs, 1941800 insertions, 2282925 deletions, and 678282 multinucleotide variants. We defined as “novel” a variant in one genome that was not present on the other 51 genomes. Note that this definition of “novel” is specific to our data set, since any variant we observed may have been observed before in other studies. We did not filter for novel variants using other publicly available databases since the DNA samples from the 52 individuals sequenced by CGI are available in the public domain and used often in polymorphism detection studies, such as the 1000 Genomes Project (Consortium, 2010), and hence are likely to have genotype information for them in publicly accessible databases such as dbSNP (Day, 2010). In addition, it is known that different sequencing platforms vary in their ability to identify deviant nucleotides, especially with respect to complex genomic regions, such as regions with highly repetitive DNA (Harismendy et al., 2009; Moore et al., 2011; Lam et al., 2012). A total of 4,596,517 variants among the 52 individuals (2921142 SNVs, 667458 insertions, 752180 deletions, and 255737 multinucleotide variants and rearrangements) were novel as defined. For each of the 24,277,549 non-reference variant sites, we identified the ancestral allele using the chimp and Macaque genome comparisons as described in the Section “Materials and Methods.” We could not determine the ancestral allele for 676,185 variants due to limitations in the available chimp and Macaque reference assemblies. This amounted to 2.78% of the total variants observed. We evaluated the likely functional effect of the derived alleles as described in the Methods and cataloged the number and rate of variant functional category types per-genome.

### General population comparisons

**Figure 1:**
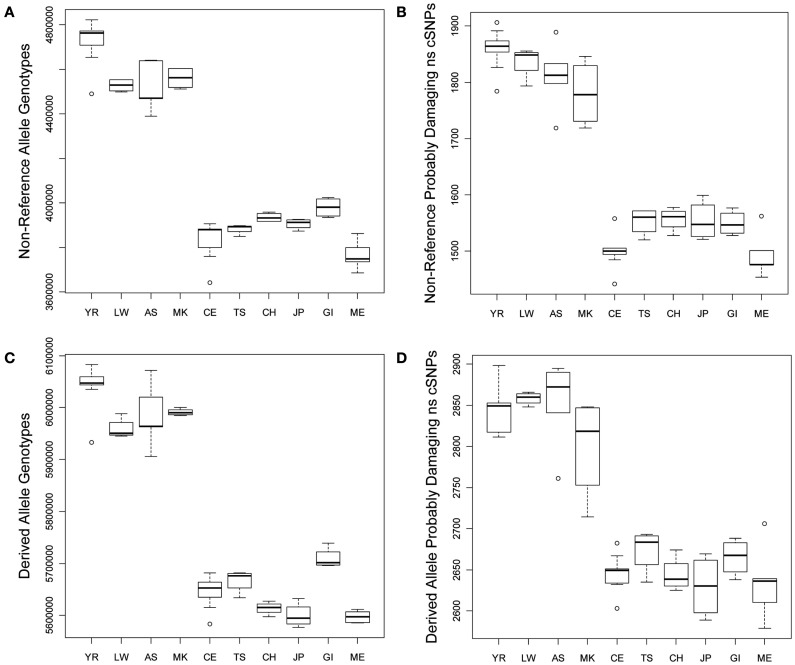
**Boxplots reflecting the differences in the number and rates of specific variant types across the 10 populations**. **(A)** Number of loci on individual genomes with at least one non-reference allele (i.e., homozygous or heterozygous non-reference allele genotypes); **(B)** Number of coding loci on individual genomes with at least one non-reference allele that results in a non-synonymous amino acid substitution that is predicted to have functional effect. **(C)** Number of loci on individual genomes with at least one derived allele (i.e., homozygous or heterozygous derived allele genotypes); **(D)** Number of coding loci on individual genomes with at least one derived allele that results in a non-synonymous amino acid substitution that is predicted to have functional effect.

We compared the frequency of variants in each of the defined functional categories across the 10 populations via graphical and linear regression analyses as described in the Section “Materials and Methods” and found very dramatic and statistically significant differences. Figure 1A provides a box plot depicting the differences in the absolute number of loci harboring non-reference alleles for each population. There are between 500,000–750,000 more loci with non-reference alleles in the genomes of African rather than non-African populations. Figure 1B depicts population differences in the number of probably damaging (PD) (by Polyphen2 designation) non-reference, non-synonymous coding SNVs (ns cSNVs; see Materials and Methods; Sunyaev et al., 2001). Each genome has, on average, 1650 loci that harbor a “PD” non-reference ns cSNVs according to Polyphen2, with Africans having ∼1.23 times more PD non-reference ns cSNVs than non-African populations (∼350 more ns cSNVs in absolute terms). Overall, we found that virtually all forms of functional non-reference variants that we have characterized are significantly more frequent in African rather than non-African populations (Tables S1 and S2 in Supplementary Material). We also determined the number of “novel” non-reference variants on each individual genome (i.e., variants only found on an individual genome in our dataset) by eliminating variants that were present on the other 51 genomes. We find that, on average, a human genome has ∼103,000 loci that harbor novel non-reference alleles, with non-African genomes harboring ∼10,000–50,000 less (Table S2 in Supplementary Material). We found consistency in the effect sizes and statistical significance of the African, European, and Asian populations, with some deviations from the East Indian (GI) and Mexican (ME) populations that likely reflect the unique population origins (Tables S1 and S2 in Supplementary Material).

As noted, due to the fact that the human reference genome available from the UCSC genome browser (Mangan et al., 2009) is based on the DNA from individuals of European ancestry, we did not want to rely on it for making claims about the frequency and rates of functional variants on genomes from individuals with different ancestries (see Materials and Methods; Boyko et al., 2008; Lohmueller et al., 2008). We therefore considered the frequency and rate of derived alleles across the genomes as a complement to comparisons involving non-reference alleles. Figures 1C,D depict the average number of derived variants on the genomes of individuals from the 10 different ancestral populations and the number of predicted PD derived ns cSNVs, respectively. Figure 1C suggests that African genomes possess ∼6,000,000 loci that harbor derived alleles whereas non-African genomes possess ∼350,000 less. This suggests that there are a great number of non-fixed derived variants in different human populations (i.e., variant sites for which ancestral and derived alleles are segregating in the human population at large). Figure 1D suggests that the number of loci that harbor PD derived ns cSNVs is ∼2850 in African genomes and ∼250 less in non-African genomes.

Table 1 presents the results of the regression analyses, and provides the estimated regression coefficients and their significance levels for each derived variant functional category. Note that since the YR African population was taken as the reference population, a negative regression coefficient means that genomes associated with a population have fewer variants, or a smaller per-genome rate, for a derived variant category than the YR population. The upper rows of Table 1 clearly suggest that there are a greater number of derived variants or alleles within African genomes across virtually every functional variant categories. The lower diagonal of Table 2 provides the results for analyses comparing the 10 populations on a pairwise basis for the total number of derived variants and suggests that although there are differences between populations in the same continent, they are not as pronounced as the differences between continental populations. The fact that we have relatively small sample sizes clearly affects this analysis. Tables S1–S6 in Supplementary Material provide more in-depth analysis results for each setting we considered.

**Table 1:** **Regression analysis results comparing the frequency and rates of variant types per-individual genome across 10 global populations**.

	*Y*-int	Africa			Europe		Asia		India	Mexico	*R*-Sqr
		LW	AS	MK	CE	TS	CH	JP	GI	ME	
Total derived genotypes	***6042271.00***	***−83419.25***	**−57488.00**	**−51149.25**	***−395516.33***	***−375283.25***	***−428364.00***	***−442378.00***	***−332701.50***	***−444921.80***	***0.97***
Non-sense SNPs:	***117.11***	7.14	3.09	**−**4.86	***−12.44***	**−11.61**	**−10.36**	**−12.11**	**−12.61**	**−**5.11	***0.45***
Frameshift structural variants:	***450.11***	**−**7.86	**−**3.51	**−**15.36	***−64.56***	**−36.11**	**−35.36**	**−37.61**	**−21.36**	***−55.11***	***0.71***
Splicing change variants:	***3009.33***	**−**17.08	17.47	9.42	4.33	14.92	**−**34.08	**−48.58**	**−**5.08	2.07	**0.29**
Probably damaging nscSNPs:	***2842.67***	15.83	9.13	**−42.92**	***−197.89***	***−168.92***	***−198.67***	***−213.17***	***−177.42***	***−208.67***	***0.90***
Possibly damaging nscSNPs:	***2201.44***	17.31	11.56	**−**13.19	***−148.00***	***−120.94***	***−150.19***	***−139.69***	***−143.19***	***−133.84***	***0.82***
Protein motif damaging variants:	***1012.22***	**−**16.72	**−**21.02	**−**17.72	***−111.44***	***−82.22***	***−89.47***	***−109.72***	***−69.47***	***−108.22***	***0.76***
TFBS disrupting variants:	***625.67***	***−92.67***	***−65.67***	***−39.42***	***−122.56***	***−117.67***	***−74.17***	***−80.67***	**−56.42**	***−155.67***	***0.75***
miRNA-BS disrupting variants:	***215.78***	**−**7.28	**−**10.78	**−**15.78	***−51.00***	***−42.53***	***−49.78***	***−51.78***	***−41.03***	***−66.58***	***0.91***
ESE-BS disrupting variants:	***2912.00***	**−**33.25	**−**6.80	**−**35.75	***−141.89***	***−138.50***	***−187.75***	***−163.25***	***−134.25***	***−140.00***	***0.82***
ESS-BS disrupting variants:	***1225.56***	**−**17.31	**−**1.36	15.19	***−43.56***	**−40.31**	***−54.06***	***−71.56***	**−40.31**	***−84.56***	***0.72***
Total likely functional variants:	***12983.11***	**−**148.11	**−**71.31	**−**133.11	***−730.11***	***−599.61***	***−729.61***	***−734.86***	***−557.86***	***−783.11***	***0.90***
Functional variants/total variants × 100000	**214.87**	0.53	0.86	**−**0.38	**2.12**	***3.65***	***3.40***	***3.86***	***2.75***	***3.09***	***0.57***
Total number of novel variants:	***230608.44***	19681.56	**−**16356.84	**−26810.44**	***−133947.00***	***−122674.44***	***−110435.44***	***−113591.94***	***−98801.19***	***−125688.44***	***0.92***
Total likely functional novel variants:	***798.78***	66.22	**−**22.18	**−125.03**	***−360.56***	***−290.78***	***−251.03***	***−263.78***	**−*250.03***	***−331.78***	***0.84***
Total number of homozygous genotypes:	***2919451.44***	2748.06	680.16	**30050.56**	***371821.44***	***362882.06***	***429429.81***	***426953.06***	***331099.31***	***377807.36***	***0.99***
Probably damaging hmz nscSNPs:	***985.00***	0.75	**−**20.80	**25.75**	***202.78***	***191.00***	***243.75***	***191.75***	***160.00***	***207.60***	***0.97***
Prob Dam hmz nscSNPs/Total Var iants × 100000	***16.30***	0.24	**−**0.19	**0.57**	***4.73***	***4.45***	***5.58***	***4.71***	***3.75***	***5.00***	***0.98***
Total likely functional homozygous variants:	***6103.11***	30.39	**−**19.71	62.14	***772.00***	***762.39***	***907.39***	***877.89***	***606.14***	***835.09***	***0.98***
Likely functional hmz variants/total Variants × 100000	***101.02***	**1.91**	0.64	**1.89**	***20.74***	***20.14***	***23.86***	***23.65***	***16.49***	***22.94***	***0.99***

*Note that the YR (Yoruban) sample was taken as the reference. *Y*-int = *Y*-intercept; *R*-Sqr = Fraction of Variation in the Variant type explained by the regression model. Bolded entries = *p*-value < 0.05; Italicized entries = *p*-value <  0.0005*.

**Table 2:** **Pairwise population comparisons using Tukey’s HSD method for the number of derived genotypes per-individual genome (below diagonal) and number of functional homozygous derived genotypes (above diagonal)**.

Sample	YR	LW	AS	MK	CE	TS	CH	JP	Gl	ME
YR		−30	20	−62	***−772***	***−762***	***−907***	***−878***	***−606***	**−*835***
LW	−**83419**		50	−32	***−742***	***−732***	***−877***	**−*848***	**−*576***	**−*805***
AS	−57488	25931		−82	***−792***	***−782***	***−927***	**−*898***	***−626***	**−*855***
MK	−51149	32270	6339		***−710***	***−700***	**−*845***	***−816***	**−*544***	***−773***
CE	**−*395516***	**−*312097***	**−*338028***	**−*344367***		10	**−135**	**−106**	*166*	−63
TS	**−*375283***	**−*291864***	**−*317795***	**−*324134***	20233		**−145**	−116	*156*	−73
CH	**−*428364***	**−*344945***	***−370876***	**−*377215***	−32848	−53081		30	*301*	72
JP	**−*442378***	**−*358959***	**−*384890***	**−*391229***	−46862	−67095	−14014		*272*	43
Gl	**−*332702***	**−*249282***	***−275214***	*281552*	62815	42582	**95663**	**109677**		**−229**
ME	**−*444922***	**−*361503***	**−*387434***	**−*393773***	−49405	−69639	−16558	−2544	**−112220**	

*Entries reflect the average differences with the populations listed in the second through eleventh columns subtracted from the populations listed in the first column. Bolded entries = *p*-value < 0.05; Bolded and Italicized entries = *p*-value < 0.0005*.

### Homozygous variant comparisons

**Figure 2:**
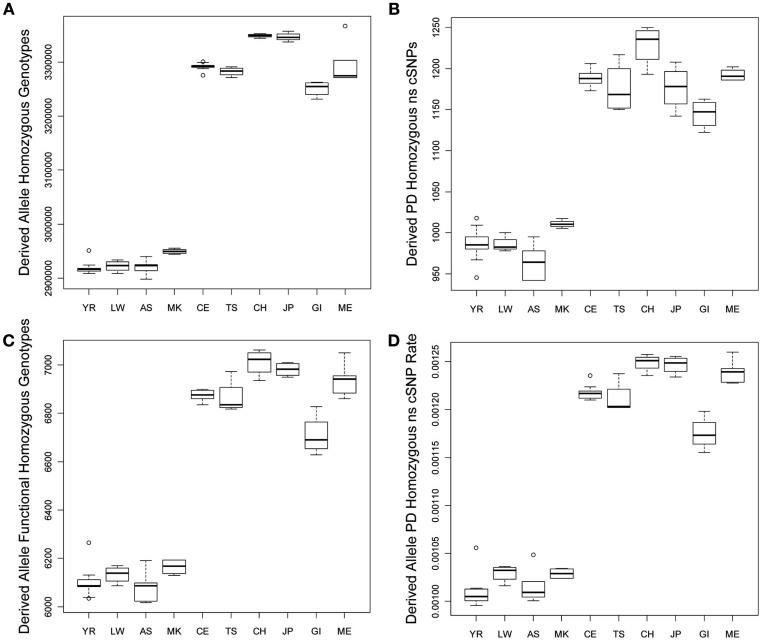
**Boxplots reflecting the differences in the number and rates of specific variant types across the 10 populations**. **(A)** Number of loci on individual genomes that are homozygous for a derived allele; **(B)** Number of coding loci on individual genomes that are homozygous for a derived allele that results in a non-synonymous amino acid substitution that is predicted to have functional effect. **(C)** Number of loci on individual genomes that are homozygous for a derived allele that is predicted to have a functional effect; **(D)** The rate of loci on individual genomes that are homozygous for a derived allele that is predicted to have a functional effect (relative to all loci on individual genomes with at least one derived allele).

We tested for differences in the frequency and per-genome rate of functional derived homozygous genotypes across the populations. Figure 2 provides a graphical display of the results. Figure 2A suggests that there is greater number of homozygous loci with derived alleles in non-African populations, and Figure 2B suggests that there are a greater number of homozygous loci with PD derived allele ns cSNVs in non-African populations as well. Figures 2C,D suggest that there are a greater number of homozygous loci with likely functional derived alleles of any type and ultimately a greater rate of homozygous loci with likely functional derived alleles across entire individual genomes, respectively. This result – that despite the fact that African genomes have a greater number of derived variants and derived functional variants, there is a greater number and rate of *homozygous* derived and homozygous derived functional variants among non-African genomes – is consistent with the findings of Bustamante and colleagues (Lohmueller et al., 2008). The bottom rows of Table 1 provide the regression analysis results for homozygous derived variants and clearly show that there is a significantly greater number and per-genome rate of homozygous functional derived variants in non-African populations.

Interestingly, although we found some evidence for consistency in the deviations of the non-Yoruban African and non-African populations from the Yoruban population with respect to numbers and rates of functional variants, there were more subtle, but statistically significant, differences in the total number and rates of different derived variant functional categories, including the number and rate of derived allele homozygous loci, between non-African populations (Table 2, contrast the entries above and below the diagonal). So, for example, the number of homozygous loci harboring derived, likely functional alleles differs between European and Asian as well as East Indian populations, but not necessarily between European populations and the admixed Mexican population (upper diagonal entries of Table 2).

### Population-specific variant comparisons

To further characterize the population-level differences in the functional content of individual genomes, we determined the number of population-specific variants in European, Asian, and African populations in a manner analogous to the approach taken by Lohmueller et al. (2008) as described in the Section “Materials and Methods.” Table 3 provides the summary information for the total number of population-specific variants as well as the per-variant rate of different functional variant categories for each population. The *z*-tests assessing the equality of derived functional variant category frequencies are also provided in Table 3. As can be seen, there are significantly higher rates of population-specific likely functional derived variants per-genome across virtually all functional variant categories in European and Asian populations relative to the African population, despite there being more population-specific variation within the African population (top row). However, there are virtually no significant differences in these rates between European and Asian populations (Table 3, last column).

**Table 3:** **Frequency and rates (×10000) of population-specific variant types for African (AFR), European (EUR), and Asian (ASN) populations**.

Variant type	Populations			*z*-test *p*-values		
	AFR	EUR	ASN	AFR vs. EUR AFR vs. ASN EUR vs. ASN		
Total number of variants:		7614850	2024886	1294731
Non-sense SNPs rate	0.500	0.840	0.842	6.931E-09	6.329E-07	4.910E-01
Frameshift structural variants rate	1.663	3.008	2.989	1.597E-34	6.239E-25	4.621E-01
Frameshift insertion rate	0.657	1.274	1.383	6.368E-19	1.089E-18	2.006E-01
Frameshift deletion rate	0.879	1.417	1.352	3.877E-12	1.584E-07	3.102E-01
Frameshift rearrangement rate	0.127	0.316	0.255	2.614E-09	2.228E-04	1.572E-01
Splicing change variants rate	1.707	2.514	2.379	4.655E-14	7.112E-08	2.223E-01
Probably damaging nscSNPs rate	10.103	15.472	15.602	1.136E-91	4.578E-69	3.853E-01
Possibly damaging nscSNPs rate	5.991	7.744	8.233	7.313E-19	3.064E-21	6.111E-02
Protein motif damaging variants rate	4.104	6.311	6.581	2.612E-39	3.043E-35	1.726E-01
TFBS disrupting variants rate	2.793	4.173	4.063	7.493E-69	2.764E-42	1.785E-01
miRNA-BS disrupting variants rate	0.948	1.170	1.081	2.405E-03	7.715E-02	2.286E-01
ESE-BS disrupting variants rate	5.835	7.260	7.283	1.696E-13	2.840E-10	4.689E-01
ESS-BS disrupting variants rate	2.460	3.013	2.865	6.435E-06	3.539E-03	2.232E-01
Total likely functional variant rate	23.718	34.906	35.436	8.999E-170	1.234E-132	2.128E-01

As noted, in addition to comparing population summaries, we also determined the rate of population-specific, likely functional variants in each individual genome within each population. This is important since sample size differences could impact the ability to identify and test frequency differences of rare and population-specific variants if only population summary statistics over all the genomes are considered, as in Table 3. We find that there are higher rates of functional variants among the population-specific variants within European and Asian genomes relative to African genomes despite the fact the rate of such variants is higher across all variants (i.e., not just population-specific variants) in African rather than European and Asian genomes (Table S6 in Supplementary Material).

### Simulation study results using known pathogenic variants

As emphasized in the Introduction, two factors go into the inference that a variant is likely to be pathogenic and causative of an idiopathic condition: the variant must be unique to the patient with the condition (i.e., “novel”) and it must be predicted to be functional. Determining the novelty of a variant requires contrasting the patient’s genomic variants with variants on other individuals’ genomes (i.e., a reference set of genomes). Determining functionality requires the use of bioinformatics techniques, if not direct laboratory-based functional assays. Thus, in order to determine the likely impact of our findings on searches for pathogenic variants influencing idiopathic diseases, we considered how many ns cSNVs in five target individuals’ genomes (i.e., a European, African, Mexican, East Indian, and Puerto Rican simulated patient’s genome) would be considered as likely pathogenic beyond known dominant-acting CMT syndrome-inducing variant and recessive-acting CF-inducing variants when compared to different reference panel genome’ ns cSNV lists derived from the 52 individuals for which we had WGS information (see Materials and Methods). We also considered the use of reference sets made up of data from the 1000 genomes project^3^.

We computed Polyphen2, SIFT, and the average Polyphen2 and SIFT scores for the CMT and CF variants, all ns cSNVs variants in each of the five target individual’s genomes and all ns SNVs in each reference data set. We limited our assessment to cSNVs due to the low coverage sequencing in non-coding regions pursued in the 1000 Genomes project. Table 4 provides the number of variants that would be considered both novel and as having a predicted functional effect score at least a large as the known disease-causing variants relative to all variants. Table 4 only provides the results of our analyses when considering the dominant-acting CMT mutation as the pathogenic variant to be identified. The upper rows consider analyses that only use Polyphen2 scores, the middle rows the use of SIFT scores, and the bottom rows the use of the average Polyphen2/SIFT scores as a way of assessing the functional effects of the ns cSNVs. The columns correspond to the use of different reference variant sets for determining the novelty of a variant. Note that since the non-European target individuals we assessed were part of the 69 WGS individuals that we studied we could not consider the use of a combined reference set with 1000 Genomes and the 69 WGS genomes data (i.e., the “ALLDB” column of Table 4). From Table 4, it can be seen that one could expect some 194 ns cSNVs to be called as “novel” that have Polyphen2 scores greater (and hence likely to be functional) than the known CMT mutation for the European individual we studied based on the use of a 1000 Genomes-derived European reference ns cSNV panel; 680 if a 1000 Genomes-derived African reference panel is used; and 439 if an eight member European reference panel was constructed from the ns cSNVs from the WGS data we studied. These would be out of a total of 1539 ns cSNVs for this European individual. These numbers represent the number of “false leads” one would have to deal with in trying to identify the known causative variant (i.e., the “implanted” CMT variant).

**Table 4:** **The number of ns cSNVs deemed “novel” with predicted functional consequence scores greater than that assigned a known CMT syndrome-inducing variant as function of the reference panel used for five individual genomes of diverse ancestry**.

Indiv	Source	ALLDB	Polyphen score >0.825 (*N* = 506)							
			All 1000k	1000k EUR	1000k ASN	1000k AFR	All CGI	CGI EUR	CGI ASN	CGI AFR
			1000+	∼500	∼500	∼500	52	8	8	8
European	STSI	101/1593	179/1593	194/1593	317/1593	680/1593	174/1593	439/1593	588/1593	669/1593
African American	CGI		185/1938	262/1938	795/1938	1068/1938		991/1938	995/1938	568/1938
Mexican	CGI		169/1595	272/1595	362/1595	676/1595		491/1595	566/1595	681/1595
East Indian	CGI		257/1685	329/1685	405/1685	669/1685		533/1685	538/1685	680/1685
Puerta Rican	CGI		220/1664	287/1664	417/1664	683/1664		491/1664	576/1664	654/1664

**Indiv**	**Source**	**ALLDB**	**Sift score >0.931 (*N* = 506)**							
			**All 1000k**	**1000k EUR**	**1000k ASN**	**1000k AFR**	**All CGI**	**CGI EUR**	**CGI ASN**	**CGI AFR**
			***n* = 1000+**	**∼500**	**∼500**	**∼500**	**52**	**8**	**8**	**8**

European	STSI	127/2593	232/2593	252/2593	394/2593	947/2593	221/2593	627/2593	843/2593	930/2593
African American	CGI		241/3199	319/3199	1174/3199	1662/3199		1557/3199	1569/3199	828/3199
Mexican	CGI		221/2565	311/2565	449/2565	963/2565		689/2565	831/2565	948/2565
East Indian	CGI		353/2856	433/2856	537/2856	1070/2856		803/2856	835/2856	1016/2856
Puerta Rican	CGI		289/2657	351/2657	502/2657	963/2657		676/2657	809/2657	900/2657

**Indiv**	**Source**	**ALLDB**	**Sift score >0.878 (*N* = 506)**							
			**All 1000k**	**1000k EUR**	**1000k ASN**	**1000k AFR**	**All CGI**	**CGI EUR**	**CGI ASN**	**CGI AFR**
			***n* = 1000+**	**∼500**	**∼500**	**∼500**	**52**	**8**	**8**	**8**

European	STSI	90/1269	157/1269	169/1269	268/1269	563/1269	146/1269	362/1269	478/1269	551/1269
African American	CGI		148/1524	197/1524	628/1524	871/1524		800/1524	802/1524	466/1524
Mexican	CGI		145/1229	204/1229	279/1229	556/1229		396/1229	455/1229	565/1229
East Indian	CGI		213/1321	255/1321	315/1321	562/1321		428/1321	429/1321	556/1321
Puerta Rican	CGI		173/1281	208/1281	309/1281	546/1281		391/1281	461/1281	531/1281

*The numerator in each cell entry provides the number of ns cSNVs with functional consequence scores greater than the average of (*N* = 506) known CMT mutations that would be deemed novel on the basis of the different reference panels associated with each column of the table for the individuals’ whole genome variant lists denoted in the “Indiv” column. The denominator provides the total number of ns cSNVs on each individual’s (“Indiv”) genome with scores higher than the CMT mutation*.

Table 4 also suggests that the use of different algorithms for predicting the likely functional significance of variants makes a difference (contrast the entries between the top, middle, and bottom sets of rows), possibly the use of sequencing platforms (as indicated by the small decrease in false positive results from the use of the 1000 Genomes reference panels vs. the only eight member WGS panel provided by the CGI data) and most importantly the genetic background of the members in the panel (i.e., contrast the columns that only consider the eight member panels derived from the WGS data). We saw similar results when assessing the novelty of homozygous variants and the scoring of the likely functional significance of the known CF mutation (Table S7 in Supplementary Material).

**Figure 3:**
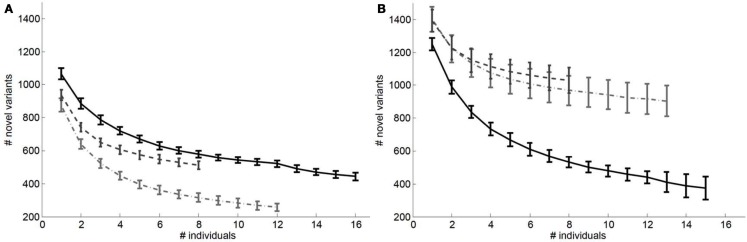
**Relationship between the number of ns cSNVs with polyphen 2.0 scores >0.8 that would be declared as novel if a European individual’s ns cSNVs were compared to a reference panel made up of European, African or Asian individuals (A) or if an African individual’s ns cSNVs were compared to a reference panel made up of European (light dashed and dotted line), African (black solid line), or Asian individuals (dashed light line) (B) as a function of the number of individuals in the panel**. Standard errors were computed by taking a randomly choosing the number of individuals from our collection of European, African, and Asian genomes given on the *x* axis.

**Figure A3:**
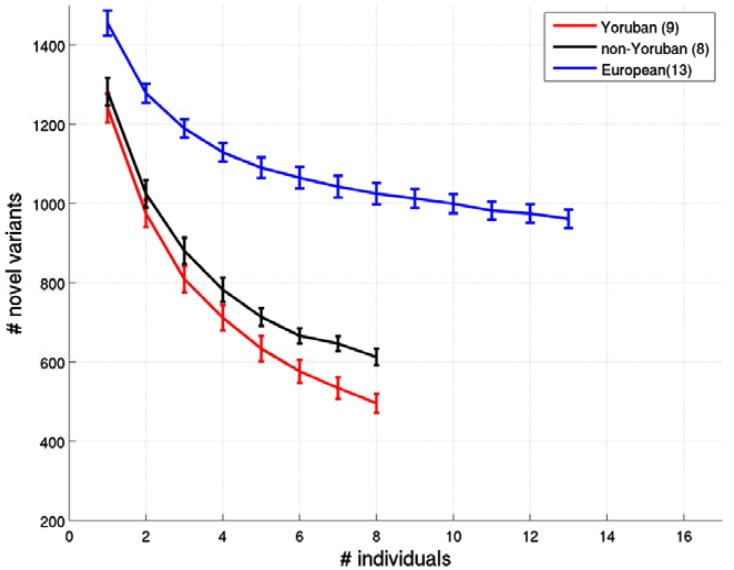
**Relationship between the number of ns cSNVs with polyphen 2.0 scores > 0.8 that would be declared as novel an African individual’s ns cSNVs were compared to a reference panel made up of European, African Yoruban or African non-Yoruban individuals as a function of the number of individuals in the panel**. Standard errors were computed by taking a randomly choosing the number of individuals from our collection of European, African Yoruban, or African non-Yoruban individuals given on the *x* axis.

We also considered the impact of the addition of genomes to a reference panel on potential “false lead” rates in pathogenic variant identification. Figure 3 depicts the relationship between the number of variants with Polyphen2 scores greater than 0.8 that would be determined as novel on a European (Figure 3A) and African genome (Figure 3B) if reference panels were comprised of increasing numbers of European, African, and Asian individuals. It is quite clear from Figure 3 that including individuals with appropriate genetic backgrounds in reference panels for determining the novelty of variants is crucial for reducing false leads and appropriately ranking likely pathogenic variants. We found similar patterns when considering analyses of an African individual’s genome-wide ns cSNVs when using different (within) African population reference panels (Figure A3 in Appendix) but with a lesser overall effect than if non-African individuals are used to construct the reference panel.

## Discussion

We have assessed the differences in the genome-wide rates of DNA sequence variants associated with different genomic functional elements across 10 contemporary global populations. We find striking evidence that historical population-level phenomena of whatever sort, including possibly bottlenecks, unique migratory patterns, admixture, natural selection, and random drift, have left an imprint on the standing genetic variation that is likely to influence phenotypic expression in these populations. In this light our results are consistent with previous reports (e.g., Lohmueller et al., 2008), but extend them to the entire genomes of individuals from many different global populations. We also considered many important functional variant categories and used genomes sequenced on a single platform and to great depth (∼60×). Importantly, we find that, on an individual genome-wide basis, there is both an absolute and proportionately greater number and rate of loci that are homozygous for derived alleles that are likely to be functional in non-African populations (Lohmueller et al., 2008).

Our findings suggest that WGS will not only be of tremendous value in future population genetic and human evolutionary studies, but also that global human population differences in rates of novel, deleterious, or functional variants must be taken into account in certain clinical sequencing applications. Importantly, our results emphasize the need for care in evaluating the novelty or likely functional impact of variants in clinical sequencing studies focusing on the identification of disease-inducing “pathogenic” variants in an individual genome based on comparisons of that genome to a reference panel of genomes (Biesecker et al., 2009). This is the case because of the tremendous diversity of variants across human populations, the existence of an abundance of likely functional variants that are population-specific, and population differences in the absolute number and rates of homozygous variants that are likely to impact phenotype expression. Thus, for example, it might be highly problematic to evaluate the novelty of variants in the genome of an African patient in order to filter out variants not likely to cause his or her unique disease by comparing that individual’s genome to a reference panel that only includes genomes from individuals with European ancestry. This problem might be particularly pronounced in large urban centers where individuals with a wide variety of ancestries may require medical care.

There are a number of issues associated with our analyses that require further consideration. First, although we used state-of-the-field tools for assessing the potential molecular functional impact of DNA sequence variations, many of these tools are not optimal nor completely accurate and therefore require improvement (Plumpton and Barnes, 2007; MacArthur et al., 2012). Despite this, the consistency of the patterns we find with their use across all functional categories is very unlikely to represent an artifact induced by a drastic misclassification of the functional impact of the variants. Second, although we avoided complete reliance on a human reference genome for studying differences in variant types and rates across populations by determining ancestral and derived alleles, many functional elements and functional prediction algorithms rely on the available reference genome (e.g., defining TFBS and predicting TFBS disrupting variants) and hence may not be adequately evaluating the functionality of specific variants due to structural and sequence context differences that exist between individuals (Balasubramanian et al., 2011). However, as SIFT and Polyphen2 exploit cross-species nucleotide conservation information, we are confident that our analyses of coding variants is not affected as much by this issue. Third, we defined derived and ancestral alleles using the available chimp and macaque reference genomes (Boyko et al., 2008; Lohmueller et al., 2008). These reference genomes – like all species reference genomes – are likely incomplete and harbor some level of inaccurate nucleotide assignments. In addition, polymorphism among individual chimps and macaques are not reflected in the chimp and macaque reference genomes, making it hard to know which alleles at these polymorphic sites actually reflect the consensus ancestral allele for the reference. Thus, some of the variants we characterized as derived or ancestral may be inaccurate, but not likely to a degree that would invalidate our results. Fourth, importantly, we did not consider phase information when evaluating the functional content of the human genomes we analyzed, as phase information is currently hard to obtain without additional resources (e.g., family members of individuals sequenced; Tewhey et al., 2011). Thus, we were not in a position to evaluate the likely impact or population differences of potentially functional compound heterozygous sites in the human genome – sites which may be of particular relevance for human phenotypic expression and disease studies (Tewhey et al., 2011). Fifth, we had relatively small sample sizes (e.g., 4–5 genomes from some populations) clearly limiting our ability to detect subtle variant frequency differences and make broad generalizations about variant frequencies in the population at large.

Despite the shortcomings of our study, we believe studies such as ours will help usher in an era of routine WGS for human population and clinical studies. In order to fully develop such studies, however, greater emphasis on the construction of ubiquitous and accessible whole genome reference sequence databases must be made, with sensitivity to the need to populate those databases with genomic information from individuals with different ancestries (Bustamante et al., 2011). The number of individuals needed of each ancestry to be reliable for determining the likelihood that a particular variant is unique to a patient with an idiopathic condition is an open question, but our analyses, as well as other recent studies (Pelak et al., 2010), suggest that there is diminishing returns in adding more and more genomes to a reference panel in order to cut down on the number of variants falsely inferred as novel, possibly after as few as 8–15 individual genomes. In addition, better methods for predicting the functional consequences of variants of unknown significance are needed, as are methods for leveraging such predictions in more sophisticated pathogenic variant identification strategies (Ionita-Laza et al., 2011; Rope et al., 2011; Torkamani et al., 2011; Yandell et al., 2011).

Our results also bear on DNA sequence-based searches for rare variants that contribute to non-idiopathic common, chronic conditions such as diabetes, cancer, and heart disease (Bansal et al., 2010). For example, many statistical methods for testing the contribution of rare variants to a disease weight variants by their frequency and their likely functional impact score, both activities of which should be informed by the knowledge of genome-wide rates of variants across populations and the behavior of bioinformatics tools for assessing variant functionality. In this light, our study may motivate larger and more sophisticated studies investigating the impact of population-level genetic phenomena on the utility of, and necessary infrastructure for, clinical DNA sequencing.

## Conflict of Interest Statement

The authors declare that the research was conducted in the absence of any commercial or financial relationships that could be construed as a potential conflict of interest.

## Supplementary Material

The Supplementary Material for this article can be found online at: http://www.frontiersin.org/Applied_Genetic_Epidemiology/10.3389/fgene.2012.00211/abstract

Supplementary Data Sheet S1 **Regression analysis results for reference-based variants: all variants**.

Supplementary Data Sheet S2 **Regression analysis results for reference-based variants: novel variants**.

Supplementary Data Sheet S3 **Regression analysis results for ancestral allele-based variants: all variants**.

Supplementary Data Sheet S4 **Regression analysis results for ancestral allele-based variants: all homozygous variants**.

Supplementary Data Sheet S5 **Regression analysis results for ancestral allele-based variants: novel variants**.

Supplementary Data Sheet S6 **Regression analysis results for ancestral allele-based population-specific variants: all variants**.

Supplementary Data Sheet S7 **False positive variants associated with the identification of a homozygous CF mutation if that homozygous CF mutation had a pathogenicity score greater than the average Polyphen2, SIFT or average Polyphen2/SIFT scores for 567 known CF mutations**.

## Footnotes

^1^http://www.completegenomics.com/sequence_data/download-data/

^2^www.1000genomes.org/

^3^www.1000genomes.org/

## Appendix

### Human population differences in rates

#### Ancestry assessment

We assessed the genetic background similarity of the individuals by constructing identity-by-state (IBS) allele sharing similarity matrices using 16,411 markers which had also been genotyped on 4,123 individuals in various public domain databases for which ancestry information on the individuals in those databases was recorded. We also calculated IBS allele sharing matrices based on 19,208,882 variants determined from the whole genome sequencing for the 52 individuals in addition to the parents in the Puerto Rican trio. We then applied multidimensional scaling (MDS) analysis to the sharing matrices to determine patterns in genetic background similarity of the individuals (Reich et al., 2008). Figure A1 in Appendix depicts the first two axes for the allele sharing determined through the use of the 16,411 markers genotyped on the 4,123 reference individuals as well. Figure A2 in Appendix depicts the first two PCs the allele sharing determined through the use of the 19,208,882 markers identified in the sequencing of the genomes of the 52 + 2 individuals.

#### Functional annotations

##### Genomic elements and conservation

All variants were associated with conservation information in two ways. First, variants were associated with conserved elements from the phastCons conserved elements (28 way, 44 way, 28 way Placental, 44 way Placental, and 44 way Primates; Siepel et al., 2005). These conserved elements represent potential functional elements preserved across species. Conservation is also assessed at the specific nucleotide positions impacted by the variant using the phyloP method (Pollard et al., 2010). The same conservation levels as phastCons are used in order to gain higher resolution into the potential functional importance of the specific nucleotide impacted by the variant.

##### Transcription factor binding sites and predictions

All variants, regardless of their genomic position, were associated with predicted transcription factor binding sites (TFBS) and scored for their potential impact on transcription factor binding. We pre-computed predicted TFBS by utilizing the human transcription factors listed in the JASPAR and TRANSFAC transcription factor binding profile to scan the human genome using the MOODS algorithm (Wingender et al., 1996; Wasserman and Sandelin, 2004; Korhonen et al., 2009). The probability that a site corresponds to a TFBS is calculated by MOODS based on the background distribution of nucleotides in the human genome. We call TFBS at a relaxed threshold within (*p*-value < 0.0002) in conserved, hypersensitive, or promoter regions, and at a more stringent threshold (*p*-value < 0.00001) for all other locations in order to capture sites that are more likely to correspond to true functional TFBS. Conserved sites correspond to the phastCons conserved elements, hypersensitive sites correspond to Encode DNASE hypersensitive sites annotated in UCSC genome browser, while promoters correspond to regions annotated by TRANSPro, and 2 kb upstream of known gene transcription start sites, identified by SwitchGear Genomics ENCODE tracks. The potential impact of variants on TFBS were scored by calculating the difference between the mutant and wild-type sequence scores using the position weighted matrix method described in Stormo (2000) and shown to identify regulatory variants in Andersen et al. (2008).

##### Splicing predictions

Variants falling near exon-intron boundaries were evaluated for their impact on splicing by the maximum entropy method of maxENTscan (Yeo and Burge, 2004). Maximum entropy scores were calculated for the wild-type and mutant sequence independently, and compared to predict the variants impact on splicing. Changes from a positive wild-type score to a negative mutant score suggest a splice site disruption. Variants falling within exons were also analyzed for their impact on exonic splicing enhancers and/or silencers (ESE/ESS). We determine the number of ESE and ESS sequences created or destroyed based on the hexanucleotides reported as potential exonic splicing regulatory elements (Stadler et al., 2006) and shown to be the most informative for identification of splice-affecting variants (Woolfe et al., 2010).

##### microRNA binding sites

Variants falling within 3′UTRs were analyzed for their impact on microRNA binding in two different manners. First, all 3′UTRs were associated with pre-computed microRNA binding sites using the targetScan algorithm and database (Lewis et al., 2005). Variant 3’UTR sequences were rescanned by targetScan in order to determine if microRNA binding sites are lost due to the impact of the variation. Second, the binding strength of the microRNA with its wild-type and variant binding site is calculated by the RNAcofold algorithm to return a ΔΔ*G* score for the change in microRNA binding strength induced by introduction of the variant (Hofacker, 2003).

##### Protein coding variants

While interpretation of frameshift and nonsense mutations is fairly straightforward, the functional impact of non-synonymous changes and in-frame indels or multi-nucleotide substitutions is highly variable. We utilized the PolyPhen-2 algorithm, which performs favorably in comparison to other available algorithms, for prioritization of non-synonymous single nucleotide substitutions (Adzhubei et al., 2010). A major drawback to predictors such as PolyPhen-2 is the inability to address more complex amino acid substitutions. To address this issue, we also generated the LogR. *E*-value score of variants, which is the log ratio of the *E*-value of the HMMER match of PFAM protein motifs between the variant and wild-type amino acid sequences (Clifford et al., 2004). This score has been shown to be capable of accurately identifying known deleterious mutations. More importantly, this score measures the fit of a full protein sequence to a PFAM motif, therefore multi-nucleotide substitutions are capable of being scored by this approach.
