# Supplementary material for: Clinical Implications of Human Population Differences in Genome-Wide Rates of Functional Genotypes
Source: Front Genet. 2012 Nov 1;3:211. doi: 10.3389/fgene.2012.00211 (PMC3485509; doi:10.3389/fgene.2012.00211)
Supplement: Supplementary Data Sheet S1 — Regression analysis results for reference-based variants: all variants. [file 32001_Schork_DataSheet1.PDF]

Reference-Based Variants: All Variants
Row 1: Regression Coefficients
Row 2: P-values for Regression Coefficients

	Var Cat	Y-int	LWK	ASW	MKK	CEU	TSI	CHB	JPT	GIH	MEX	F-Stat	P-Value	R-Sqr
Coding SNPs:	1	25758	-707.25	-875	-762	-4890.6667	-4462	-4307	-4313.75	-3771.5	-4997.4	160.9211	0	0.965211
	1	0	0.008531	0.000581	0.004764	0	0	0	0	0	0			
Nonsynonymous SNPs:	2	11983.7778	-297.028	-411.378	-345.278	-2256.5556	-2018.53	-1949.28	-1914.28	-1736.53	-2333.38	130.6527	0	0.957494
	2	0	0.028999	0.001417	0.011642	0	0	0	0	0	0			
Synonymous SNPs:	3	13667.1111	-413.861	-465.511	-410.111	-2617.4444	-2429.36	-2341.61	-2382.86	-2018.61	-2654.31	171.7324	0	0.96733
	3	0	0.003281	0.000444	0.003556	0	0	0	0	0	0			
Nonsense SNPs:	4	107.111111	3.638889	1.888889	-6.61111	-16.666667	-14.1111	-16.1111	-16.6111	-16.3611	-9.71111	6.643385	9E-07	0.533889
	4	0	0.464981	0.682404	0.186358	5.8622E-05	0.005808	0.001787	0.001312	0.001532	0.03834			
Untranslated Region SNPs:	5	38208	-917	-1191	-1319.25	-7607.1111	-7090.25	-7018.75	-7071.5	-6273	-7828.6	301.7792	0	0.981143
	5	0	0.003698	7.79E-05	5.14E-05	0	0	0	0	0	0			
Non-coding RNA SNPs:	6	1198072.11	-33514.1	-38464.1	-33877.1	-214935.56	-202680	-192149	-199256	-183974	-224740	266.6252	0	0.97871
	6	0	0.000477	2.39E-05	0.000419	0	0	0	0	0	0			
Intronic SNPs:	7	1672861.33	-40257.8	-51920.1	-54030.8	-322929.11	-303577	-298376	-303519	-276284	-325826	455.8505	0	0.987436
	7	0	0.000294	1.35E-06	2.7E-06	0	0	0	0	0	0			
Intergenic SNPs:	8	2385485	-63218.8	-77790.8	-72154	-442040.11	-421971	-398267	-406666	-373981	-460679	279.7645	0	0.979689
	8	0	0.00099	2.18E-05	0.000202	0	0	0	0	0	0			
**Total SNPs:**	9	4130472	-105296	-132028	-128445	-778945.44	-738542	-709291	-722978	-661545	-800823	351.1354	0	0.983751
	9	0	0.000525	5.86E-06	3.38E-05	0	0	0	0	0	0			
Coding Insertions:	10	273.111111	-31.6111	-24.5111	-22.8611	-51.111111	-30.3611	-25.6111	-26.8611	-26.1111	-53.9111	8.094044	1E-07	0.582555
	10	0	0.001866	0.008625	0.02212	5.712E-09	0.002736	0.010736	0.007599	0.009362	1.07E-07			
In-frame Insertions:	11	32	-6	-4.8	-5	-7.8888889	-7.25	-7	-5.75	-5	-10	2.959256	0.004512	0.337843
	11	0	0.033172	0.065219	0.074404	0.00052258	0.010639	0.013502	0.040951	0.074404	0.000221			
Out-of-frame Insertions:	12	63.1111111	-15.3611	-8.51111	-7.11111	-9.6666667	-4.86111	-8.36111	-7.86111	-6.36111	-16.1111	4.332413	0.000155	0.42758
	12	0	0.000102	0.016227	0.060095	0.0014862	0.195566	0.02783	0.038227	0.091755	1.51E-05			
Frameshift Insertions:	13	178	-10.25	-11.2	-10.75	-33.555556	-18.25	-10.25	-13.25	-14.75	-27.8	7.895485	1E-07	0.576503
	13	0	0.109112	0.060209	0.0932	3.798E-09	0.00517	0.109112	0.039582	0.022453	1.14E-05			
Untranslated region Insertions:	14	2714.44444	-401.694	-270.644	-109.194	-484.88889	-414.444	-359.444	-382.944	-303.444	-644.444	15.99888	0	0.733931
	14	0	2.68E-06	0.000405	0.167806	3.7E-11	1.44E-06	2E-05	6.62E-06	0.000245	1E-12			
Non-coding RNA Insertions:	15	68212.8889	-11068.6	-8316.69	-3727.89	-11889.667	-10540.1	-7882.89	-9827.14	-8033.39	-17279.3	13.54444	0	0.700172
	15	0	3.07E-06	0.000105	0.090882	1.682E-09	7.68E-06	0.000554	2.56E-05	0.000442	2E-12			
Intronic Insertions:	16	102055	-17124	-13081.2	-6216.25	-18983.111	-16721	-12937	-15835.3	-13114	-26899.4	14.66432	0	0.71658
	16	0	1.75E-06	5.41E-05	0.061392	2.82E-10	2.8E-06	0.000184	7.78E-06	0.000153	1E-12			
Intergenic Insertions:	17	133432.444	-22078.7	-16618	-7541.19	-24211	-21479.4	-15833.7	-19611.9	-16349.9	-34812	14.35839	0	0.712279
	17	0	2.21E-06	8.05E-05	0.081421	5.62E-10	3.78E-06	0.000417	1.93E-05	0.000279	1E-12			
**Total Insertions:**	18	238844.222	-39671	-30030.6	-13903.5	-43793.556	-38708.7	-29214	-35916.5	-29842.2	-62497.8	14.55006	0	0.714989
	18	0	1.96E-06	6.76E-05	0.072251	3.82E-10	3.18E-06	0.000278	1.25E-05	0.000211	1E-12			
Coding Deletions:	19	298.777778	-25.7778	-17.5778	-18.0278	-58.555556	-49.5278	-39.2778	-35.7778	-23.2778	-72.9778	17.45262	0	0.750566
	19	0	0.005007	0.036652	0.046347	4E-12	4.75E-07	3.67E-05	0.000146	0.01084	1E-12			
In-frame Deletions:	20	29	-0.25	-2.4	-2.25	-4.5555556	-5.5	-3.75	-2.25	1.5	-7.8	5.039055	2.98E-05	0.464898
	20	0	0.894881	0.174706	0.236805	0.00299279	0.004792	0.050731	0.236805	0.428959	3.23E-05			
Inter-Codon Deletions:	21	56.3333333	-8.33333	-5.53333	1.166667	-11.111111	-12.5833	-4.58333	-5.83333	-5.33333	-14.1333	5.064933	2.81E-05	0.466173
	21	0	0.022166	0.098529	0.744033	0.00017166	0.000742	0.202157	0.105829	0.138615	6.1E-05			
Frameshift Deletions:	22	213.444444	-17.1944	-9.64444	-16.9444	-42.888889	-31.4444	-30.9444	-27.6944	-19.4444	-51.0444	15.68559	0	0.730052
	22	0	0.012206	0.124259	0.013461	1.1E-11	1.29E-05	1.69E-05	9.62E-05	0.004859	9E-12			
Untranslated region Deletions:	23	2876.55556	-367.556	-299.356	-210.556	-634.33333	-561.056	-487.306	-536.806	-489.306	-810.156	29.97501	0	0.837876
	23	0	3.72E-06	3.63E-05	0.005202	0	8.4E-11	5.5E-09	3.35E-10	4.91E-09	0			
Non-coding RNA Deletions:	24	75361.3333	-11702.3	-9268.13	-4339.08	-14576	-13400.6	-10139.8	-12077.6	-10991.3	-20139.9	16.55703	0	0.740574
	24	0	4.21E-06	6.19E-05	0.067582	2.9E-11	2.52E-07	4.89E-05	2.28E-06	1.31E-05	0			
Intronic Deletions:	25	112997.667	-18052.9	-14453.9	-7579.67	-23045.444	-21232.7	-16771.9	-19481.7	-17186.4	-31497.1	18.17526	0	0.758084
	25	0	2.22E-06	3.13E-05	0.033219	4E-12	6.16E-08	8.88E-06	4.54E-07	5.7E-06	0			
Intergenic Deletions:	26	148244.111	-23582.6	-18928.9	-9260.61	-29448.556	-27056.4	-20656.9	-24532.9	-22056.9	-40817.7	17.57143	0	0.751834
	26	0	2.41E-06	3.21E-05	0.046848	1E-11	1.24E-07	2.61E-05	1.09E-06	8.49E-06	0			
**Total Deletions:**	27	264800.444	-42061.9	-33716	-17089.7	-53258.222	-48981.4	-38011.9	-44644.9	-39811.4	-73283.4	17.99859	0	0.756288
	27	0	2.27E-06	3.13E-05	0.039347	6E-12	8.07E-08	1.47E-05	6.66E-07	6.47E-06	0			
Coding rearrangements:	28	321.111111	-45.6111	-22.9111	-23.3611	-44.111111	-23.1111	-19.3611	-30.6111	-19.8611	-68.7111	8.34389	0	0.589929
	28	0	8.2E-05	0.026413	0.035282	2.2799E-06	0.037224	0.079491	0.006391	0.072198	3.33E-09			
In-frame rearrangements:	29	298.222222	-42.9722	-25.8222	-25.9722	-42.111111	-25.9722	-22.7222	-29.7222	-20.7222	-65.8222	8.433708	0	0.592517
	29	0	6.6E-05	0.007532	0.012307	1.3012E-06	0.012307	0.027677	0.004445	0.044002	1.37E-09			
Frameshift rearrangements:	30	22.8888889	-2.63889	2.911111	2.611111	-2	2.861111	3.361111	-0.88889	0.861111	-2.88889	2.958553	0.00452	0.33779
	30	0	0.210449	0.137588	0.215234	0.22613053	0.174971	0.111988	0.671531	0.681209	0.140568			
Untranslated region rearrangements:	31	733.777778	-82.2778	-65.3778	-39.2778	-165	-127.028	-112.778	-117.528	-107.028	-185.578	47.53935	0	0.891262
	31	0	1.91E-07	4.88E-06	0.007204	0	0	2.8E-11	7E-12	1.53E-10	0			
Non-coding RNA rearrangements:	32	27531.1111	-2876.86	-2068.31	-1333.36	-4789.5556	-4076.36	-3356.61	-4017.61	-3432.36	-6181.51	24.07253	0	0.805842
	32	0	1.67E-05	0.000616	0.035114	0	8.66E-09	8.96E-07	1.27E-08	5.57E-07	0			
Intronic rearrangements:	33	35968.1111	-3787.36	-2911.11	-2049.36	-7076.4444	-6249.61	-5480.86	-6071.11	-5295.86	-8595.31	35.83593	0	0.860697
	33	0	2.75E-06	6.96E-05	0.007212	0	4E-12	2.75E-10	1E-11	7.75E-10	0			
Intergenic rearrangements:	34	52917.1111	-5468.11	-4030.31	-2597.86	-9728.5556	-8429.36	-6916.36	-8027.86	-7044.36	-12162.1	29.31971	0	0.834851
	34	0	8.08E-06	0.000275	0.024698	0	2.3E-10	5.53E-08	9.99E-10	3.5E-08	0			
**Total rearrangements:**	35	90131.1111	-9400.86	-7047.11	-4720.11	-17041	-14857.9	-12558.4	-14275.6	-12488.9	-21055.1	32.09214	0	0.846934
	35	0	4.65E-06	0.000147	0.014782	0	4E-11	6.17E-09	1.44E-10	7.18E-09	0			
**Total number of variants:**	36	4724247.78	-196430	-202822	-164159	-893038.22	-841090	-789075	-817815	-743688	-957660	184.3733	0	0.969502
	36	0	3.73E-05	6.04E-06	0.000449	0	0	0	0	0	0			
**Conserved Element SNPs:**	37	197587.667	-3954.92	-5520.47	-5547.67	-37051.778	-35410.4	-33948.4	-34169.2	-31612.4	-37943.5	374.4854	0	0.984748
	37	0	0.004596	3.83E-05	0.000109	0	0	0	0	0	0			
TFBS SNPs:	38	5749.55556	-248.306	-238.556	-191.806	-1175.5556	-1116.56	-1059.56	-1123.81	-1032.31	-1306.56	243.7336	0	0.976757
	38	0	1.26E-05	6.84E-06	0.000527	0	0	0	0	0	0			
TFBS SNPs/Total SNPs	39	0.007	0	0	0	0	0	0	0	0	0	0.599519	0.807727	0.093682
	39	0	1	1	1	0.10989014	1	1	1	1	1			
miRNA-BS deletion SNPs:	40	7838.22222	-113.972	-220.622	-261.472	-1547.7778	-1450.47	-1452.47	-1474.22	-1301.72	-1558.02	336.723	0	0.983067
	40	0	0.060389	0.00017	4.23E-05	0	0	0	0	0	0			
miRNA-BS deletion SNPs/Total SNPs	41	0.2348889	0.000111	0.000111	-0.00039	-0.0005556	-0.00039	0.000111	-0.00039	0.000611	0.000311	0.413101	0.934871	0.066489
	41	0	0.899692	0.891978	0.659298	0.42282442	0.659298	0.899692	0.659298	0.48889	0.703898			
miRNA-BS induction SNPs:	42	7928.22222	-135.722	-241.422	-265.222	-1609.1111	-1495.72	-1541.22	-1530.22	-1372.47	-1634.62	409.5434	0	0.986036
	42	0	0.018742	1.82E-05	1.3E-05	0	0	0	0	0	0			
miRNA-BS induction SNPs/Total SNPs	43	0.2374444	-0.00019	-0.00024	5.56E-05	-0.002	-0.00119	-0.00244	-0.00169	-0.00094	-0.00164	3.333231	0.00177	0.364956
	43	0	0.803264	0.735853	0.943243	0.00165201	0.129145	0.002482	0.032808	0.22869	0.025865			
ESE-BS deletion SNPs:	44	8687.88889	-228.639	-252.689	-245.389	-1634	-1493.89	-1443.14	-1377.14	-1270.64	-1679.89	136.452	0	0.959227
	44	0	0.019057	0.005632	0.012123	0	0	0	0	0	0			
ESE-BS deletion SNPs/Total SNPs	45	0.432	0.00175	0.0024	0.00175	0.0015556	0.002	0.00175	0.0045	0.002	0.0012	1.353914	0.224821	0.189255
	45	0	0.250841	0.091621	0.250841	0.19382227	0.190066	0.250841	0.00403	0.190066	0.395195			
ESE-BS induction SNPs:	46	8745.88889	-275.639	-338.689	-288.889	-1647	-1520.64	-1474.14	-1505.89	-1314.14	-1677.89	141.8318	0	0.960713
	46	0	0.004297	0.000215	0.002844	0	0	0	0	0	0			
ESE-BS induction SNPs/Total SNPs	47	0.4346667	-0.00067	-0.00167	-0.00042	0.0016667	0.001083	0.000833	-0.00217	8.33E-05	0.002133	1.532462	0.151273	0.208997
	47	0	0.693985	0.291008	0.805686	0.21228776	0.522983	0.622962	0.203478	0.960752	0.17766			
ESS-BS deletion SNPs:	48	4694.66667	-62.1667	-99.4667	-91.1667	-808.44444	-738.167	-719.417	-775.417	-611.667	-775.467	154.477	0	0.963813
	48	0	0.175613	0.021215	0.04874	0	0	0	0	0	0			
ESS-BS deletion SNPs/Total SNPs	49	0.4628889	0.001611	0.003111	0.002861	0.0072222	0.006611	0.004861	0.000111	0.007611	0.007911	6.273502	0.000002	0.519609
	49	0	0.416203	0.093394	0.15094	1.4687E-05	0.001302	0.01613	0.955175	0.000254	5.14E-05			
ESS-BS induction SNPs:	50	4931.22222	-101.972	-157.422	-137.722	-960.11111	-874.722	-812.722	-798.222	-758.222	-923.822	214.1015	0	0.973625
	50	0	0.02343	0.00026	0.00257	0	0	0	0	0	0			
ESS-BS induction SNPs/Total SNPs	51	0.4865556	-0.00181	-0.00276	-0.00156	-0.006	-0.00531	-0.00206	0.001444	-0.00556	-0.00516	4.073632	0.000288	0.412577
	51	0	0.371769	0.143966	0.441247	0.00030532	0.010242	0.309659	0.474423	0.007311	0.007322			
Splicing Change SNPs:	52	37.1111111	-3.86111	-3.91111	-3.11111	-9.2222222	-11.6111	-8.36111	-10.3611	-8.86111	-8.71111	6.987294	5E-07	0.546425
	52	0	0.105983	0.078259	0.191222	4.5615E-06	5.75E-06	0.000714	4.02E-05	0.000359	0.00017			
Splicing Change SNPs/Total SNPs	53	0.007	-0.00075	-0.0008	-0.0005	-0.0002222	-0.001	-0.00025	-0.0005	-0.0005	-0.0004	0.789685	0.638558	0.119837
	53	0	0.170335	0.115911	0.358793	0.60234719	0.069011	0.645572	0.358793	0.358793	0.428611			
Protein Motif disrupting SNPs:	54	786.666667	-27.1667	-30.0667	-16.4167	-146.66667	-119.167	-123.917	-126.167	-115.917	-152.667	30.62987	0	0.84079
	54	0	0.127794	0.070409	0.354788	0	4E-09	1.32E-09	7.79E-10	8.51E-09	0			
Protein Motif disrupting SNPs/Total SNPs	55	0.2204444	-0.00169	-0.00144	0.003056	0.0001111	0.003056	-0.00094	-0.00444	-0.00019	-0.00264	0.598438	0.808617	0.093529
	55	0	0.681842	0.706515	0.460387	0.9726463	0.460387	0.819182	0.284022	0.962455	0.491139			

**Conserved Element Insertions:**	56	10377.6667	-1309.67	-1000.27	-455.167	-1844.1111	-1612.17	-1312.92	-1451.92	-1272.17	-2411.47	19.91111	0	0.774417
	56	0	4.52E-06	0.000111	0.087384	0	4.97E-08	4.32E-06	5.62E-07	7.73E-06	0			
TFBS Insertions:	57	10014.8889	-1402.39	-991.289	-484.139	-1872.6667	-1665.64	-1349.64	-1516.89	-1315.39	-2463.89	24.2937	0	0.807269
	57	0	2.17E-07	4E-05	0.050253	0	2.7E-09	5.11E-07	3.29E-08	8.86E-07	0			
TFBS Insertions/Total Insertions	58	0.283	-0.00175	0.001	0.002	-0.0006667	0.00025	0.00025	0.0005	0.0015	-0.0002	1.805269	0.079869	0.237371
	58	0	0.126844	0.344669	0.081829	0.45545422	0.825888	0.825888	0.660143	0.189659	0.849618			
miRNA-BS deletion Insertions:	59	371.888889	-38.6389	-16.2889	2.361111	-55.333333	-56.3889	-42.6389	-39.1389	-33.6389	-72.4889	19.97101	0	0.774941
	59	0	4.37E-05	0.051087	0.790097	2.6E-11	1.9E-08	8.4E-06	3.57E-05	0.000307	1E-12			
miRNA-BS deletion Insertions/Total Insertions	60	0.1542222	0.006278	0.008778	0.007278	0.0058889	0.000528	0.003028	0.006028	0.003528	0.007778	1.715388	0.098925	0.22825
	60	0	0.094719	0.012952	0.053563	0.04665921	0.887113	0.416541	0.108325	0.344268	0.026916			
miRNA-BS induction Insertions:	61	297.333333	-5.58333	-4.13333	-8.83333	-52.333333	-49.3333	-49.0833	-49.8333	-41.5833	-60.7333	41.72615	0	0.877962
	61	0	0.353197	0.458487	0.143801	0	8E-12	1E-11	6E-12	1.77E-09	0			
miRNA-BS induction Insertions/Total Insertions	62	0.1233333	0.017167	0.011067	0.001167	0.0005556	-0.00158	-0.00458	-0.00408	-0.00408	0.005067	7.978005	1E-07	0.579039
	62	0	6.16E-06	0.001106	0.739705	0.8401252	0.652173	0.194446	0.247073	0.247073	0.123242			
ESE-BS deletion Insertions:	63	49.5555556	-1.30556	-2.15556	-3.05556	-8.5555556	-3.30556	-5.30556	-3.80556	-7.30556	-3.55556	5.024202	3.09E-05	0.464164
	63	0	0.531121	0.266769	0.145313	1.6307E-06	0.115643	0.012781	0.070928	0.000774	0.069134			
ESE-BS deletion Insertions/Total Insertions	64	0.2637778	0.024222	0.007422	0.001972	0.0051111	0.012222	-0.00203	0.011972	-0.01378	0.037422	4.313954	0.000162	0.426535
	64	0	0.017888	0.425702	0.843903	0.51598047	0.224869	0.839566	0.234392	0.1719	0.00014			
ESE-BS induction Insertions:	65	36.4444444	-4.44444	-4.84444	-2.44444	-7.7777778	-6.94444	-6.94444	-8.94444	-6.69444	-9.44444	6.835657	6E-07	0.540982
	65	0	0.024392	0.008665	0.209669	2.5983E-06	0.000608	0.000608	1.7E-05	0.000918	1.55E-06			
ESE-BS induction Insertions/Total Insertions	66	0.1938889	-0.00289	-0.01309	0.000361	-0.0057778	-0.01689	-0.01939	-0.02839	-0.01689	-0.01449	1.297014	0.253846	0.182755
	66	0	0.814791	0.25507	0.976636	0.55080895	0.173754	0.119188	0.023916	0.173754	0.208216			
ESS-BS deletion Insertions:	67	13.3333333	0.416667	-1.93333	1.916667	-0.2222222	1.916667	0.166667	-0.08333	-0.33333	0.066667	1.238883	0.286553	0.176006
	67	0	0.768547	0.144347	0.178651	0.84139943	0.178651	0.906272	0.953053	0.813861	0.959531			
ESS-BS deletion Insertions/Total Insertions	68	0.1555556	0.016194	-0.00336	0.033444	0.0255556	0.032194	0.011694	0.005444	0.011694	0.030444	1.301948	0.251214	0.183323
	68	0	0.383334	0.845285	0.074427	0.0820767	0.085653	0.528441	0.768891	0.528441	0.080053			
ESS-BS induction Insertions:	69	27.6666667	-0.16667	-2.06667	-3.16667	-3.1111111	-1.16667	0.083333	-0.66667	-3.16667	-3.26667	2.14012	0.03525	0.269532
	69	0	0.914069	0.152552	0.043483	0.01216032	0.450983	0.95697	0.666214	0.043483	0.025347			
ESS-BS induction Insertions/Total Insertions	70	0.3228889	0.020611	0.017111	-0.01939	0.0161111	0.000861	0.018861	0.007111	-0.00389	0.014911	0.781959	0.645642	0.118803
	70	0	0.322264	0.375641	0.351588	0.32397727	0.966893	0.364769	0.731893	0.85133	0.439763			
Splicing Change Insertions:	71	261.222222	-56.4722	-35.0222	-18.9722	-60.444444	-49.9722	-41.7222	-51.4722	-39.4722	-85.8222	8.830635	0	0.603572
	71	0	8.76E-05	0.006852	0.165231	2.9472E-07	0.000444	0.002954	0.000308	0.004778	2.96E-09			
Splicing Change Insertions/Total Insertions	72	0.4227778	-0.01028	-0.00238	-0.00478	-0.0223333	-0.01228	-0.02153	-0.02028	-0.02003	-0.02718	2.313015	0.022915	0.285099
	72	0	0.318961	0.80315	0.642191	0.00702491	0.234579	0.039216	0.051699	0.054568	0.005636			
Protein motif disrupting Insertions:	73	58.1111111	-1.86111	-0.91111	0.638889	-7.8888889	-5.36111	-3.11111	-6.11111	2.138889	-3.11111	2.481188	0.01503	0.299617
	73	0	0.574302	0.76683	0.846933	0.00327422	0.108626	0.348746	0.068204	0.518718	0.312982			
Protein motif disrupting Insertions/Total Insertions	74	0.872	0.03425	-0.003	0.0085	0.0143333	0.015	0.01125	0.01275	0.02125	0.0456	1.425478	0.192301	0.197285
	74	0	0.074315	0.864671	0.654209	0.33693394	0.429996	0.553513	0.502059	0.26467	0.011443			
**Conserved Element Deletions:**	75	11607.4444	-1338.69	-1130.44	-615.444	-2375.1111	-2174.44	-1813.44	-1998.19	-1821.44	-2957.64	31.79178	0	0.845711
	75	0	3.9E-06	2.1E-05	0.023813	0	1.2E-11	3.25E-09	1.85E-10	2.87E-09	0			
TFBS Deletions:	76	46601.7778	-8008.03	-6354.58	-3514.03	-9358.1111	-8787.78	-6857.03	-7928.78	-7139.03	-13211.6	18.93768	0	0.76554
	76	0	3.03E-07	7.25E-06	0.014973	3E-12	3.3E-08	7.05E-06	3.78E-07	3.31E-06	0			
TFBS Deletions/Total Deletions	77	0.6777778	0.002472	0.001822	0.003722	-0.0018889	0.002472	-0.00128	0.000722	0.003472	0.002822	6.061615	3.1E-06	0.511028
	77	0	0.056092	0.127357	0.004671	0.0626199	0.056092	0.318617	0.571988	0.008076	0.019621			
miRNA-BS deletion Deletions:	78	308.555556	-8.30556	-4.55556	-20.3056	-85.333333	-86.5556	-74.3056	-72.3056	-68.0556	-83.5556	67.2074	0	0.920556
	78	0	0.256821	0.501468	0.006743	0	0	0	0	0	0			
miRNA-BS deletion Deletions/Total Deletions	79	0.1195556	0.012944	0.011444	0.000194	-0.0084444	-0.01231	-0.00956	-0.00731	-0.00831	0.001044	7.989882	1E-07	0.579402
	79	0	0.005069	0.007438	0.965405	0.01870183	0.007549	0.036052	0.106607	0.067345	0.801867			
miRNA-BS induction Deletions:	80	418.777778	-31.2778	-35.1778	-34.7778	-101.77778	-95.5278	-100.278	-102.778	-82.5278	-123.378	50.19689	0	0.896423
	80	0	0.002008	0.000229	0.000656	0	0	0	0	3E-12	0			
miRNA-BS induction Deletions/Total Deletions	81	0.162	0.009	0.0028	-0.0025	-0.0042222	-0.006	-0.0125	-0.01225	-0.00625	-0.004	4.729502	6.09E-05	0.449167
	81	0	0.03318	0.468575	0.547811	0.1978519	0.151762	0.00357	0.004249	0.135676	0.301455			
ESE-BS deletion Deletions:	82	378.555556	-35.3056	-23.3556	-3.05556	-84.666667	-75.0556	-56.8056	-58.5556	-64.0556	-87.9556	34.12585	0	0.854731
	82	0	0.000259	0.007655	0.739468	0	1E-11	3.84E-08	1.77E-08	1.51E-09	0			
ESE-BS deletion Deletions/Total Deletions	83	0.4038889	0.008111	0.010111	0.016611	-0.0111111	-0.00464	-0.00164	-0.00339	-0.01114	0.008511	4.316467	0.000161	0.426677
	83	0	0.236366	0.113269	0.017036	0.04074614	0.496745	0.809966	0.619267	0.105516	0.18132			
ESE-BS induction Deletions:	84	459.777778	-58.5278	-44.5778	-35.2778	-89.666667	-82.2778	-64.2778	-66.2778	-62.0278	-120.378	15.13557	0	0.72296
	84	0	0.000151	0.001568	0.018172	4.6E-11	3.6E-07	3.81E-05	2.32E-05	6.58E-05	1E-12			
ESE-BS induction Deletions/Total Deletions	85	0.49	-0.009	-0.0074	-0.01475	0.005	0.006	0.00425	0.00225	0.00625	-0.0082	2.446871	0.016383	0.296703
	85	0	0.199582	0.255204	0.037431	0.36217465	0.39084	0.542753	0.747035	0.371514	0.207878			
ESS-BS deletion Deletions:	86	481.111111	-84.6111	-61.3111	-36.6111	-86.555556	-79.8611	-51.3611	-55.8611	-64.8611	-120.711	8.249303	0	0.587168
	86	0	5.48E-05	0.001272	0.06659	4.0068E-07	0.000127	0.01098	0.005881	0.001534	7.09E-09			
ESS-BS deletion Deletions/Total Deletions	87	0.5517778	-0.03503	-0.02298	0.000722	0.007	0.012472	0.019722	0.012972	0.000472	-0.00618	5.869649	4.7E-06	0.502984
	87	0	0.000563	0.012729	0.940687	0.35943975	0.201566	0.045347	0.184296	0.961198	0.493656			
ESS-BS induction Deletions:	88	287.888889	-10.1389	-9.88889	-18.6389	-64.222222	-59.3889	-42.6389	-41.1389	-41.3889	-67.0889	23.41715	0	0.801487
	88	0	0.234082	0.211421	0.030717	0	1.32E-09	3.63E-06	7.08E-06	6.34E-06	2E-12			
ESS-BS induction Deletions/Total Deletions	89	0.3311111	0.031139	0.021889	0.002639	-0.014	-0.01011	-0.00511	-0.00336	-0.00386	0.003289	7.663182	1E-07	0.569195
	89	0	9.97E-05	0.002556	0.726886	0.02057196	0.183522	0.499274	0.656512	0.609511	0.639208			
Splicing Change Deletions:	90	194.666667	-25.1667	-14.6667	-13.1667	-42.777778	-34.1667	-30.1667	-29.4167	-42.4167	-51.2667	10.80178	0	0.65064
	90	0	0.00304	0.057707	0.1123	6.016E-09	8.78E-05	0.000457	0.000615	2.18E-06	4.23E-09			
Splicing Change Deletions/Total Deletions	91	0.3345556	0.010444	0.011644	-0.00606	-0.0161111	-0.00531	-0.02356	-0.01481	-0.02581	-0.00016	3.14124	0.002858	0.35132
	91	0	0.330193	0.242904	0.571469	0.05800206	0.619937	0.03037	0.169004	0.018082	0.98749			
Protein motif disrupting Deletions:	92	59.1111111	0.388889	-2.31111	-4.61111	-14.555556	-11.3611	-9.61111	-14.8611	-7.36111	-16.1111	11.53148	0	0.665349
	92	0	0.895873	0.403264	0.124012	3.0152E-08	0.000277	0.001824	4.05E-06	0.015391	1.53E-07			
Protein motif disrupting Deletions/Total Deletions	93	0.6931111	0.022139	0.001089	-0.02011	0.0107778	-0.01961	-0.01586	-0.05911	-0.01661	-0.00691	1.824982	0.076179	0.239342
	93	0	0.345566	0.960004	0.391263	0.55750488	0.403083	0.498496	0.01354	0.478482	0.750357			
**Conserved Element Rearrangements:**	94	3181	-267.25	-178.4	-115.25	-528.88889	-448.25	-404.5	-437.25	-355	-684.8	29.61192	0	0.836213
	94	0	8.13E-05	0.003589	0.074766	0	1.28E-09	2.14E-08	2.62E-09	4.77E-07	0			
TFBS Rearrangements:	95	12170.6667	-1707.42	-1332.67	-727.917	-2295.6667	-2067.92	-1712.42	-1917.42	-1637.17	-3121.27	21.55651	0	0.787985
	95	0	1.07E-06	2.63E-05	0.025246	0	1.16E-08	1.01E-06	7.89E-08	2.5E-06	0			
TFBS Rearrangements/Total Rearrangements	96	0.4314444	-0.00544	-0.00624	0.001306	0.0027778	0.002556	0.002056	0.002306	0.003806	-0.00284	8.864789	0	0.604495
	96	0	0.002912	0.000296	0.461271	0.04847931	0.151583	0.247463	0.195138	0.034355	0.086561			
miRNA-BS deletion Rearrangements:	97	175.555556	-12.3056	-11.9556	-10.8056	-35.444444	-25.3056	-26.5556	-25.3056	-24.0556	-36.7556	11.79713	0	0.670401
	97	0	0.044042	0.03531	0.076011	1.65E-10	7.5E-05	3.58E-05	7.5E-05	0.000154	7.68E-09			
miRNA-BS deletion Rearrangements/Total Rearrangements	98	0.2671111	0.010889	0.003689	-0.00161	0.0056667	0.005139	-0.00111	0.004389	0.004889	0.018689	1.720412	0.097757	0.228766
	98	0	0.118774	0.566045	0.815851	0.29827444	0.458426	0.872387	0.526353	0.480494	0.004738			
miRNA-BS induction Rearrangements:	99	172.111111	-14.3611	-12.7111	-3.86111	-42.111111	-26.3611	-28.6111	-34.8611	-25.3611	-42.9111	17.11746	0	0.746918
	99	0	0.023023	0.02991	0.533761	1E-12	6.29E-05	1.7E-05	3.6E-07	0.000111	2E-10			
miRNA-BS induction Rearrangements/Total Rearrangements	100	0.262	0.00625	0.001	0.009	-0.0088889	0.00225	-0.00525	-0.01425	0.002	0.0044	1.759505	0.089096	0.232754
	100	0	0.431222	0.891832	0.258215	0.1557469	0.776463	0.508216	0.075489	0.800732	0.550121			
ESE-BS deletion Rearrangements:	101	244.666667	-26.6667	-13.6667	-15.4167	-41	-35.4167	-28.9167	-35.9167	-23.1667	-59.8667	15.04611	0	0.721771
	101	0	0.000464	0.046018	0.036965	6.2E-10	6.62E-06	0.000165	5.11E-06	0.002109	1E-12			
ESE-BS deletion Rearrangements/Total Rearrangements	102	0.4373333	-0.00158	0.006067	0.000667	0.0041111	0.001917	0.001917	-0.00183	0.009167	-0.00453	0.41483	0.93399	0.066748
	102	0	0.862361	0.474991	0.941802	0.56651468	0.833779	0.833779	0.840905	0.317278	0.593151			
ESE-BS induction Rearrangements:	103	261.777778	-27.0278	-17.1778	-15.2778	-39.666667	-36.0278	-31.5278	-36.5278	-28.7778	-54.1778	7.125177	3E-07	0.551263
	103	0	0.007062	0.061326	0.120881	2.0485E-06	0.00043	0.001851	0.000364	0.004259	8.79E-08			
ESE-BS induction Rearrangements/Total Rearrangements	104	0.4674444	0.001556	0.002356	0.002556	0.0134444	0.006806	0.000556	0.002556	0.001806	0.018556	1.087607	0.386674	0.157908
	104	0	0.872473	0.79345	0.792038	0.08039061	0.483278	0.95428	0.792038	0.852202	0.042233			
ESS-BS deletion Rearrangements:	105	174.333333	-18.3333	-12.5333	-9.33333	-21.444444	-14.3333	-13.0833	-20.5833	-9.08333	-25.9333	2.855607	0.005856	0.329914
	105	0	0.020582	0.085237	0.231497	0.00074105	0.068094	0.095175	0.009692	0.244104	0.000577			
ESS-BS deletion Rearrangements/Total Rearrangements	106	0.4504444	0.005056	0.010756	0.004056	0.0216667	0.023056	0.019306	0.017306	0.034806	0.041356	4.158036	0.000235	0.417556
	106	0	0.620893	0.258799	0.691452	0.00842946	0.026684	0.062077	0.0936	0.001067	4.28E-05			
ESS-BS induction Rearrangements:	107	189.111111	-24.1111	-16.9111	-9.86111	-37.111111	-32.1111	-25.8611	-30.6111	-33.1111	-50.9111	15.62682	0	0.729311
	107	0	0.000246	0.004673	0.117877	1.27E-10	2.4E-06	9.44E-05	5.97E-06	1.29E-06	1E-12			
ESS-BS induction Rearrangements/Total Rearrangements	108	0.4893333	-0.00733	0.001867	0.004917	-0.02	-0.02483	-0.01358	-0.00658	-0.03033	-0.03013	3.099768	0.003171	0.348298
	108	0	0.515968	0.858409	0.662917	0.02640756	0.030423	0.230683	0.559679	0.008742	0.005172			
Splicing Change Rearrangements:	109	30.2222222	-2.72222	-3.82222	1.527778	-4.6666667	-1.72222	2.027778	-3.47222	-2.97222	-6.22222	2.448933	0.016298	0.296879
	109	0	0.296942	0.116506	0.557201	0.02473939	0.508303	0.436363	0.184507	0.255157	0.011807			
Splicing Change Rearrangements/Total Rearrangements	110	0.2124444	0.009556	-0.01084	0.016806	0.0038889	0.016306	0.026306	-0.00369	0.010056	0.006556	0.894674	0.543715	0.13364
	110	0	0.561765	0.478333	0.308787	0.76318947	0.323293	0.113132	0.822313	0.54153	0.66785			
Protein motif disrupting Rearrangements:	111	88.5555556	-19.5556	-9.35556	-7.30556	-10.444444	-7.05556	-8.55556	-15.5556	-4.55556	-19.7556	4.17466	0.000226	0.418527
	111	0	0.000184	0.045191	0.143735	0.00886453	0.15773	0.087792	0.00244	0.359588	5.48E-05			
Protein motif disrupting Rearrangements/Total Rearrangements	112	0.612	-0.008	0	0.03175	0.0087778	0.033	0.02825	-0.0095	0.01175	0.0078	1.26507	0.271429	0.17906
	112	0	0.681473	1	0.106506	0.56613676	0.09367	0.150131	0.626055	0.546899	0.666378			

Nonsense SNPs:	113	107.111111	3.638889	1.888889	-6.61111	-16.666667	-14.1111	-16.1111	-16.6111	-16.3611	-9.71111	6.643385	9E-07	0.533889
	113	0	0.464981	0.682404	0.186358	5.8622E-05	0.005808	0.001787	0.001312	0.001532	0.03834			
Nonsense SNPs/Total SNPs	114	0.0041111	0.000139	8.89E-05	-0.00011	0.0002222	-0.00011	0.000139	0.000139	0.000139	0.000489	0.986566	0.4653	0.14537
	114	0	0.595597	0.714248	0.671015	0.28062349	0.671015	0.595597	0.595597	0.595597	0.047129			
Frameshift Structural Variants:	115	414.333333	-30.0833	-17.9333	-25.0833	-78.444444	-46.8333	-37.8333	-41.8333	-33.3333	-81.7333	14.00599	0	0.707159
	115	0	0.014699	0.112417	0.040573	6E-12	0.000226	0.002441	0.000879	0.007144	3.86E-10			
Frameshift Structural Variants/Total Variants	116	0.4642222	0.022528	0.014578	0.005528	-0.0094444	0.001278	0.001278	0.001278	-0.00147	0.012578	4.07931	0.000284	0.412915
	116	0	0.002668	0.033156	0.446629	0.10011111	0.860072	0.860072	0.860072	0.839056	0.064916			
Splicing Change Variants:	117	523.222222	-88.2222	-57.4222	-33.7222	-117.11111	-97.4722	-78.2222	-94.7222	-93.7222	-152.022	12.93956	0	0.690494
	117	0	5.95E-05	0.003726	0.106018	5.32E-10	1.17E-05	0.000314	1.92E-05	2.29E-05	2.9E-11			
Splicing Change Variants/Total Variants	118	0.0786667	-0.00842	-0.00507	-0.00217	-0.0026667	8.33E-05	0.001583	-0.00042	-0.00242	-0.00767	2.94834	0.004637	0.337017
	118	0	0.00409	0.058047	0.446709	0.23402213	0.976602	0.577791	0.883419	0.396292	0.004792			
Probably Damaging nscSNPs:	119	1858.11111	-21.1111	-47.7111	-77.6111	-359.66667	-304.861	-301.111	-304.361	-308.611	-364.311	114.5781	0	0.951819
	119	0	0.359599	0.028003	0.001171	0	0	0	0	0	0			
Probably Damaging nscSNPs/Total nscSNPs	120	0.155	0.002	0.0014	-0.00225	-0.0008889	0.00075	0.00025	-0.001	-0.00375	-0.0002	1.741802	0.092926	0.230953
	120	0	0.263506	0.398135	0.208987	0.52509389	0.673756	0.888331	0.574769	0.038217	0.903671			
Possibly Damaging nscSNPs:	121	1297.77778	-10.5278	-36.1778	-53.5278	-276.88889	-240.778	-218.028	-195.028	-217.778	-257.178	50.37234	0	0.896746
	121	0	0.682307	0.13271	0.040394	0	0	3E-12	1.19E-10	3E-12	0			
Possibly Damaging nscSNPs/Total nscSNPs	122	0.1082222	0.002028	0.000578	-0.00122	-0.0034444	-0.00222	-0.00072	0.001528	-0.00297	-0.00022	2.788659	0.006931	0.324691
	122	0	0.236482	0.715005	0.474034	0.01187161	0.194981	0.671872	0.371349	0.084542	0.888265			
Protein motif damaging Variants:	123	992.444444	-48.1944	-42.6444	-27.6944	-179.55556	-142.944	-145.194	-162.694	-125.694	-191.644	31.70409	0	0.84535
	123	0	0.022398	0.029205	0.183694	0	1.99E-09	1.27E-09	3.8E-11	6.01E-08	0			
Protein motif damaging Variants/Total Variants	124	0.2566667	-0.00417	-0.00227	0.002583	0.0014444	0.005333	0.001833	-0.00592	0.004333	-0.00187	1.745375	0.092141	0.231317
	124	0	0.273782	0.520027	0.49625	0.62740526	0.162458	0.628895	0.121852	0.255223	0.596082			
TFBS Disrupting Variants:	125	76310.6667	-11766.4	-9252.07	-5067.67	-15039.556	-13963.2	-11179.9	-12741.9	-11364.9	-20662.5	22.20582	0	0.7929
	125	0	2.95E-07	8.38E-06	0.016736	0	4.04E-09	8.98E-07	4.48E-08	6.33E-07	0			
TFBS Disrupting Variants/Total Variants	126	0.0785556	-0.00856	-0.00616	-0.00256	-0.0003333	0.000194	0.002444	0.000944	0.001194	-0.00616	10.63067	0	0.647002
	126	0	2.04E-06	0.000144	0.124976	0.79694334	0.906252	0.14194	0.567769	0.470265	0.000144			
miRNA-BS Disrupting Variants:	127	17510.6667	-360.167	-546.867	-602.917	-3529.2222	-3285.67	-3315.17	-3328.67	-2949.42	-3612.47	374.2751	0	0.98474
	127	0	0.006441	1.92E-05	1.3E-05	0	0	0	0	0	0			
miRNA-BS Disrupting Variants/Total Variants	128	0.4486667	0.005083	0.003133	0.000833	-0.0025556	-0.00292	-0.00442	-0.00367	-0.00192	0.001333	10.14119	0	0.636163
	128	0	0.000464	0.017115	0.548146	0.02121455	0.038364	0.002107	0.009877	0.169649	0.301857			
ESE-BS Disrupting Variants:	129	18864.5556	-657.556	-697.156	-608.806	-3552.3333	-3253.56	-3111.06	-3093.06	-2776.81	-3693.16	127.0182	0	0.956331
	129	0	0.002716	0.000695	0.005289	0	0	0	0	0	0			
ESE-BS Disrupting Variants/Total Variants	130	0.8653333	0.000917	0.001267	0.001417	0.0033333	0.002667	0.002417	0.001667	0.001417	0.003667	3.821372	0.000531	0.397175
	130	0	0.366452	0.180383	0.164558	7.6452E-05	0.010161	0.019314	0.102954	0.164558	0.000212			
ESS-BS Disrupting Variants:	131	10799.3333	-301.083	-361.533	-304.583	-1981.2222	-1797.83	-1664.83	-1722.58	-1521.83	-1967.13	140.2698	0	0.960293
	131	0	0.008691	0.00084	0.007979	0	0	0	0	0	0			
ESS-BS Disrupting Variants/Total Variants	132	0.9404444	0.000806	0.001556	0.002556	0.001	0.001056	0.003806	0.002306	0.002056	0.003156	4.938592	3.76E-05	0.459892
	132	0	0.334574	0.047166	0.002971	0.12874414	0.207212	1.99E-05	0.007015	0.015653	0.000113			
**Total Likely Functional Variants:**	133	112980.556	-12437.1	-10211.4	-6119.06	-22095	-20389.1	-17606.1	-19178.6	-16993.8	-27730.2	37.24391	0	0.865254
	133	0	9.57E-07	1.02E-05	0.009887	0	1E-12	1.07E-10	6E-12	3.22E-10	0			
synonymous to nonsynonymous rate	134	1.1405246	-0.00647	0.000375	-0.00143	-0.0044485	-0.01254	-0.01176	-0.0199	-0.00367	0.000736	1.817884	0.077489	0.238634
	134	0	0.373768	0.95552	0.843318	0.43500932	0.08714	0.108147	0.007541	0.613376	0.912884			
Likely functional variant rate	135	0.0239041	-0.0017	-0.00119	-0.00047	-0.0001882	-6.1E-05	0.000332	0.000107	0.00021	-0.00128	9.731146	0	0.626557
	135	0	1.97E-06	0.000198	0.154487	0.46441494	0.852449	0.312119	0.743856	0.522668	7.14E-05			
Likely functional variant rate (minus probdam)	136	0.0236294	-0.00171	-0.0012	-0.00047	-0.00018	-5.8E-05	0.000332	9.94E-05	0.000213	-0.00128	9.911546	0	0.630845
	136	0	1.59E-06	0.000176	0.153993	0.48172171	0.857691	0.309265	0.760314	0.514006	6.5E-05			
Functional Nonsense SNPs/Total Variants	137	0.0000227	1.8E-06	1.4E-06	-6E-07	0.000001	1.3E-06	5E-07	5E-07	1E-07	3.2E-06	2.106697	0.038291	0.266445
	137	0	0.110786	0.173438	0.569416	0.27419535	0.250094	0.679536	0.654859	0.919041	0.002833			
Functional Frameshift Structural Variants/Total Variants	138	0.0000877	-2.8E-06	-1E-07	-2.3E-06	0	0.000007	0.000008	7.7E-06	0.000008	6E-07	7.647868	1E-07	0.568705
	138	0	0.219206	0.97779	0.313689	0.98722149	0.003071	0.000759	0.001188	0.000725	0.78714			
Functional Splicing Change Variants/Total Variants	139	0.0001107	-1.5E-05	-7.9E-06	-3.4E-06	-0.0000048	-1.1E-06	2.4E-06	-1E-06	-2.8E-06	-1.2E-05	3.49987	0.00117	0.376335
	139	0	0.000941	0.048956	0.427081	0.15050799	0.796187	0.575719	0.809221	0.510363	0.00255			
Probably Damaging nscSNPs/Total Variants	140	0.0003934	1.23E-05	0.000007	-2.9E-06	-0.0000021	6.6E-06	2.3E-06	4.3E-06	-4.1E-06	3.2E-06	2.35318	0.020724	0.288621
	140	0	0.013018	0.124409	0.549509	0.57545483	0.175033	0.640138	0.371083	0.396643	0.476787			
Possibly Damaging nscSNPs/Total Variants	141	0.0002747	9.6E-06	4.3E-06	-1.8E-06	-0.0000082	-2.5E-06	-3E-07	7.6E-06	-3.3E-06	1.6E-06	2.566531	0.012126	0.306762
	141	0	0.067903	0.377297	0.731407	0.04916263	0.633978	0.95371	0.147346	0.524012	0.747569			
Functional Protein motif damaging Variants/Total Variants	142	0.00021	-1.4E-06	1E-07	1.6E-06	0.0000022	8.8E-06	5.3E-06	2.4E-06	7.7E-06	2.6E-06	1.466041	0.175725	0.201766
	142	0	0.714144	0.982305	0.68	0.46608859	0.027746	0.178137	0.538431	0.053366	0.473256			
Functional TFBS Disrupting Variants/Total Variants	143	0.0161418	-0.00189	-0.00133	-0.00052	-0.0001577	-8.7E-05	0.000409	0.00013	0.000173	-0.00138	10.68495	0	0.648164
	143	0	6.89E-07	9.34E-05	0.136755	0.56112927	0.800808	0.238861	0.707024	0.61675	5.33E-05			
Functional miRNA-BS Disrupting Variants/Total Variants	144	0.0037069	0.000081	4.54E-05	1.1E-06	-0.0000566	-4.4E-05	-1E-04	-7.6E-05	-4.9E-05	-1.6E-05	11.52404	0	0.665205
	144	0	0.00081	0.037783	0.963697	0.00261718	0.063577	5.36E-05	0.001519	0.039287	0.449563			
Functional ESE-BS Disrupting Variants/Total Variants	145	0.0039926	2.85E-05	2.49E-05	1.09E-05	0.000005	2.75E-05	1.04E-05	4.45E-05	4.92E-05	3.53E-05	0.940923	0.503538	0.139584
	145	0	0.297302	0.326224	0.689238	0.81407588	0.314415	0.701948	0.106064	0.074745	0.166448			
Functional ESS-BS Disrupting Variants/Total Variants	146	0.0022857	3.29E-05	2.28E-05	1.58E-05	0.0000164	3.24E-05	3.55E-05	3.77E-05	4.52E-05	5.93E-05	4.267048	0.000181	0.423863
	146	0	0.017532	0.073601	0.245898	0.12779488	0.019591	0.010706	0.006861	0.001371	1.24E-05			
