# Supplementary material for: Clinical Implications of Human Population Differences in Genome-Wide Rates of Functional Genotypes
Source: Front Genet. 2012 Nov 1;3:211. doi: 10.3389/fgene.2012.00211 (PMC3485509; doi:10.3389/fgene.2012.00211)
Supplement: Supplementary Data Sheet S2 — Regression analysis results for reference-based variants: novel variants. [file 32001_Schork_DataSheet2.PDF]

Reference-Based Variants: Novel Variants
Row 1: Regression Coefficients
Row 2: P-values for Regression Coefficients

	Var Cat	Y-int	LWK	ASW	MKK	CEU	TSI	CHB	JPT	GIH	MEX	F-Stat	P-Value	R-Sqr
Coding SNPs:	1	470.111111	355.6389	4.888889	386.8889	-258.77778	-90.1111	45.88889	-128.111	354.1389	-26.3111	18.72026	0	0.763461
	1	0	2.28E-05	0.946417	5.19E-06	7.3847E-05	0.252558	0.558693	0.105527	2.44E-05	0.717709			
Nonsynonymous SNPs:	2	278.111111	206.3889	-5.71111	219.3889	-143.77778	-29.3611	46.63889	-69.1111	218.3889	0.688889	16.41056	0	0.738863
	2	0	5.22E-05	0.89783	1.96E-05	0.00027609	0.540605	0.332093	0.152358	2.12E-05	0.987642			
Synonymous SNPs:	3	185.888889	146.3611	10.51111	165.8611	-112.66667	-59.8889	-1.63889	-57.8889	132.6111	-29.0889	21.12513	0	0.784588
	3	0	1.38E-05	0.717699	1.3E-06	1.8995E-05	0.059106	0.958252	0.067858	6.73E-05	0.318626			
Nonsense SNPs:	4	6.1111111	2.888889	0.088889	1.638889	-2.3333333	-0.86111	0.888889	-1.11111	3.138889	2.088889	2.22405	0.028614	0.277173
	4	1.8972E-07	0.132382	0.959876	0.390563	0.12149068	0.651246	0.640793	0.559914	0.10257	0.239537			
Untranslated Region SNPs:	5	645.555556	378.1944	-23.9556	531.6944	-384.22222	-221.306	-90.5556	-233.556	323.6944	-129.956	19.37838	0	0.769644
	5	0	0.000366	0.798571	1.52E-06	7.3408E-06	0.031485	0.371885	0.023477	0.002018	0.169154			
Non-coding RNA SNPs:	6	17186.6667	10439.08	-1925.87	11831.58	-9729.1111	-5564.92	-2474.67	-6171.67	6668.333	-4382.07	24.04697	0	0.805675
	6	0	1.3E-05	0.352803	1.22E-06	4.4586E-07	0.014513	0.268423	0.006988	0.003711	0.036933			
Intronic SNPs:	7	24735.1111	15978.14	-2530.71	18397.39	-14186.111	-8341.61	-3416.61	-9145.11	10730.14	-5733.31	23.66966	0	0.803187
	7	0	1.17E-05	0.421864	7.7E-07	1.1046E-06	0.015962	0.314845	0.008521	0.002228	0.071568			
Intergenic SNPs:	8	33915.8889	20765.36	-3313.69	23724.36	-19309.778	-11323.9	-5239.14	-12301.9	12645.11	-8607.09	22.6238	0	0.795946
	8	0	2.12E-05	0.434325	1.85E-06	8.6519E-07	0.015073	0.252491	0.008529	0.006935	0.044991			
**Total SNPs:**	9	59890.3333	37558.92	-5877.93	43110.92	-34206.444	-20017.6	-8716.58	-21854.8	24111.42	-14535.3	23.0874	0	0.79922
	9	0	1.64E-05	0.436223	1.23E-06	9.7102E-07	0.015822	0.284837	0.008695	0.003987	0.056971			
Coding Insertions:	10	30.3333333	-2.58333	-2.33333	0.916667	-13.222222	-2.58333	-0.83333	-7.08333	-1.08333	-3.53333	4.410114	0.000129	0.431936
	10	0	0.475953	0.487856	0.79999	1.4567E-05	0.475953	0.81783	0.053494	0.764637	0.294611			
In-frame Insertions:	11	3.2222222	-1.72222	-0.22222	0.527778	-1.8888889	-1.97222	-0.97222	-1.22222	-0.72222	-2.42222	2.054042	0.043605	0.261527
	11	4.9213E-07	0.103481	0.819202	0.614616	0.02410202	0.063042	0.354763	0.245563	0.491187	0.014827			
Out-of-frame Insertions:	12	7.8888889	-4.38889	-2.88889	-1.63889	-5.1111111	-3.38889	-2.63889	-4.88889	-1.63889	-4.88889	4.865518	4.44E-05	0.456191
	12	0	0.001748	0.023833	0.227623	7.9526E-06	0.014203	0.05408	0.000545	0.227623	0.000215			
Frameshift Insertions:	13	19.2222222	3.527778	0.777778	2.027778	-6.2222222	2.777778	2.777778	-0.97222	1.277778	3.777778	2.559351	0.012347	0.306166
	13	0	0.288558	0.800194	0.540696	0.01892857	0.402592	0.402592	0.769043	0.69963	0.221434			
Untranslated region Insertions:	14	177.222222	0.777778	-1.82222	9.027778	-89.333333	-56.2222	-47.9722	-52.7222	-33.7222	-59.2222	17.27819	0	0.748681
	14	0	0.954044	0.884353	0.504261	3E-12	8.6E-05	0.000671	0.00021	0.01457	1.13E-05			
Non-coding RNA Insertions:	15	4663.77778	-281.528	-318.378	-300.278	-2224.2222	-1522.28	-1496.28	-1627.78	-1192.03	-1785.58	44.99727	0	0.885821
	15	0	0.159726	0.087824	0.134102	0	8.8E-11	1.52E-10	1E-11	8.19E-08	0			
Intronic Insertions:	16	7345.77778	-445.528	-474.978	-404.278	-3470.5556	-2376.28	-2271.53	-2487.28	-1932.28	-2826.38	43.48162	0	0.882309
	16	0	0.162778	0.109713	0.204733	0	1.71E-10	6.76E-10	4E-11	5.46E-08	0			
Intergenic Insertions:	17	9106.11111	-480.861	-550.311	-501.611	-4411.5556	-3004.86	-2952.61	-3176.86	-2463.36	-3503.11	53.43262	0	0.902081
	17	0	0.19051	0.107677	0.172318	0	8E-12	1.5E-11	1E-12	3.83E-09	0			
**Total Insertions:**	18	16681.3333	-923.583	-1031.73	-894.583	-7995.6667	-5447.33	-5279.83	-5733.08	-4435.33	-6399.33	48.65689	0	0.893494
	18	0	0.184726	0.111469	0.198717	0	3.6E-11	9.8E-11	6E-12	1.53E-08	0			
Coding Deletions:	19	29.2222222	1.527778	2.377778	5.027778	-13	-5.22222	-4.47222	-5.47222	2.027778	-5.22222	5.320852	1.58E-05	0.478457
	19	0	0.698352	0.516233	0.204657	7.4662E-05	0.187896	0.258621	0.167896	0.607142	0.156414			
In-frame Deletions:	20	3	1.75	1.2	-1.25	-1.5555556	-0.5	-0.75	-0.25	-0.25	-0.8	2.20457	0.030036	0.275414
	20	2.536E-06	0.100694	0.223136	0.238681	0.06362979	0.635938	0.478118	0.812779	0.812779	0.415263			
Inter-Codon Deletions:	21	6.4444444	-1.19444	-0.04444	4.055556	-3.6666667	-2.94444	-0.19444	0.055556	0.055556	-2.44444	3.729295	0.000665	0.391351
	21	3.042E-09	0.484956	0.977625	0.019946	0.00769574	0.088032	0.909326	0.974041	0.974041	0.126249			
Frameshift Deletions:	22	19.7777778	0.972222	1.222222	2.222222	-7.7777778	-1.77778	-3.52778	-5.27778	2.222222	-1.97778	3.568801	0.000987	0.380924
	22	0	0.741607	0.655287	0.451832	0.00122569	0.54694	0.233827	0.076784	0.451832	0.470583			
Untranslated region Deletions:	23	204.777778	36.47222	1.222222	4.472222	-119.77778	-91.0278	-90.7778	-86.0278	-74.5278	-91.1778	48.63483	0	0.893451
	23	0	0.004531	0.915853	0.719823	0	3.85E-10	4.18E-10	2.03E-09	8.77E-08	3.5E-11			
Non-coding RNA Deletions:	24	5550.66667	487.8333	-276.267	2.333333	-3124	-2471.17	-2424.42	-2559.42	-2007.42	-2467.87	75.70588	0	0.92884
	24	0	0.053727	0.235118	0.992534	0	0	0	0	1.7E-11	0			
Intronic Deletions:	25	8623.77778	714.7222	-383.378	-193.278	-4873.3333	-3897.03	-3810.53	-3940.03	-3051.53	-3863.58	68.25432	0	0.921679
	25	0	0.081936	0.311104	0.634494	0	0	0	0	1.62E-10	0			
Intergenic Deletions:	26	10916.1111	872.8889	-443.911	-24.3611	-6090.6667	-4866.36	-4776.11	-5052.36	-3906.61	-4850.11	72.2577	0	0.925696
	26	0	0.083891	0.339823	0.961089	0	0	0	0	4.4E-11	0			
**Total Deletions:**	27	19797	1632.5	-818.2	-208.25	-11111.444	-8873	-8688.75	-9097.25	-7039	-8815	70.90721	0	0.924388
	27	0	0.077843	0.337034	0.819997	0	0	0	0	7.6E-11	0			
Coding rearrangements:	28	25.8888889	-1.88889	5.111111	-2.38889	-12.555556	-1.63889	-4.38889	-4.63889	-5.13889	-9.68889	6.388714	1.6E-06	0.52415
	28	0	0.574547	0.104742	0.478037	9.965E-06	0.626108	0.194407	0.170511	0.129548	0.002687			
In-frame rearrangements:	29	21.6666667	-1.16667	2.933333	-3.16667	-11.111111	-5.16667	-4.41667	-2.91667	-4.16667	-8.26667	5.401923	1.32E-05	0.482232
	29	0	0.698776	0.296241	0.295272	1.2524E-05	0.089846	0.145899	0.334747	0.169738	0.004168			
Frameshift rearrangements:	30	4.2222222	-0.72222	2.177778	0.777778	-1.4444444	3.527778	0.027778	-1.72222	-0.97222	-1.42222	4.735437	6.01E-05	0.449477
	30	4.849E-09	0.52609	0.042269	0.494866	0.1088902	0.002722	0.980517	0.133277	0.393991	0.180883			
Untranslated region rearrangements:	31	58.7777778	-2.27778	-2.57778	-5.02778	-31.888889	-27.5278	-19.2778	-23.2778	-17.2778	-31.5778	22.41662	0	0.794447
	31	0	0.606694	0.530416	0.25764	0	3.23E-08	4.31E-05	1.47E-06	0.000208	7.5E-11			
Non-coding RNA rearrangements:	32	2034	-65.75	-133.4	-175.5	-1108.3333	-860	-710.25	-842.25	-698.5	-1024.2	33.90689	0	0.85393
	32	0	0.588197	0.238531	0.151102	0	9.37E-10	1.45E-07	1.72E-09	2.13E-07	0			
Intronic rearrangements:	33	2793.55556	-19.0556	-264.356	-252.306	-1605.2222	-1240.31	-1124.56	-1200.81	-1042.06	-1433.56	37.68225	0	0.866612
	33	0	0.908764	0.090181	0.132446	0	2.02E-10	3.61E-09	5.42E-10	2.75E-08	0			
Intergenic rearrangements:	34	4005.66667	-153.667	-283.267	-354.167	-2248.2222	-1736.67	-1518.42	-1658.42	-1424.42	-2035.47	34.85307	0	0.857329
	34	0	0.523809	0.207517	0.14436	0	5.57E-10	2.32E-08	2.14E-09	1.13E-07	0			
**Total rearrangements:**	35	6896.55556	-174.806	-545.556	-616.556	-3905.2222	-3009.31	-2669.56	-2892.31	-2493.06	-3517.36	36.44859	0	0.862717
	35	0	0.669791	0.154474	0.13556	0	3.24E-10	9.95E-09	1.06E-09	5.74E-08	0			
**Total number of variants:**	36	103265.222	38093.03	-8273.42	41391.53	-57218.778	-37347.2	-25354.7	-39577.5	10144.03	-33267	27.85058	0	0.82764
	36	0	0.000176	0.355824	5.33E-05	1.22E-10	0.000229	0.010173	0.000103	0.293793	0.000385			
**Conserved Element SNPs:**	37	3232.88889	2391.861	-294.889	2848.111	-1722.5556	-802.889	-139.639	-1055.64	2086.111	-387.689	22.13537	0	0.792378
	37	0	7.48E-06	0.521013	2.1E-07	3.2154E-05	0.107692	0.777611	0.035685	7.09E-05	0.399338			
TFBS SNPs:	38	136.444444	63.80556	10.15556	86.55556	-81.666667	-45.6944	-25.9444	-55.9444	35.05556	-46.2444	21.34991	0	0.786371
	38	0	0.000672	0.542795	8E-06	1.8152E-07	0.012905	0.151575	0.002606	0.054165	0.006943			
TFBS SNPs/Total SNPs	39	0.0093333	-0.00083	0.001067	0.000167	-0.0004444	0.000417	-8.3E-05	-0.00058	-8.3E-05	-0.00073	2.178917	0.032014	0.273084
	39	0	0.151057	0.049236	0.772346	0.3269782	0.470245	0.884957	0.312963	0.884957	0.173098			
miRNA-BS deletion SNPs:	40	110.111111	81.38889	0.488889	117.1389	-62.888889	-23.8611	-5.36111	-39.3611	87.38889	-12.5111	18.89754	0	0.765159
	40	0	0.000153	0.979351	2.15E-07	0.00018765	0.243384	0.792257	0.056408	5.47E-05	0.50843			
miRNA-BS deletion SNPs/Total SNPs	41	0.2134444	0.009556	0.009156	0.007056	-0.0085556	0.027306	0.008056	-0.00619	0.027306	0.012356	1.858383	0.07029	0.24266
	41	0	0.481083	0.467099	0.602597	0.42156985	0.046894	0.552323	0.647506	0.046894	0.327192			
miRNA-BS induction SNPs:	42	125.222222	80.77778	-3.22222	133.2778	-74.555556	-35.7222	-6.97222	-40.4722	81.27778	-18.2222	18.73418	0	0.763595
	42	0	0.000567	0.876841	9.9E-08	6.5519E-05	0.114121	0.755671	0.074205	0.000528	0.382129			
miRNA-BS induction SNPs/Total SNPs	43	0.2396667	0.000833	0.005333	0.013833	-0.013	0.012583	0.011083	0.005833	0.011833	0.007133	1.841445	0.07322	0.240981
	43	0	0.936065	0.580618	0.185874	0.11403813	0.228319	0.288061	0.57489	0.256959	0.460345			
ESE-BS deletion SNPs:	44	143.555556	125.4444	7.244444	121.1944	-78.333333	-24.0556	13.94444	-33.3056	105.1944	1.444444	18.97863	0	0.765927
	44	0	2.16E-06	0.747963	4.21E-06	0.00010349	0.323581	0.566236	0.173144	4.77E-05	0.948897			
ESE-BS deletion SNPs/Total SNPs	45	0.3918889	0.023611	0.017311	0.004111	0.0073333	0.006111	0.001361	0.026361	-0.00014	0.016111	0.739997	0.684097	0.113149
	45	0	0.160413	0.266206	0.805551	0.57600351	0.714494	0.93503	0.117734	0.993363	0.300478			
ESE-BS induction SNPs:	46	169.222222	119.7778	1.577778	144.7778	-91.444444	-30.2222	22.27778	-43.2222	131.2778	-3.42222	16.54875	0	0.740478
	46	0	0.000144	0.95454	7.04E-06	0.00020788	0.312651	0.455931	0.150353	3.71E-05	0.901599			
ESE-BS induction SNPs/Total SNPs	47	0.463	-0.01725	-0.002	0.00325	0.0127778	-0.003	0.01525	0.00575	0.00975	0.0038	0.702701	0.718009	0.108063
	47	0	0.28809	0.893994	0.840724	0.31558699	0.852827	0.347208	0.722257	0.547058	0.800165			
ESS-BS deletion SNPs:	48	68.1111111	56.63889	-4.11111	57.88889	-37.555556	-10.6111	4.138889	-15.1111	65.88889	-1.51111	15.26069	0	0.724606
	48	0	8.92E-05	0.745312	6.46E-05	0.00076753	0.437309	0.761468	0.269769	7.62E-06	0.904929			
ESS-BS deletion SNPs/Total SNPs	49	0.3874444	0.006306	-0.00784	-0.02244	0.0122222	0.009306	-0.01069	0.015806	0.027056	-0.00344	0.484521	0.893369	0.077098
	49	0	0.828366	0.771409	0.44124	0.59251132	0.749062	0.713191	0.587185	0.353727	0.898463			
ESS-BS induction SNPs:	50	98.4444444	78.55556	-1.64444	88.30556	-52	-15.6944	12.55556	-23.6944	74.80556	2.155556	16.99883	0	0.745601
	50	0	2.7E-05	0.919323	3.39E-06	0.00031053	0.371001	0.473712	0.17847	5.82E-05	0.89438			
ESS-BS induction SNPs/Total SNPs	51	0.5574444	0.000806	0.017556	0.017556	-0.0065556	-0.00269	0.016056	0.002806	-0.02094	0.021756	0.41678	0.93299	0.067041
	51	0	0.978862	0.534467	0.564136	0.783479	0.929382	0.597818	0.926479	0.491658	0.441761			
Splicing Change SNPs:	52	0.6666667	1.333333	0.333333	1.083333	0.1111111	0.583333	0.833333	0.083333	1.333333	0.933333	1.18492	0.319766	0.16964
	52	0.10443978	0.072289	0.624435	0.142573	0.84677285	0.427157	0.257799	0.909474	0.072289	0.173043			
Splicing Change SNPs/Total SNPs	53	0.0077778	0.005472	0.003422	0.006472	0.0115556	0.009472	0.009972	0.004472	0.005472	0.012622	0.528908	0.862718	0.08357
	53	0.14334438	0.565257	0.69823	0.496631	0.12449573	0.320738	0.296051	0.638241	0.565257	0.155611			
Protein Motif disrupting SNPs:	54	30	23.25	-5	24.75	-14.444444	2.75	9.5	0	23.75	4.4	15.17031	0	0.723419
	54	0	4.25E-05	0.313716	1.53E-05	0.00091574	0.606021	0.077945	1	3.03E-05	0.374907			
Protein Motif disrupting SNPs/Total SNPs	55	0.3046667	-0.00192	-0.03307	0.020333	0.0095556	0.036083	0.013083	0.067083	-0.00817	0.031533	1.408115	0.199798	0.195351
	55	0	0.953686	0.282448	0.538376	0.71218488	0.276354	0.691942	0.045257	0.80459	0.305177			
**Conserved Element Insertions:**	56	656.555556	5.694444	-24.7556	7.444444	-299.66667	-156.556	-171.806	-173.056	-131.806	-205.956	23.63053	0	0.802926

	56	0	0.879033	0.476753	0.842298	0	8.05E-05	1.87E-05	1.65E-05	0.000742	1.07E-07			
TFBS Insertions:	57	736.888889	-51.8889	-51.8889	-66.8889	-363.88889	-261.139	-258.639	-270.639	-191.889	-320.089	46.41432	0	0.888919
	57	0	0.113927	0.089026	0.042823	0	1.9E-11	2.6E-11	6E-12	1.21E-07	0			
TFBS Insertions/Total Insertions	58	0.2911111	-0.00386	0.001689	0.002639	0.0206667	0.012889	0.009139	0.009639	0.021889	0.006289	3.523039	0.001105	0.377885
	58	0	0.599706	0.804504	0.719678	0.00060862	0.082921	0.216323	0.192516	0.003905	0.358104			
miRNA-BS deletion Insertions:	59	18.2222222	1.027778	2.577778	3.277778	-9.6666667	-3.97222	-6.97222	-5.72222	-4.97222	-5.62222	9.695257	0	0.625692
	59	0	0.652033	0.225241	0.153228	8.3934E-07	0.08458	0.003064	0.014054	0.031904	0.00952			
miRNA-BS deletion Insertions/Total Insertions	60	0.1193333	0.000167	0.017067	0.015417	-0.0036667	0.014417	-0.02008	-0.00533	-0.01158	-0.00113	0.796431	0.632377	0.120737
	60	0	0.992939	0.330653	0.414211	0.80404475	0.444997	0.288317	0.777108	0.539116	0.94831			
miRNA-BS induction Insertions:	61	8.6666667	2.333333	-0.06667	2.083333	-5.2222222	-2.41667	-2.66667	-2.66667	-1.16667	-2.66667	4.078505	0.000285	0.412867
	61	6E-12	0.217131	0.969521	0.269927	0.00069947	0.201339	0.159112	0.159112	0.535424	0.12972			
miRNA-BS induction Insertions/Total Insertions	62	0.056	0.012	0.0008	0.00925	-0.0097778	0.00225	-0.005	-0.00225	0.0035	0.0002	0.66697	0.749954	0.103135
	62	6E-12	0.326494	0.943614	0.448781	0.30826351	0.853528	0.681765	0.853528	0.774011	0.985892			
ESE-BS deletion Insertions:	63	4	1	0.4	1.25	-1.6666667	1	-0.5	-0.5	0	1.4	1.691934	0.104552	0.225834
	63	1.6597E-07	0.42065	0.728045	0.314777	0.08977321	0.42065	0.686666	0.686666	1	0.225933			
ESE-BS deletion Insertions/Total Insertions	64	0.2082222	0.084028	0.017978	0.021528	-0.008	0.052028	0.004778	0.032028	-0.00172	0.140778	1.396389	0.205002	0.19404
	64	4.4455E-08	0.171688	0.751127	0.72448	0.86734276	0.395355	0.93762	0.600209	0.977494	0.015115			
ESE-BS induction Insertions:	65	3.2222222	0.027778	-0.02222	1.027778	-1.5555556	-1.47222	-0.47222	-1.22222	-0.22222	-0.62222	1.703288	0.101792	0.227006
	65	4.1984E-08	0.976489	0.979735	0.277646	0.03844291	0.121633	0.616692	0.197514	0.813648	0.477773			
ESE-BS induction Insertions/Total Insertions	66	0.1657778	0.039722	-0.00238	0.020222	0.0388889	-0.07578	-0.00328	-0.03153	-0.01528	0.037222	0.516525	0.871608	0.081774
	66	0.00011541	0.587925	0.972094	0.782491	0.49915814	0.302686	0.964298	0.66702	0.834762	0.58437			
ESS-BS deletion Insertions:	67	1.1111111	0.888889	0.288889	0.638889	-0.5555556	0.638889	0.388889	-0.11111	0.138889	0.488889	0.836074	0.596215	0.125989
	67	0.01130359	0.251762	0.686924	0.408971	0.36032482	0.408971	0.614674	0.885534	0.8572	0.495692			
ESS-BS deletion Insertions/Total Insertions	68	0.0955556	0.055944	0.014644	0.045694	-0.0324444	0.029694	0.038694	0.004444	-0.00206	0.035044	0.411231	0.935817	0.066208
	68	0.01485818	0.419652	0.819561	0.509446	0.55032015	0.667844	0.576232	0.948756	0.976286	0.5855			
ESS-BS induction Insertions:	69	2.4444444	1.055556	1.155556	-0.19444	-0.2222222	0.305556	1.305556	0.555556	-0.44444	1.555556	1.722764	0.097215	0.229007
	69	1.3619E-06	0.208147	0.138577	0.815606	0.73411081	0.714106	0.120674	0.50585	0.594332	0.047614			
ESS-BS induction Insertions/Total Insertions	70	0.2151111	0.049139	0.133489	-0.03061	0.1105556	-0.00786	0.093639	0.063639	-0.02586	0.131689	1.189573	0.316793	0.170193
	70	5.4226E-05	0.586557	0.114443	0.734602	0.12175783	0.930596	0.301454	0.48157	0.77454	0.11932			
Splicing Change Insertions:	71	20.6666667	-4.16667	-2.66667	-3.66667	-10.222222	-3.66667	-8.16667	-6.41667	-8.66667	-9.86667	4.439507	0.00012	0.433567
	71	0	0.145675	0.313741	0.199611	1.905E-05	0.199611	0.005256	0.02662	0.003164	0.000364			
Splicing Change Insertions/Total Insertions	72	0.4558889	-0.06889	-0.03349	-0.02289	-0.0323333	0.032861	-0.05789	-0.00289	-0.10614	-0.08489	1.246009	0.282373	0.176839
	72	0	0.208689	0.508439	0.674544	0.45021825	0.546876	0.289933	0.957705	0.05466	0.096594			
Protein motif disrupting Insertions:	73	8.7777778	1.722222	0.222222	1.722222	-2.3333333	-0.02778	-1.27778	-1.52778	1.222222	1.022222	1.383075	0.211049	0.192546
	73	5E-12	0.365221	0.899531	0.365221	0.12008226	0.988312	0.501124	0.421537	0.519857	0.561869			
Protein motif disrupting Insertions/Total Insertions	74	0.8483333	0.046917	-0.03533	-0.02533	0.0415556	0.075417	-0.04658	0.057917	0.052167	0.118267	0.975219	0.474661	0.143939
	74	0	0.550346	0.627813	0.74685	0.50025282	0.338054	0.553175	0.461272	0.506778	0.107745			
**Conserved Element Deletions:**	75	843.333333	132.4167	-19.1333	18.41667	-482.44444	-371.083	-345.583	-352.333	-288.583	-365.133	43.44986	0	0.882233
	75	0	0.012664	0.69129	0.722721	0	7.22E-10	5.5E-09	3.22E-09	4.63E-07	1.21E-10			
TFBS Deletions:	76	4290.11111	-220.111	-423.911	-273.111	-2310.6667	-1926.36	-1827.36	-1940.86	-1519.11	-2098.11	57.45136	0	0.908302
	76	0	0.267298	0.023338	0.169746	0	0	0	0	7.7E-11	0			
TFBS Deletions/Total Deletions	77	0.6964444	-0.00169	0.003356	-0.00294	-0.0116667	-0.00594	-0.01319	-0.00644	5.56E-05	-0.00624	3.372554	0.001605	0.367679
	77	0	0.712471	0.432528	0.522332	0.00181544	0.1986	0.005306	0.163868	0.990354	0.146391			
miRNA-BS deletion Deletions:	78	20.4444444	8.555556	3.755556	6.055556	-14.333333	-9.94444	-9.69444	-7.94444	-7.69444	-10.0444	18.32486	0	0.759584
	78	0	0.003783	0.160532	0.037387	1.7103E-08	0.000866	0.00114	0.00693	0.008806	0.00032			
miRNA-BS deletion Deletions/Total Deletions	79	0.1102222	0.023778	0.018978	0.026778	-0.0304444	0.001028	-0.00047	0.009028	-0.00222	-0.00762	2.1182	0.037216	0.26751
	79	0	0.223515	0.294554	0.171066	0.04897341	0.957806	0.980606	0.642392	0.908928	0.672696			
miRNA-BS induction Deletions:	80	33.7777778	5.472222	0.222222	-1.27778	-21.111111	-18.2778	-17.5278	-14.5278	-14.7778	-16.3778	18.32268	0	0.759562
	80	0	0.129602	0.946672	0.721228	1.58E-10	2.72E-06	6.02E-06	0.000125	9.79E-05	5.32E-06			
miRNA-BS induction Deletions/Total Deletions	81	0.1836667	-0.00117	-0.00127	-0.02242	-0.0184444	-0.02292	-0.02192	-0.00117	-0.02067	-0.01307	0.521464	0.868092	0.082491
	81	0	0.956657	0.949311	0.298314	0.27551898	0.287755	0.309135	0.956657	0.337335	0.512625			
ESE-BS deletion Deletions:	82	26	4.5	-0.2	9.25	-15.222222	-7.75	-7.75	-8	-8.5	-10.6	9.093237	0	0.610562
	82	0	0.251167	0.955964	0.020198	4.5224E-06	0.050278	0.050278	0.043501	0.03228	0.004532			
ESE-BS deletion Deletions/Total Deletions	83	0.4051111	0.000889	-0.02731	0.056139	-0.0244444	0.002139	-0.02711	-0.01036	-0.05011	-0.04211	0.587014	0.817942	0.091908
	83	0	0.987061	0.591797	0.307633	0.57016432	0.968873	0.621191	0.85009	0.362095	0.409032			
ESE-BS induction Deletions:	84	28.4444444	7.555556	3.555556	1.305556	-16.666667	-7.44444	-4.19444	-9.69444	-8.19444	-9.44444	9.951237	0	0.631775
	84	0	0.050016	0.315229	0.731269	4.1309E-07	0.053383	0.271824	0.0127	0.033983	0.009063			
ESE-BS induction Deletions/Total Deletions	85	0.4477778	0.026972	0.010222	-0.06653	-0.0117778	0.009722	0.053722	-0.02928	-0.03478	2.22E-05	0.855087	0.579025	0.128486
	85	0	0.586149	0.823924	0.181763	0.76167589	0.844274	0.27978	0.554624	0.483015	0.999614			
ESS-BS deletion Deletions:	86	31.7777778	1.472222	4.222222	-0.27778	-17.666667	-10.7778	-9.02778	-11.0278	-12.7778	-11.1778	10.03397	0	0.633699
	86	0	0.700465	0.236793	0.942106	1.2712E-07	0.006166	0.020721	0.005129	0.001317	0.002367			
ESS-BS deletion Deletions/Total Deletions	87	0.5162222	-0.03422	-0.00682	-0.03047	-0.0238889	-0.00222	0.019028	0.011528	-0.07672	-0.01502	0.66591	0.75089	0.102988
	87	0	0.464093	0.874817	0.514291	0.51456306	0.962006	0.683544	0.804864	0.103461	0.728758			
ESS-BS induction Deletions:	88	18.7777778	4.472222	1.222222	3.222222	-11.444444	-6.02778	-4.27778	-7.77778	-6.27778	-6.77778	8.98993	0	0.607841
	88	0	0.113984	0.638801	0.252668	1.8663E-06	0.034479	0.130277	0.006951	0.027873	0.011019			
ESS-BS induction Deletions/Total Deletions	89	0.3084444	0.031806	-0.02104	0.019056	-0.0533333	0.017806	0.030806	-0.03669	-0.02119	-0.00844	1.027606	0.432303	0.150508
	89	0	0.482666	0.616449	0.673683	0.13599327	0.693944	0.496488	0.418264	0.639577	0.840582			
Splicing Change Deletions:	90	13.1111111	3.638889	4.288889	-0.11111	-6.3333333	-5.36111	-6.11111	-5.86111	-4.11111	-6.11111	7.863259	1E-07	0.575504
	90	0	0.110245	0.043757	0.960731	0.00062261	0.019937	0.008349	0.011249	0.071914	0.004651			
Splicing Change Deletions/Total Deletions	91	0.3514444	0.021306	0.057556	-0.02219	0.006	-0.03719	-0.04769	-0.04144	0.028556	-0.04484	0.806699	0.62298	0.122103
	91	0	0.713513	0.287085	0.702132	0.89509546	0.52199	0.412089	0.475735	0.622805	0.406076			
Protein motif disrupting Deletions:	92	6.7777778	1.472222	0.622222	1.722222	-2.8888889	0.972222	0.472222	-2.02778	1.222222	-1.17778	1.733008	0.094884	0.230055
	92	2.0505E-08	0.445951	0.728101	0.372984	0.05939529	0.614298	0.806493	0.294749	0.526613	0.510996			
Protein motif disrupting Deletions/Total Deletions	93	0.6297778	0.120972	0.069422	0.140722	0.2368889	0.184472	0.001722	0.026472	0.234972	0.210022	2.428602	0.017152	0.295142
	93	0	0.22137	0.448035	0.155679	0.00298924	0.064134	0.986031	0.787898	0.019295	0.024045			
**Conserved Element Rearrangements:**	94	274.666667	10.33333	-3.46667	-10.1667	-128.88889	-89.6667	-79.4167	-86.4167	-63.4167	-116.867	16.86356	0	0.744083
	94	0	0.619052	0.85727	0.624707	3.1E-11	5.02E-05	0.000276	8.72E-05	0.00313	6.27E-08			
TFBS Rearrangements:	95	1295.66667	-132.417	-184.867	-166.417	-700.22222	-567.417	-511.917	-557.667	-462.417	-662.467	39.01171	0	0.87057
	95	0	0.055131	0.004554	0.016773	0	5E-12	1.58E-10	1E-11	3.23E-09	0			
TFBS Rearrangements/Total Rearrangements	96	0.4974444	-0.00969	-0.01324	-0.00519	0.023	0.014306	0.004556	0.000556	0.012806	0.013956	5.877751	4.6E-06	0.503329
	96	0	0.217514	0.071349	0.507046	0.00035335	0.070646	0.560523	0.94334	0.104782	0.057754			
miRNA-BS deletion Rearrangements:	97	12	0.25	0.8	-0.25	-5.6666667	-8	-4.25	-4.5	-1.75	-6.6	11.69641	0	0.668504
	97	0	0.861213	0.547181	0.861213	3.3454E-06	4.07E-07	0.003972	0.002375	0.223545	4.52E-06			
miRNA-BS deletion Rearrangements/Total Rearrangements	98	0.2428889	0.010861	0.008511	0.031611	0.0246667	-0.07839	-0.01239	0.023611	0.037611	0.021911	2.620185	0.010594	0.311179
	98	0	0.703097	0.747579	0.269231	0.27174823	0.007394	0.663791	0.408295	0.189499	0.408391			
miRNA-BS induction Rearrangements:	99	10.6666667	2.833333	2.933333	-1.66667	-4.4444444	-5.16667	-0.16667	-5.41667	-1.16667	-4.66667	8.427784	0	0.592347
	99	0	0.084011	0.054595	0.305905	0.00081341	0.002111	0.918133	0.001318	0.472674	0.002728			
miRNA-BS induction Rearrangements/Total Rearrangements	100	0.2152222	0.067278	0.050378	0.005528	0.0548889	0.009278	0.093028	-0.03597	0.040528	0.080178	2.104322	0.038516	0.266224
	100	0	0.103151	0.187056	0.892419	0.09033734	0.820451	0.025503	0.380147	0.323151	0.037585			
ESE-BS deletion Rearrangements:	101	20.2222222	-2.47222	0.377778	-3.22222	-8.6666667	-5.72222	-3.72222	-7.47222	-5.22222	-11.6222	8.811149	0	0.603043
	101	0	0.242697	0.846727	0.129187	1.5809E-06	0.008125	0.080501	0.000682	0.01527	1E-07			
ESE-BS deletion Rearrangements/Total Rearrangements	102	0.4704444	-0.04594	-0.00984	-0.03994	0.0811111	-0.02494	-0.00494	-0.02469	0.008556	-0.06504	2.16782	0.032909	0.272072
	102	0	0.342556	0.826023	0.408885	0.0350667	0.60548	0.918371	0.609093	0.859257	0.149515			
ESE-BS induction Rearrangements:	103	19.6666667	-1.16667	1.133333	-1.16667	-11.111111	-4.41667	-4.91667	-5.91667	-7.66667	-10.4667	10.35605	0	0.641001
	103	0	0.604967	0.58829	0.604967	2.5477E-08	0.053263	0.031989	0.010421	0.001086	4.01E-06			
ESE-BS induction Rearrangements/Total Rearrangements	104	0.4592222	-0.01622	0.014978	0.009528	-0.0791111	-0.02347	-0.04647	0.017278	-0.07922	-0.01542	1.256853	0.276105	0.178104
	104	0	0.752672	0.753935	0.853132	0.05332344	0.648559	0.367951	0.737168	0.127002	0.746885			
ESS-BS deletion Rearrangements:	105	13.5555556	-0.55556	0.844444	-2.55556	-7	-4.05556	-0.80556	-4.80556	-6.05556	-9.15556	6.95712	5E-07	0.545352
	105	0	0.785408	0.655833	0.212933	4.107E-05	0.050043	0.693077	0.02095	0.004016	7.58E-06			
ESS-BS deletion Rearrangements/Total Rearrangements	106	0.4557778	-0.04553	0.039222	-0.04478	-0.0456667	-0.01153	0.104222	0.021222	-0.03003	-0.07778	2.423319	0.017381	0.294689
	106	0	0.352253	0.387709	0.360191	0.23522844	0.813235	0.035632	0.663767	0.538782	0.0893			
ESS-BS induction Rearrangements:	107	13.7777778	1.722222	-1.37778	-0.52778	-6	-4.02778	-4.77778	-4.52778	-4.52778	-7.57778	5.671252	7.2E-06	0.494388
	107	0	0.377516	0.446553	0.786271	0.00019339	0.041577	0.01629	0.022516	0.022516	7.75E-05			
ESS-BS induction Rearrangements/Total Rearrangements	108	0.4538889	0.060111	-0.01069	0.044361	0.0326667	-0.01839	-0.06464	0.035361	0.082111	0.071511	1.052359	0.413091	0.153576
	108	0	0.339828	0.854434	0.480495	0.50770911	0.769595	0.30496	0.573601	0.193593	0.222196			
Splicing Change Rearrangements:	109	2.1111111	0.138889	-0.91111	-0.36111	-0.8888889	-0.11111	2.638889	-0.61111	-0.86111	-1.91111	3.510176	0.001141	0.377026
	109	6.1251E-05	0.876404	0.273794	0.686091	0.20715785	0.900977	0.004173	0.494485	0.336538	0.023715			
Splicing Change Rearrangements/Total Rearrangements	110	0.3018889	-0.04014	-0.13969	-0.13914	-0.1028889	-0.08764	0.128361	0.052361	-0.10614	-0.25189	1.567239	0.139718	0.212731
	110	4.6049E-05	0.748855	0.232323	0.269109	0.29729137	0.485176	0.30763	0.676297	0.398319	0.033286			
Protein motif disrupting Rearrangements:	111	8.2222222	-0.47222	0.377778	-1.97222	-3.5555556	-0.97222	-2.72222	-3.97222	-2.22222	-4.62222	2.559656	0.012338	0.306192
	111	2E-12	0.784469	0.813631	0.25552	0.01042445	0.573723	0.118134	0.023984	0.200716	0.005103			
Protein motif disrupting Rearrangements/Total Rearrangements	112	0.7936667	0.026083	-0.01007	-0.14892	-0.1496667	-0.06817	-0.06917	-0.14242	-0.10817	-0.21487	1.067528	0.401584	0.155446
	112	0	0.825725	0.927041	0.211312	0.11060143	0.565405	0.559729	0.231696	0.362594	0.053939			
Nonsense SNPs:	113	6.1111111	2.888889	0.088889	1.638889	-2.3333333	-0.86111	0.888889	-1.11111	3.138889	2.088889	2.22405	0.028614	0.277173
	113	1.8972E-07	0.132382	0.959876	0.390563	0.12149068	0.651246	0.640793	0.559914	0.10257	0.239537			

Nonsense SNPs/Total SNPs	114	0.0137778	-0.00278	-0.00078	-0.00378	0.0041111	-0.00028	0.000472	0.000472	-0.00253	0.004222	1.081903	0.390873	0.15721
	114	4.5058E-08	0.49266	0.835777	0.351501	0.19753443	0.945206	0.906991	0.906991	0.532282	0.262621			
Frameshift Structural Variants:	115	43.2222222	3.777778	4.177778	5.027778	-15.444444	4.527778	-0.72222	-7.97222	2.527778	0.377778	4.395104	0.000134	0.4311
	115	0	0.489902	0.411059	0.358794	0.00057022	0.408295	0.894803	0.147556	0.643752	0.940597			
Frameshift Structural Variants/Total Variants	116	0.5041111	0.067389	0.016089	0.037389	0.1021111	0.122639	0.068639	0.022139	0.056639	0.140489	3.23888	0.002239	0.358328
	116	0	0.112893	0.680787	0.37598	0.00280577	0.004718	0.106491	0.599426	0.181518	0.000589			
Splicing Change Variants:	117	36.5555556	0.944444	1.044444	-3.05556	-17.333333	-8.55556	-10.8056	-12.8056	-12.3056	-16.9556	7.843573	1E-07	0.574891
	117	0	0.823137	0.790024	0.470389	1.6914E-06	0.046049	0.012489	0.003349	0.004715	4.92E-05			
Splicing Change Variants/Total Variants	118	0.2021111	-0.05161	0.002289	-0.06536	0.0084444	0.008639	-0.03036	0.005639	-0.08661	-0.05851	5.480469	0.000011	0.485837
	118	0	0.026748	0.914142	0.005511	0.63815936	0.705788	0.187226	0.805299	0.000314	0.007316			
Probably Damaging nscSNPs:	119	91.1111111	73.63889	0.088889	53.13889	-38.111111	-6.61111	28.88889	-16.3611	64.38889	17.08889	12.09586	0	0.675903
	119	0	2.24E-05	0.995286	0.001597	0.00369804	0.683569	0.078156	0.314647	0.000168	0.258324			
Probably Damaging nscSNPs/Total nscSNPs	120	0.3298889	0.010361	0.005511	-0.04089	0.0617778	0.013361	0.041111	0.024861	-0.01589	0.059711	5.026796	3.07E-05	0.464292
	120	0	0.645375	0.791889	0.072558	0.00079697	0.553093	0.071055	0.271295	0.480835	0.005483			
Possibly Damaging nscSNPs:	121	50.1111111	33.38889	-2.91111	24.63889	-28.888889	-8.61111	-0.36111	-12.1111	21.88889	-5.31111	9.384566	0	0.618033
	121	0	0.00088	0.744598	0.012405	0.00027459	0.372328	0.970068	0.2109	0.025609	0.552649			
Possibly Damaging nscSNPs/Total nscSNPs	122	0.1792222	-0.00772	-0.00502	-0.03272	-0.0166667	-0.01322	-0.02497	0.002028	-0.03522	-0.02022	1.276928	0.264792	0.180435
	122	0	0.648828	0.749569	0.056789	0.21255435	0.436235	0.143745	0.904746	0.040743	0.201277			
Protein motif damaging Variants:	123	53.7777778	25.97222	-3.77778	26.22222	-23.222222	2.722222	5.972222	-7.52778	23.97222	-0.37778	12.60038	0	0.684789
	123	0	0.000553	0.571584	0.000494	0.00010102	0.704963	0.407121	0.296803	0.001337	0.954832			
Protein motif damaging Variants/Total Variants	124	0.4148889	-0.03239	-0.00469	-0.02139	0.0493333	0.038111	-0.02189	0.041611	-0.04614	0.007511	1.628075	0.1214	0.219179
	124	0	0.410232	0.89755	0.586034	0.11232001	0.33303	0.577327	0.290868	0.241988	0.836607			
TFBS Disrupting Variants:	125	6797.11111	-419.611	-743.111	-481.611	-3627.7778	-2941.11	-2736.61	-2954.61	-2244.86	-3310.71	53.31138	0	0.90188
	125	0	0.184801	0.012846	0.128785	0	0	1E-12	0	7.56E-10	0			
TFBS Disrupting Variants/Total Variants	126	0.2641111	-0.07386	-0.01071	-0.07161	0.0288889	-0.01836	-0.04636	-0.01661	-0.09111	-0.04951	8.212642	0	0.586088
	126	0	0.000861	0.587445	0.001201	0.08648927	0.388801	0.031992	0.435361	5.58E-05	0.014125			
miRNA-BS Disrupting Variants:	127	339.111111	182.6389	7.488889	258.6389	-197.88889	-107.361	-53.6111	-120.611	137.1389	-76.7111	21.34301	0	0.786317
	127	0	0.000256	0.865046	7.24E-07	1.2213E-06	0.026416	0.260985	0.013052	0.005044	0.085115			
miRNA-BS Disrupting Variants/Total Variants	128	0.3733333	0.033667	0.014867	0.043667	-0.0283333	0.024917	0.022667	-0.00258	0.059667	0.022667	4.625543	7.76E-05	0.443674
	128	0	0.080894	0.40214	0.024648	0.06156037	0.19411	0.236991	0.892235	0.002506	0.203032			
ESE-BS Disrupting Variants:	129	414.333333	254.6667	14.06667	274.4167	-224.66667	-80.0833	14.66667	-109.333	206.6667	-43.3333	18.96265	0	0.765776
	129	0	3.89E-05	0.793973	1.12E-05	5.1874E-06	0.170483	0.800465	0.062867	0.000654	0.422109			
ESE-BS Disrupting Variants/Total Variants	130	0.8411111	0.014389	0.009489	0.007889	0.0063333	-0.00361	0.014889	0.015139	0.003139	0.013489	0.801458	0.627774	0.121406
	130	0	0.193363	0.353918	0.47381	0.46356824	0.742624	0.178531	0.171447	0.775287	0.189013			
ESS-BS Disrupting Variants:	131	248	144.25	0.6	146.5	-132.44444	-50.25	-0.5	-66.5	110.75	-32	18.48948	0	0.761214
	131	0	4.78E-05	0.984512	3.76E-05	3.1234E-06	0.134554	0.98802	0.049059	0.001379	0.302446			
ESS-BS Disrupting Variants/Total Variants	132	0.8857778	0.026222	0.005222	0.013472	-0.0255556	0.007722	0.028472	0.009472	0.021972	0.016822	1.937053	0.05808	0.250361
	132	0	0.184176	0.774307	0.492951	0.10017556	0.693979	0.149792	0.629461	0.264871	0.357035			
**Total Likely Functional Variants:**	133	7282.88889	142.6111	-630.489	136.3611	-3907.4444	-2977.14	-2657.14	-3068.14	-1745.64	-3248.29	45.28719	0	0.886469
	133	0	0.708934	0.078694	0.721134	0	5E-11	1.61E-09	1.8E-11	2.01E-05	0			
**Total variants (not just novel)**	134	4724247.78	-196430	-202822	-164159	-893038.22	-841090	-789075	-817815	-743688	-957660	184.3733	0	0.969502
	134	0	3.73E-05	6.04E-06	0.000449	0	0	0	0	0	0			
synonymous to nonsynonymous rate	135	0.6586091	0.02646	0.068551	0.063024	-0.1145034	-0.16064	-0.08688	-0.0454	-0.01055	-0.09287	7.395467	2E-07	0.560455
	135	0	0.535672	0.086847	0.142783	0.00102305	0.000337	0.044858	0.289276	0.804753	0.021496			
Likely functional variant/Total Novel Variants	136	0.0708761	-0.01828	-0.0006	-0.01815	0.0066739	-0.00518	-0.01145	-0.00417	-0.02201	-0.01301	7.203966	3E-07	0.553982
	136	0	0.001694	0.908374	0.001824	0.13264981	0.357626	0.044495	0.457724	0.000198	0.014557			
Likely functional variant (minus probdam)/ Total Novel Variants	137	0.0703958	-0.01839	-0.00061	-0.01816	0.0067091	-0.00532	-0.0116	-0.00428	-0.02217	-0.01317	7.203754	3E-07	0.553975
	137	0	0.001693	0.907453	0.001916	0.1328733	0.34723	0.042888	0.448967	0.000195	0.01401			
Functional Nonsense SNPs/Total Novel Variants	138	0.0000607	3.3E-06	3.7E-06	-4.2E-06	0.0000177	1.87E-05	2.95E-05	1.52E-05	2.03E-05	5.37E-05	1.197579	0.311726	0.171142
	138	1.5714E-05	0.889325	0.866739	0.859729	0.33892462	0.428362	0.213532	0.519396	0.390899	0.016351			
Functional Frameshift Structural Variants/Total Novel Variants	139	0.0004226	-8.9E-05	7.95E-05	-7.6E-05	0.0002091	0.000306	0.000121	0.000136	-1.9E-05	0.000196	5.046118	2.94E-05	0.465247
	139	1E-12	0.301841	0.320884	0.380634	0.00273506	0.000654	0.161709	0.1161	0.82382	0.016205			
Functional Splicing Change Variants/Total Novel Variants	140	0.0003553	-8.9E-05	3.68E-05	-0.00012	0.0000927	7.79E-05	-2.4E-05	2.39E-05	-0.00014	-7.9E-05	4.032949	0.000318	0.410146
	140	0	0.157674	0.525705	0.049499	0.06145347	0.214658	0.698679	0.701898	0.026954	0.174216			
Probably Damaging nscSNPs/Total Novel Variants	141	0.0008836	0.000284	6.58E-05	8.68E-05	0.0002087	0.000391	0.000653	0.000269	0.000489	0.000652	7.85168	1E-07	0.575144
	141	0	0.030645	0.582553	0.501693	0.04216831	0.003361	3.22E-06	0.04026	0.000307	7.29E-07			
Possibly Damaging nscSNPs/Total Novel Variants	142	0.0004803	0.000108	9.6E-06	1.54E-05	-0.0000353	0.000145	0.000158	0.000107	0.000153	0.000154	2.825048	0.006324	0.32754
	142	0	0.142361	0.88695	0.833044	0.53760443	0.050049	0.032739	0.144709	0.038784	0.025637			
Functional Protein motif damaging Variants/Total Novel Variants	143	0.0005225	4.31E-05	7E-07	3.41E-05	0.0001505	0.000337	0.000243	0.000201	0.000164	0.00024	5.630639	7.9E-06	0.492592
	143	0	0.560141	0.991777	0.64519	0.01134166	2.13E-05	0.00158	0.008231	0.029006	0.000826			
Functional TFBS Disrupting Variants/Total Novel Variants	144	0.0662327	-0.02106	-0.0022	-0.02087	0.0073769	-0.00731	-0.01404	-0.0053	-0.02605	-0.01617	7.492319	2E-07	0.563658
	144	0	0.001439	0.709088	0.001577	0.14235051	0.252717	0.030027	0.40613	0.000109	0.007646			
Functional miRNA-BS Disrupting Variants/Total Novel Variants	145	0.0032617	0.000436	0.000384	0.000795	-0.0003134	0.000246	0.000408	0.000153	0.000937	0.000507	6.381019	1.6E-06	0.523849
	145	0	0.070377	0.085644	0.001305	0.09651807	0.303292	0.090073	0.520888	0.000188	0.024235			
Functional ESE-BS Disrupting Variants/Total Novel Variants	146	0.0039868	0.000752	0.000497	0.000709	-0.0000033	0.001053	0.001512	0.000762	0.001495	0.001287	8.455206	0	0.593131
	146	0	0.018311	0.089651	0.025724	0.98938286	0.001176	7.27E-06	0.016804	8.89E-06	3.17E-05			
Functional ESS-BS Disrupting Variants/Total Novel Variants	147	0.0023907	0.000385	0.000224	0.000292	0.0000725	0.000584	0.000779	0.000432	0.000779	0.000691	8.660431	0	0.598905
	147	0	0.015743	0.124942	0.064863	0.55429776	0.000359	4.16E-06	0.007068	4.17E-06	9.6E-06			
Likely functional variant/Total Variants	148	0.0015389	0.000101	-6.9E-05	8.55E-05	-0.0006583	-0.00043	-0.00036	-0.00046	-0.00015	-0.00047	33.55282	0	0.852615
	148	0	0.184136	0.323636	0.25965	0	2.68E-07	8.15E-06	5.4E-08	0.053055	4.86E-09			
Likely functional variant (minus probdam)/Total Variants	149	0.0015283	0.000093	-6.9E-05	7.97E-05	-0.0006532	-0.00043	-0.00037	-0.00046	-0.00016	-0.00047	34.26355	0	0.85523
	149	0	0.210817	0.314632	0.282899	0	1.66E-07	5.02E-06	3.38E-08	0.038366	2.52E-09			
Functional Nonsense SNPs/Total Variants	150	0.0000013	7E-07	1E-07	4E-07	-0.0000003	1E-07	5E-07	0	0.000001	9E-07	2.169762	0.03275	0.272249
	150	4.2131E-06	0.141273	0.850102	0.38795	0.40011438	0.904632	0.298589	0.974043	0.031218	0.04596			
Functional Frameshift Structural Variants/Total Variants	151	0.0000091	1.3E-06	1.3E-06	1.4E-06	-0.0000019	3.2E-06	1.7E-06	-1E-07	2.4E-06	2.4E-06	3.939877	0.000398	0.40451
	151	0	0.32676	0.268558	0.260307	0.06031278	0.014685	0.1909	0.930864	0.067033	0.043474			
Functional Splicing Change Variants/Total Variants	152	0.0000077	5E-07	6E-07	-4E-07	-0.0000027	-5E-07	-1.2E-06	-1.7E-06	-1.6E-06	-2.5E-06	4.008284	0.000337	0.408663
	152	0	0.574425	0.528115	0.675337	0.00061704	0.587721	0.223534	0.09227	0.095344	0.006239			
Probably Damaging nscSNPs/Total Variants	153	0.0000193	1.71E-05	9E-07	1.22E-05	-0.0000055	2.5E-06	1.12E-05	-2E-07	1.98E-05	9.4E-06	12.05476	0	0.675157
	153	0	1.96E-05	0.789612	0.001603	0.06561241	0.509218	0.003619	0.963559	1.29E-06	0.008297			
Possibly Damaging nscSNPs/Total Variants	154	0.0000106	7.9E-06	-1E-07	5.8E-06	-0.000005	1E-07	2.1E-06	-9E-07	7.5E-06	1.3E-06	8.699868	0	0.599996
	154	1E-12	0.000538	0.945809	0.009665	0.0040806	0.963134	0.342401	0.692073	0.000887	0.51923			
Functional Protein motif damaging Variants/Total Variants	155	0.0000114	6.3E-06	-3E-07	6.1E-06	-0.0000034	3.2E-06	3.8E-06	5E-07	8.2E-06	2.8E-06	11.54312	0	0.665574
	155	0	0.000376	0.8463	0.000473	0.01202163	0.060927	0.025154	0.780798	6.66E-06	0.077943			
Functional TFBS Disrupting Variants/Total Variants	156	0.0014363	-2.8E-05	-9.9E-05	-5.3E-05	-0.0006092	-0.00044	-0.0004	-0.00045	-0.00029	-0.00051	39.51957	0	0.87202
	156	0	0.643866	0.078645	0.379672	0	3.05E-10	4.39E-09	1.56E-10	6.86E-06	0			
Functional miRNA-BS Disrupting Variants/Total Variants	157	0.0000716	4.36E-05	5.2E-06	5.91E-05	-0.0000348	-1.2E-05	9E-07	-1.6E-05	0.000048	-2E-06	19.69532	0	0.772507
	157	0	8.24E-05	0.594041	3.12E-07	6.2226E-05	0.252596	0.930271	0.134381	1.82E-05	0.839643			
Functional ESE-BS Disrupting Variants/Total Variants	158	0.0000875	6.02E-05	7.3E-06	6.31E-05	-0.0000381	-1.5E-06	2.15E-05	-9.5E-06	6.85E-05	1.08E-05	17.76932	0	0.753917
	158	0	1.53E-05	0.543226	6.75E-06	0.00035211	0.907008	0.100484	0.463043	1.36E-06	0.369367			
Functional ESS-BS Disrupting Variants/Total Variants	159	0.0000524	3.42E-05	2.6E-06	3.38E-05	-0.0000223	-1.5E-06	1.05E-05	-6E-06	3.78E-05	4.9E-06	17.05222	0	0.746195
	159	0	1.8E-05	0.705308	2.15E-05	0.00028178	0.83872	0.160708	0.42186	3E-06	0.478442			
