# Supplementary material for: Clinical Implications of Human Population Differences in Genome-Wide Rates of Functional Genotypes
Source: Front Genet. 2012 Nov 1;3:211. doi: 10.3389/fgene.2012.00211 (PMC3485509; doi:10.3389/fgene.2012.00211)
Supplement: Supplementary Data Sheet S3 — Regression analysis results for ancestral allele-based variants: all variants. [file 32001_Schork_DataSheet3.PDF]

Ancestral Allele Based Variants: All Variants

Row 1: Regression Coefficients

Row 2: P-values for Regression Coefficients

	Var Cat	Y-int	LWK	ASW	MKK	CEU	TSI	CHB	JPT	GIH	MEX	Overall F	p-val	R-Sqr
Coding SNPs:	1	35799.7778	16.72222	38.22222	-65.2778	-1698	-1541.28	-2006.03	-1971.03	-1404.53	-1715.58	205.7367	0	0.972582
	1	0	0.863279	0.671699	0.502121	0	0	0	0	0	0			
Nonsynonymous SNPs:	2	17321	31.5	50.2	-26.25	-726.11111	-637.25	-890.25	-895.75	-617.75	-757.2	110.0118	0	0.949919
	2	0	0.595025	0.362402	0.657681	0	0	0	0	0	0			
Synonymous SNPs:	3	18361.6667	-21.9167	-15.0667	-34.1667	-959.44444	-892.417	-1105.42	-1063.17	-774.167	-953.267	182.1735	0	0.969145
	3	0	0.698662	0.774271	0.546495	0	0	0	0	0	0			
Nonsense SNPs:	4	117.111111	7.138889	3.088889	-4.86111	-12.444444	-11.6111	-10.3611	-12.1111	-12.6111	-5.11111	4.831018	4.81E-05	0.454427
	4	0	0.150484	0.500097	0.325542	0.00190769	0.0209	0.03851	0.016167	0.01242	0.265911			
Untranslated Region SNPs:	5	50171.7778	-51.7778	143.6222	-167.778	-2949.8889	-2759.53	-3456.28	-3422.53	-2421.78	-3005.58	415.4529	0	0.986232
	5	0	0.663004	0.195323	0.160729	0	0	0	0	0	0			
Non-coding RNA SNPs:	6	1558466.22	-2587.72	-694.822	-5134.72	-83312.889	-82300.7	-99965	-102606	-75908.2	-87728.4	478.1547	0	0.988015
	6	0	0.417885	0.814306	0.110483	0	0	0	0	0	0			
Intronic SNPs:	7	2183021.22	-2703.47	1812.978	-8367.97	-125213.44	-120143	-148104	-148981	-110609	-127590	688.5345	0	0.991647
	7	0	0.49242	0.619623	0.036293	0	0	0	0	0	0			
Intergenic SNPs:	8	3037452.78	-1583.53	581.8222	-12240.5	-170147	-165084	-205702	-205537	-149866	-179314	567.9633	0	0.989891
	8	0	0.790476	0.916219	0.043089	0	0	0	0	0	0			
**Total SNPs:**	9	5317957.67	-4277.92	2656.733	-20806.9	-300523.22	-289996	-359864	-360590	-264746	-312155	680.6113	0	0.99155
	9	0	0.65478	0.764718	0.032427	0	0	0	0	0	0			
Coding Insertions:	10	345	-22.25	-18.6	-21.75	-46	-26.75	-26	-28	-20.25	-42.8	7.473424	2E-07	0.563037
	10	0	0.013791	0.025928	0.015966	5.98E-09	0.003366	0.004305	0.002212	0.024457	1.74E-06			
In-frame Insertions:	11	79.4444444	-4.44444	-4.24444	-5.44444	-7.5555556	-7.69444	-7.69444	-6.44444	-5.19444	-10.0444	3.797133	0.000563	0.395653
	11	0	0.077634	0.069628	0.03161	0.0002381	0.002808	0.002808	0.011503	0.04	4.53E-05			
Out-of-frame Insertions:	12	61.6666667	-15.4167	-8.86667	-7.16667	-9	-4.66667	-7.66667	-7.16667	-5.66667	-15.8667	4.378566	0.000139	0.430175
	12	0	8.03E-05	0.011366	0.055012	0.00261794	0.207895	0.040497	0.055012	0.127264	1.56E-05			
Frameshift Insertions:	13	203.888889	-2.38889	-5.48889	-9.13889	-29.444444	-14.3889	-10.6389	-14.3889	-9.38889	-16.8889	6.848707	6E-07	0.541455
	13	0	0.688913	0.323174	0.128722	2.4705E-08	0.018158	0.077873	0.018158	0.118765	0.003159			
Untranslated region Insertions:	14	3771.88889	-336.389	-209.489	-88.1389	-381.33333	-318.889	-286.639	-305.889	-231.889	-508.889	14.68989	0	0.716934
	14	0	2.04E-06	0.00086	0.177512	1.79E-10	5.68E-06	3.55E-05	1.2E-05	0.000634	3E-12			
Non-coding RNA Insertions:	15	90270.8889	-9603.89	-6991.69	-3174.89	-9703.1111	-8636.14	-6568.39	-8302.39	-6454.89	-14449.1	12.87613	0	0.689443
	15	0	2.25E-06	0.000131	0.091742	5.992E-09	1.59E-05	0.000734	3.05E-05	0.000891	5E-12			
Intronic Insertions:	16	136840.222	-14891.5	-11042.8	-5232.72	-15552.333	-13681.7	-10878.2	-13350.7	-10594	-22530.4	13.73643	0	0.703119
	16	0	1.45E-06	7.36E-05	0.067477	1.275E-09	7.42E-06	0.000254	1.15E-05	0.000356	2E-12			
Intergenic Insertions:	17	176232	-19358	-14198.8	-6380	-19798.444	-17534.3	-13335.3	-16696.8	-13162.8	-29314.4	13.5674	0	0.700528
	17	0	1.55E-06	9.04E-05	0.086821	2.535E-09	1.01E-05	0.000545	2.33E-05	0.000634	2E-12			
**Total Insertions:**	18	317694.667	-34639.9	-25500.5	-11737.7	-35833.222	-31617.4	-24581.2	-30435.9	-24049.4	-52470.7	13.70162	0	0.702589
	18	0	1.47E-06	8.26E-05	0.077758	1.706E-09	8.43E-06	0.000369	1.64E-05	0.000482	2E-12			
Coding Deletions:	19	295.666667	-7.41667	-6.06667	-9.66667	-45.444444	-39.1667	-35.6667	-26.6667	-15.1667	-53.0667	18.72991	0	0.763554
	19	0	0.299292	0.359913	0.177318	1.2E-11	5.84E-07	3.9E-06	0.000358	0.036072	1.8E-11			
In-frame Deletions:	20	28.1111111	-0.61111	-2.11111	-2.36111	-4.4444444	-5.11111	-3.61111	-2.11111	1.638889	-7.31111	4.431514	0.000123	0.433124
	20	0	0.749109	0.236223	0.219027	0.00404545	0.009112	0.062058	0.271236	0.392187	9.96E-05			
Inter-Codon Deletions:	21	44.2222222	-3.97222	-3.02222	1.527778	-8	-9.47222	-3.97222	-2.22222	-3.97222	-10.4222	4.058833	0.000298	0.411695
	21	0	0.194067	0.286107	0.61554	0.00125914	0.002604	0.194067	0.465588	0.194067	0.000426			
Frameshift Deletions:	22	223.333333	-2.83333	-0.93333	-8.83333	-33	-24.5833	-28.0833	-22.3333	-12.8333	-35.3333	15.44902	0	0.727046
	22	0	0.616964	0.858998	0.121915	2.26E-10	4.59E-05	4.71E-06	0.000183	0.026046	4.21E-09			
Untranslated region Deletions:	23	3344.44444	-296.194	-238.644	-183.944	-516.11111	-461.444	-399.444	-453.944	-411.694	-661.844	31.99298	0	0.846532
	23	0	2.66E-06	3.28E-05	0.002188	0	2.5E-11	2.11E-09	4.2E-11	8.79E-10	0			
Non-coding RNA Deletions:	24	87172.8889	-10050.6	-7829.89	-3699.14	-12290.111	-11336.1	-8887.64	-10647.9	-9240.64	-17093.1	16.64671	0	0.74161
	24	0	3.19E-06	6.37E-05	0.065704	3.3E-11	2.56E-07	2.82E-05	1E-06	1.47E-05	0			
Intronic Deletions:	25	129724.889	-15528.4	-12203.7	-6375.89	-19281.556	-17765.1	-14388.6	-16783.6	-14298.4	-26628.5	17.94235	0	0.755711
	25	0	1.71E-06	3.42E-05	0.034817	7E-12	8.78E-08	7.36E-06	3.28E-07	8.24E-06	0			
Intergenic Deletions:	26	170550.667	-20228.9	-16043.5	-7955.92	-24847.889	-22894.7	-18177.2	-21586.7	-18603.7	-34765.5	17.5996	0	0.752133
	26	0	1.99E-06	3.33E-05	0.044496	1.3E-11	1.36E-07	1.45E-05	5.14E-07	9.66E-06	0			
**Total Deletions:**	27	304351	-36080.8	-28498.2	-14545.5	-44749.444	-41227.3	-33050.3	-38900	-33369.5	-62172.6	17.92579	0	0.75554
	27	0	1.83E-06	3.33E-05	0.038766	8E-12	9.8E-08	9.61E-06	3.74E-07	8.09E-06	0			
Coding rearrangements:	28	435.777778	-33.0278	-15.1778	-11.0278	-41.666667	-21.2778	-18.2778	-23.2778	-15.2778	-52.3778	7.962408	1E-07	0.578562
	28	0	0.000714	0.08352	0.240287	2.8506E-07	0.025422	0.053716	0.01484	0.105402	6.9E-08			
In-frame rearrangements:	29	412.888889	-30.3889	-18.0889	-13.6389	-39.555556	-24.1389	-21.6389	-22.3889	-16.1389	-49.4889	7.908643	1E-07	0.576909
	29	0	0.000711	0.026036	0.115869	1.38E-07	0.006322	0.013858	0.011013	0.06377	3.56E-08			
Frameshift rearrangements:	30	22.8888889	-2.63889	2.911111	2.611111	-2.1111111	2.861111	3.361111	-0.88889	0.861111	-2.88889	3.040397	0.00368	0.343921
	30	0	0.208067	0.135547	0.212837	0.19930757	0.172725	0.110133	0.669893	0.679614	0.138507			
Untranslated region rearrangements:	31	853.222222	-75.2222	-53.4222	-32.4722	-132.88889	-92.2222	-91.9722	-106.222	-84.4722	-159.622	38.85905	0	0.870127
	31	0	1.98E-07	3.48E-05	0.014746	0	9.64E-10	1.04E-09	1.1E-11	1.12E-08	0			
Non-coding RNA rearrangements:	32	31815.1111	-2568.61	-1796.91	-1219.36	-4098	-3478.86	-2946.36	-3536.11	-2958.61	-5343.11	23.3604	0	0.8011
	32	0	1.1E-05	0.000639	0.02729	0	1.51E-08	7.7E-07	9.81E-09	7.05E-07	0			
Intronic rearrangements:	33	40575.5556	-3415.81	-2564.76	-1768.06	-5984.5556	-5244.31	-4748.81	-5269.56	-4488.06	-7378.56	33.37774	0	0.851957
	33	0	1.7E-06	6.87E-05	0.008385	0	1.9E-11	4.45E-10	1.6E-11	2.33E-09	0			
Intergenic rearrangements:	34	60155.4444	-4880.44	-3496.24	-2238.44	-8225.8889	-7059.19	-5988.69	-7026.69	-5928.94	-10499	27.15747	0	0.824016
	34	0	7.17E-06	0.000362	0.028893	0	1.27E-09	9.84E-08	1.46E-09	1.25E-07	0			
**Total rearrangements:**	35	102267.667	-8420.67	-6146.07	-4059.17	-14410.444	-12442.2	-10868.7	-12452.4	-10536.4	-18123.1	29.89444	0	0.83751
	35	0	3.49E-06	0.000173	0.017385	0	2.12E-10	1.04E-08	2.06E-10	2.34E-08	0			
**Total number of variants:**	36	6042271	-83419.3	-57488	-51149.3	-395516.33	-375283	-428364	-442378	-332702	-444922	222.3361	0	0.974577
	36	0	8.19E-05	0.002709	0.012324	0	0	0	0	0	0			
Conserved Element SNPs:	37	238986.778	-327.778	-244.778	-1434.78	-17205.333	-16684.8	-19406.3	-19506.8	-15104.3	-18058	871.1452	0	0.993386
	37	0	0.48626	0.575108	0.003138	0	0	0	0	0	0			
TFBS SNPs:	38	30.6666667	-2.91667	-1.06667	-2.66667	-7.6666667	-6.16667	-2.16667	-1.16667	-1.91667	-8.86667	3.10625	0.00312	0.348772
	38	0	0.312279	0.689586	0.355267	0.00109914	0.034954	0.452106	0.685121	0.505758	0.001395			
TFBS SNPs/Total SNPs	39	0	0	0	0	0	0	0	0	0	0	0	1	0
	39													
miRNA-BS disrupting SNPs:	40	189.111111	-4.11111	-7.31111	-12.8611	-43	-35.3611	-39.3611	-40.8611	-33.6111	-57.5111	43.08068	0	0.881344
	40	0	0.397923	0.107782	0.009727	0	4.08E-10	1.3E-11	4E-12	1.82E-09	0			
miRNA-BS disrupting SNPs/Total SNPs	41	0.0325556	-0.00106	-0.00156	-0.00231	-0.0054444	-0.00406	-0.00456	-0.00481	-0.00406	-0.00776	17.49803	0	0.751052
	41	0	0.255245	0.07293	0.014629	1.59E-10	3.85E-05	5.24E-06	1.86E-06	3.85E-05	0			
ESE-BS deletion SNPs:	42	1225.11111	-9.11111	1.288889	-16.3611	-45.333333	-53.6111	-79.3611	-68.6111	-57.6111	-46.3111	19.34018	0	0.769294
	42	0	0.36718	0.890361	0.107718	2.3199E-07	1.18E-06	3.5E-11	2.96E-09	2.49E-07	4.87E-06			
ESE-BS deletion SNPs/Total SNPs	43	0.0503333	-0.00058	-0.00033	-0.00058	0.0005556	-8.3E-05	-0.00033	-8.3E-05	-0.00058	0.000667	3.122412	0.002996	0.349952
	43	0	0.14974	0.372884	0.14974	0.08143096	0.83573	0.407989	0.83573	0.14974	0.077296			
ESE-BS induction SNPs:	44	1449.44444	-2.69444	2.555556	-10.4444	-56.777778	-51.1944	-85.6944	-62.6944	-48.6944	-47.4444	11.02826	0	0.655342
	44	0	0.850462	0.847238	0.465738	3.1874E-06	0.000613	8.21E-08	3.92E-05	0.001069	0.000624			
ESE-BS induction SNPs/Total SNPs	45	0.0594444	-0.00019	-4.4E-05	-0.00044	0.0004444	0.000556	-0.00019	0.000806	0.000306	0.000956	1.278685	0.26382	0.180639
	45	0	0.74283	0.935578	0.454047	0.34051789	0.349914	0.74283	0.176839	0.606349	0.085683			
ESS-BS deletion SNPs:	46	475.111111	-5.36111	10.68889	9.388889	1.7777778	4.388889	-7.61111	-19.8611	0.138889	-11.3111	5.318152	1.59E-05	0.478331
	46	0	0.353947	0.049001	0.106798	0.69440715	0.447455	0.18961	0.00095	0.980779	0.037559			
ESS-BS deletion SNPs/Total SNPs	47	0.0532222	-0.00072	0.001178	0.000778	0.0023333	0.002778	0.002028	0.000528	0.002028	0.000778	6.449892	1.4E-06	0.526526
	47	0	0.285004	0.062602	0.249895	3.4613E-05	9.75E-05	0.003513	0.433709	0.003513	0.215479			
ESS-BS induction SNPs:	48	504.666667	6.083333	0.133333	18.58333	-15.666667	-18.1667	-22.1667	-20.1667	-20.4167	-27.0667	6.337865	1.7E-06	0.522157
	48	0	0.476149	0.986551	0.032095	0.02160137	0.036016	0.011131	0.0204	0.01895	0.001022			
ESS-BS induction SNPs/Total SNPs	49	0.0565556	0.000444	-0.00056	0.001694	0.0004444	-5.6E-05	0.000444	0.000694	-0.00031	-0.00096	1.376949	0.213881	0.191857
	49	0	0.620181	0.504823	0.06197	0.52778073	0.950558	0.620181	0.439321	0.733189	0.252879			
Splicing Change SNPs:	50	2404.11111	49.63889	57.68889	35.63889	95.2222222	88.38889	23.38889	26.63889	66.13889	112.8889	19.80717	0	0.773501
	50	0	0.00047	1.9E-05	0.010292	0	9.65E-09	0.087723	0.052549	6.37E-06	0			
Splicing Change SNPs/Total SNPs	51	0.3325556	0.008944	0.007444	0.007194	0.0337778	0.031944	0.027444	0.029194	0.026694	0.034444	131.0988	0	0.957633
	51	0	1.43E-05	8.11E-05	0.000352	0	0	0	0	0	0			
Protein Motif disrupting SNPs:	52	843.444444	-4.19444	-12.0444	-9.44444	-84.888889	-64.6944	-69.9444	-79.1944	-65.1944	-79.0444	14.94784	0	0.720453
	52	0	0.777953	0.384213	0.52597	4.31E-10	4.47E-05	1.24E-05	1.17E-06	3.97E-05	2.42E-07			
Protein Motif disrupting SNPs/Total SNPs	53	0.1765556	-0.00081	-0.00276	-0.00056	-0.0087778	-0.00631	-0.00481	-0.00756	-0.00656	-0.00756	4.51006	0.000102	0.437443
	53	0	0.754083	0.250485	0.828923	4.437E-05	0.01639	0.064951	0.004371	0.012736	0.002241			
Conserved Element Insertions:	54	13761.5556	-1055.81	-771.156	-362.806	-1419.1111	-1212.31	-1047.56	-1153.31	-950.056	-1888.56	17.60785	0	0.75222
	54	0	7.03E-06	0.00028	0.098633	6E-12	4.4E-07	8.1E-06	1.27E-06	4.18E-05	0			
TFBS Insertions:	55	80	-10.25	-7.8	-4.5	-17	-9.5	-14.25	-9.5	-5	-23.2	9.053092	0	0.609509
	55	0	0.007767	0.027724	0.232452	1.9466E-07	0.013277	0.000299	0.013277	0.185141	5.35E-09			
TFBS Insertions/Total Insertions	56	0.0021111	0.000139	0.000289	0.000139	-0.0001111	0.000389	-0.00011	0.000389	0.000639	-0.00011	2.646033	0.009926	0.313287
	56	0	0.553895	0.186986	0.553895	0.5460932	0.100422	0.635663	0.100422	0.007939	0.609803			
miRNA-BS disrupting Insertions:	57	8.8888889	-0.38889	-0.28889	0.111111	-1.3333333	-0.88889	-1.63889	-1.88889	-2.13889	-1.68889	5.727471	6.4E-06	0.496854
	57	0	0.458307	0.552516	0.831874	0.00175144	0.092822	0.002486	0.000561	0.000113	0.000859			
miRNA-BS disrupting Insertions/Total Insertions	58	0.0101111	0.000389	0.000289	0.000389	-0.0004444	-0.00011	-0.00086	-0.00136	-0.00211	-0.00091	3.487147	0.001208	0.375481
	58	0	0.531165	0.616053	0.531165	0.3623857	0.857809	0.167959	0.031021	0.00108	0.116787			
ESE-BS deletion Insertions:	59	14.8888889	0.611111	0.711111	-0.13889	0.1111111	0.611111	-1.63889	-1.88889	1.361111	-0.28889	6.661194	9E-07	0.534555
	59	0	0.259165	0.158312	0.796705	0.79277203	0.259165	0.003258	0.000787	0.013597	0.564133			
ESE-BS deletion Insertions/Total Insertions	60	0.0307778	0.002222	0.001822	0.000472	0.0016667	0.001972	-0.00278	-0.00278	0.003222	0.000422	5.100145	0.000026	0.467897
	60	0	0.077989	0.118527	0.704886	0.09165905	0.116874	0.028592	0.028592	0.0116	0.715228			
ESE-BS induction Insertions:	61	31.5555556	-0.30556	1.244444	0.194444	1.4444444	1.194444	0.944444	0.444444	1.944444	2.044444	1.996276	0.050255	0.256055
	61	0	0.727182	0.128946	0.824257	0.03848984	0.175411	0.282747	0.612015	0.029141	0.013927			
ESE-BS induction Insertions/Total Insertions	62	0.0655556	0.000694	0.003044	0.001944	0.0057778	0.003444	0.003194	0.003444	0.004194	0.006044	3.095503	0.003205	0.347985
	62	0	0.718444	0.091878	0.314336	0.00027545	0.07703	0.100484	0.07703	0.032248	0.001157			
ESS-BS deletion Insertions:	63	54.4444444	2.055556	1.555556	1.055556	2.2222222	3.305556	2.055556	0.305556	2.055556	1.355556	1.789952	0.082853	0.235832
	63	0	0.080954	0.153215	0.366133	0.01725557	0.005813	0.080954	0.793055	0.080954	0.21243			
ESS-BS deletion Insertions/Total Insertions	64	0.1172222	0.004528	0.004178	0.002778	0.0071111	0.007028	0.005528	0.004028	0.006528	0.004778	2.006752	0.04898	0.257053
	64	0	0.103758	0.105778	0.315241	0.00154151	0.012728	0.048069	0.146997	0.020272	0.065143			
ESS-BS induction Insertions:	65	22.4444444	0.055556	-0.04444	-0.19444	-0.6666667	-1.19444	-0.19444	-2.44444	-0.69444	-0.64444	2.649785	0.009833	0.313592
	65	0	0.932578	0.941871	0.767216	0.1983836	0.072337	0.767216	0.000388	0.29227	0.292368			
ESS-BS induction Insertions/Total Insertions	66	0.0483333	0.000167	0.000467	-8.3E-05	-0.0006667	-0.00233	-8.3E-05	-0.00383	-0.00083	-0.00053	1.501674	0.162206	0.205662
	66	0	0.911592	0.737767	0.955726	0.57173835	0.123398	0.955726	0.012617	0.579211	0.702022			
Splicing Change Insertions:	67	254.111111	-47.3611	-27.5111	-11.6111	-52.444444	-41.6111	-31.6111	-42.1111	-31.6111	-68.1111	7.750607	1E-07	0.571975
	67	0	0.000215	0.016919	0.340684	5.8074E-07	0.001008	0.011082	0.000885	0.011082	6.84E-08			
Splicing Change Insertions/Total Insertions	68	0.3062222	-0.02322	-0.01082	2.78E-05	-0.0308889	-0.02097	-0.02047	-0.02447	-0.02122	-0.03842	5.040843	2.97E-05	0.464986
	68	0	0.013232	0.205703	0.99758	5.3674E-05	0.024657	0.028158	0.009203	0.023056	2.43E-05			
Protein motif disrupting Insertions:	69	42.2222222	-1.22222	0.177778	0.527778	-7.1111111	-4.97222	-2.72222	-4.97222	4.027778	-2.02222	2.383086	0.019227	0.291221
	69	0	0.717327	0.954736	0.875721	0.00885421	0.143813	0.420944	0.143813	0.235093	0.519152			
Protein motif disrupting Insertions/Total Insertions	70	0.6802222	0.019528	-0.00342	0.006028	-0.021	-0.01072	0.001278	-0.00897	0.033278	0.020778	1.249852	0.28014	0.177288
	70	0	0.394387	0.871914	0.79213	0.24410992	0.639404	0.955437	0.694941	0.148748	0.32932			
Conserved Element Deletions:	71	13709.4444	-1056.94	-885.444	-497.444	-1908	-1764.69	-1529.44	-1698.19	-1476.44	-2397.24	33.80796	0	0.853565
	71	0	3.98E-06	2.44E-05	0.020928	0	5E-12	4.93E-10	1.8E-11	1.4E-09	0			
TFBS Deletions:	72	390.555556	-62.5556	-43.3556	-18.0556	-70.111111	-73.5556	-43.8056	-46.5556	-35.0556	-88.7556	10.63284	0	0.647048
	72	0	2.78E-05	0.001289	0.198439	1.5678E-08	1.44E-06	0.002431	0.001334	0.014059	2.49E-09			
TFBS Deletions/Total Deletions	73	0.0061111	0.000389	8.89E-05	0.000389	0	-0.00011	0.000389	0.000639	0.000889	0.000689	4.214989	0.000205	0.420868
	73	0	0.120913	0.700097	0.120913	1	0.655001	0.120913	0.012053	0.000623	0.00381			
miRNA-BS disrupting Deletions:	74	16.1111111	-2.61111	-2.71111	-3.86111	-6.4444444	-5.61111	-8.61111	-8.86111	-5.61111	-6.91111	9.173746	0	0.612655
	74	0	0.091426	0.059676	0.01368	9.9189E-07	0.000467	3.59E-07	1.88E-07	0.000467	6.79E-06			
miRNA-BS disrupting Deletions/Total Deletions	75	0.0222222	-0.00222	-0.00242	-0.00497	-0.0066667	-0.00547	-0.01072	-0.01072	-0.00647	-0.00662	5.796477	5.5E-06	0.499848
	75	0	0.330606	0.253883	0.0318	0.00037385	0.018551	1.21E-05	1.21E-05	0.005735	0.002466			
ESE-BS deletion Deletions:	76	46.4444444	-6.69444	-4.44444	0.055556	-15	-12.6944	-8.94444	-9.44444	-12.1944	-15.0444	13.02337	0	0.691872
	76	0	0.012122	0.069546	0.982991	3.35E-10	6.62E-06	0.000989	0.000535	1.35E-05	3.33E-08			
ESE-BS deletion Deletions/Total Deletions	77	0.0697778	-0.00328	-0.00138	0.003222	-0.0114444	-0.00703	-0.00453	-0.00528	-0.00928	-0.00758	3.053014	0.003565	0.344856
	77	0	0.435698	0.723613	0.443483	0.00085833	0.097354	0.282588	0.211087	0.029833	0.054977			
ESE-BS induction Deletions:	78	50.7777778	-6.27778	-6.37778	-1.52778	-13	-13.0278	-8.77778	-10.7778	-13.5278	-15.9778	8.367679	0	0.590618
	78	0	0.04293	0.027162	0.617166	7.8704E-07	6.03E-05	0.005254	0.000726	3.36E-05	3.44E-07			
ESE-BS induction Deletions/Total Deletions	79	0.0762222	-0.00172	-0.00422	0.001278	-0.0057778	-0.00647	-0.00297	-0.00647	-0.01047	-0.00762	1.311923	0.24596	0.184468
	79	0	0.717021	0.339824	0.787953	0.1242802	0.175929	0.532038	0.175929	0.030292	0.087246			
ESS-BS deletion Deletions:	80	74.6666667	-12.6667	-8.46667	-5.66667	-12.333333	-14.9167	-10.6667	-14.4167	-9.41667	-24.0667	6.201082	2.3E-06	0.51671
	80	0	0.003811	0.03444	0.184386	0.00040814	0.000755	0.013958	0.001097	0.029189	5.12E-08			
ESS-BS deletion Deletions/Total Deletions	81	0.1181111	-0.00511	-0.00171	0.000389	0.0004444	-0.00336	-0.00186	-0.00761	-0.00136	-0.01411	1.452929	0.180942	0.200323
	81	0	0.38086	0.751346	0.946687	0.92239674	0.563795	0.74905	0.193458	0.814991	0.010756			
ESS-BS induction Deletions:	82	32.6666667	-1.41667	-4.46667	-4.91667	-12.111111	-10.4167	-9.66667	-9.41667	-8.16667	-11.4667	13.41069	0	0.698085
	82	0	0.476721	0.017748	0.015515	5.6E-11	1.61E-06	6.8E-06	1.09E-05	0.000104	3.37E-08			
ESS-BS induction Deletions/Total Deletions	83	0.0516667	0.005333	-0.00127	-0.00417	-0.0125556	-0.00892	-0.00967	-0.00892	-0.00792	-0.00827	5.853358	4.9E-06	0.502289
	83	0	0.141491	0.704635	0.249214	3.1505E-05	0.015362	0.00885	0.015362	0.030636	0.015478			

Splicing Change Deletions:	84	314.333333	-16.3333	-9.53333	-15.0833	-34.222222	-30.0833	-27.8333	-28.5833	-36.0833	-37.1333	11.16579	0	0.658136
	84	0	0.013624	0.115776	0.022271	3.934E-09	1.51E-05	5.32E-05	3.52E-05	4.37E-07	3.87E-08			
Splicing Change Deletions/Total Deletions	85	0.421	0.0165	0.0166	-0.005	0.0006667	0.00125	-0.01775	-0.012	-0.012	0.0172	5.073312	2.76E-05	0.466584
	85	0	0.041348	0.027434	0.530627	0.91500258	0.875261	0.028574	0.13504	0.13504	0.022489			
Protein motif disrupting Deletions:	86	49	2.25	-3	-5	-13.777778	-9.75	-9.75	-13.25	-6.75	-14.4	11.30482	0	0.660914
	86	0	0.424651	0.252638	0.078776	2.9882E-08	0.000884	0.000884	1.2E-05	0.018712	5.51E-07			
Protein motif disrupting Deletions/Total Deletions	87	0.7015556	0.012944	-0.00156	-0.03681	0.0028889	-0.01831	-0.02631	-0.06981	-0.02831	-0.00256	1.665501	0.111247	0.223093
	87	0	0.635039	0.950959	0.179722	0.89250151	0.502436	0.336031	0.012355	0.300849	0.91952			
Conserved Element Rearrangements:	88	3536.11111	-249.111	-151.711	-94.8611	-472.55556	-384.861	-350.111	-383.361	-308.861	-602.711	26.07314	0	0.818029
	88	0	9.71E-05	0.008294	0.119052	0	1.72E-08	1.76E-07	1.91E-08	2.56E-06	0			
TFBS Rearrangements:	89	97	-11.75	-9.8	-10.5	-22.666667	-22.75	-12	-18	-14.25	-25.8	9.973291	0	0.63229
	89	0	0.007867	0.016392	0.016969	4.447E-09	1.37E-06	0.006702	8.13E-05	0.001446	1.27E-08			
TFBS Rearrangements/Total Rearrangements	90	0.0038889	-0.00014	-8.9E-05	-0.00039	-0.0005556	-0.00064	-0.00014	-0.00014	-0.00014	-8.9E-05	1.588501	0.13305	0.214996
	90	0	0.623772	0.735081	0.172232	0.01439267	0.026633	0.623772	0.623772	0.623772	0.735081			
miRNA-BS disrupting Rearrangements:	91	1.6666667	-0.16667	-0.46667	0.833333	-0.2222222	-0.66667	-0.16667	-0.16667	0.333333	-0.46667	0.984763	0.466781	0.145143
	91	4.2063E-06	0.781915	0.404767	0.169222	0.63817262	0.270217	0.781915	0.781915	0.580146	0.404767			
miRNA-BS disrupting Rearrangements/Total Rearrangements	92	0.0213333	-0.00033	-0.00553	0.010667	0.0008889	-0.00683	-0.00133	0.000417	0.006167	-0.00193	0.740468	0.683666	0.113213
	92	1.1872E-05	0.967402	0.465747	0.193785	0.88952033	0.403392	0.870163	0.959259	0.450594	0.798488			
ESE-BS deletion Rearrangements:	93	45.6666667	-3.41667	0.333333	-1.91667	-6.6666667	-3.16667	-1.16667	-4.41667	-2.66667	-6.66667	2.88307	0.005465	0.332034
	93	0	0.170779	0.884729	0.440065	0.00099338	0.203823	0.637908	0.078004	0.283733	0.004893			
ESE-BS deletion Rearrangements/Total Rearrangements	94	0.0758889	-0.00064	0.003311	-0.00139	-0.0027778	0.001361	0.003861	-0.00064	0.000111	-0.00169	0.629316	0.782707	0.097882
	94	0	0.877696	0.391464	0.738052	0.39494491	0.7431	0.353866	0.877696	0.978646	0.661385			
ESE-BS induction Rearrangements:	95	48.1111111	-5.36111	-2.11111	-5.61111	-6.6666667	-6.61111	-3.11111	-5.86111	-2.86111	-10.3111	3.122964	0.002992	0.349992
	95	0	0.055233	0.41076	0.045086	0.00289002	0.018895	0.261576	0.036589	0.301506	0.000139			
ESE-BS induction Rearrangements/Total Rearrangements	96	0.0796667	-0.00317	-0.00027	-0.00742	-0.0018889	-0.00442	0.000583	-0.00267	0.000583	-0.00767	1.107062	0.372583	0.16028
	96	0	0.46177	0.946659	0.087588	0.57543171	0.30561	0.891953	0.535191	0.891953	0.057756			
ESS-BS deletion Rearrangements:	97	30.2222222	-1.22222	-0.62222	1.027778	-1	0.777778	0.277778	-0.97222	0.277778	-2.02222	0.583024	0.821162	0.09134
	97	0	0.508186	0.716362	0.577754	0.49019091	0.673411	0.880281	0.598442	0.880281	0.239886			
ESS-BS deletion Rearrangements/Total Rearrangements	98	0.0931111	0.002389	0.002089	0.005889	0.009	0.010639	0.008139	0.004639	0.006389	0.006689	1.234382	0.289217	0.175478
	98	0	0.636093	0.655742	0.245443	0.02561932	0.037984	0.110046	0.359299	0.208035	0.156248			
ESS-BS induction Rearrangements:	99	31.3333333	-4.83333	-0.13333	-4.08333	-5.7777778	-4.08333	-6.08333	-4.58333	-4.08333	-9.33333	4.988203	3.35E-05	0.462376
	99	0	0.019928	0.943708	0.047953	0.00054204	0.047953	0.003772	0.026986	0.047953	5.07E-06			
ESS-BS induction Rearrangements/Total Rearrangements	100	0.0965556	-0.00906	0.004244	-0.01056	-0.0072222	-0.00581	-0.01256	-0.00756	-0.00756	-0.01856	2.508232	0.014042	0.301897
	100	0	0.14907	0.463657	0.093507	0.14251162	0.352756	0.046987	0.227559	0.227559	0.001965			
Splicing Change Rearrangements:	101	36.7777778	-3.02778	-3.17778	0.472222	-4.2222222	-1.77778	1.972222	-4.52778	-3.52778	-5.57778	2.011126	0.048457	0.257469
	101	0	0.246932	0.191081	0.855999	0.04170137	0.495194	0.449428	0.085273	0.178099	0.023498			
Splicing Change Rearrangements/Total Rearrangements	102	0.2311111	0.000389	-0.00691	0.002139	0.0004444	0.011389	0.024889	-0.00486	0.002639	0.008489	0.801726	0.627529	0.121442
	102	0	0.978285	0.602636	0.881008	0.96836888	0.426476	0.084953	0.733789	0.853483	0.522734			
Protein motif disrupting Rearrangements:	103	77.5555556	-13.5556	-6.15556	-3.80556	-5.6666667	-2.80556	-7.05556	-12.3056	-1.55556	-12.7556	2.862971	0.005748	0.330484
	103	0	0.002672	0.131693	0.384305	0.10112673	0.520736	0.109154	0.006108	0.721496	0.002352			
Protein motif disrupting Rearrangements/Total Rearrangements	104	0.532	-0.024	-0.0006	0.01575	0.0253333	0.03775	0.0085	-0.023	0.015	0.0066	2.171647	0.032597	0.272421
	104	0	0.20844	0.972812	0.40752	0.0920753	0.049804	0.654285	0.227804	0.430105	0.707882			
Nonsense SNPs:	105	117.111111	7.138889	3.088889	-4.86111	-12.444444	-11.6111	-10.3611	-12.1111	-12.6111	-5.11111	4.831018	4.81E-05	0.454427
	105	0	0.150484	0.500097	0.325542	0.00190769	0.0209	0.03851	0.016167	0.01242	0.265911			
Nonsense SNPs/Total SNPs	106	0.0031111	0.000139	8.89E-05	-0.00011	-0.0001111	-0.00011	-0.00011	-0.00011	-0.00011	8.89E-05	0.811954	0.618177	0.122801
	106	0	0.408203	0.567975	0.507805	0.39903132	0.507805	0.507805	0.507805	0.507805	0.567975			
Frameshift Structural Variants:	107	450.111111	-7.86111	-3.51111	-15.3611	-64.555556	-36.1111	-35.3611	-37.6111	-21.3611	-55.1111	14.22642	0	0.710383
	107	0	0.438496	0.708816	0.132472	1.2E-11	0.000644	0.000817	0.000398	0.037902	1.39E-07			
Frameshift Structural Variants/Total Variants	108	0.4181111	0.018139	0.012689	0.002139	-0.0093333	0.000389	-0.00186	-0.00511	-0.00036	0.007289	5.060437	2.84E-05	0.465952
	108	0	0.001967	0.017865	0.705209	0.03830174	0.945135	0.741996	0.36721	0.949048	0.167671			
Frameshift Insertions:	109	203.888889	-2.38889	-5.48889	-9.13889	-29.444444	-14.3889	-10.6389	-14.3889	-9.38889	-16.8889	6.848707	6E-07	0.541455
	109	0	0.688913	0.323174	0.128722	2.4705E-08	0.018158	0.077873	0.018158	0.118765	0.003159			
Frameshift Insertions/Total Insertions	110	0.5913605	0.033013	0.016608	0.010811	-0.0077879	0.003888	0.014475	0.006443	0.007257	0.026904	5.025395	3.08E-05	0.464223
	110	0	0.000349	0.045054	0.221487	0.2611698	0.658586	0.103157	0.464675	0.410404	0.001511			
Frameshift Deletions:	111	223.333333	-2.83333	-0.93333	-8.83333	-33	-24.5833	-28.0833	-22.3333	-12.8333	-35.3333	15.44902	0	0.727046
	111	0	0.616964	0.858998	0.121915	2.26E-10	4.59E-05	4.71E-06	0.000183	0.026046	4.21E-09			
Frameshift Deletions/Total Deletions	112	0.7552032	0.009756	0.013147	-0.00511	0.0055582	0.020701	-0.0041	-0.00818	-0.00512	0.02035	1.84944	0.071823	0.241775
	112	0	0.38709	0.210615	0.650053	0.52934574	0.069109	0.715527	0.467718	0.64928	0.05457			
Frameshift Rearrangements:	113	22.8888889	-2.63889	2.911111	2.611111	-2.1111111	2.861111	3.361111	-0.88889	0.861111	-2.88889	3.040397	0.00368	0.343921
	113	0	0.208067	0.135547	0.212837	0.19930757	0.172725	0.110133	0.669893	0.679614	0.138507			
Frameshift Rearrangements/Total Rearrangements	114	0.0523956	-0.00201	0.00888	0.007675	0.000241	0.009685	0.010442	0.000863	0.004003	-0.00024	2.330316	0.021945	0.286621
	114	0	0.66288	0.040741	0.098828	0.94678471	0.038397	0.025969	0.85125	0.385762	0.954858			
Splicing Change Variants:	115	3009.33333	-17.0833	17.46667	9.416667	4.3333333	14.91667	-34.0833	-48.5833	-5.08333	2.066667	2.379966	0.019378	0.290951
	115	0	0.377657	0.331449	0.626055	0.77486564	0.440788	0.080959	0.013918	0.792385	0.908195			
Splicing Change Variants/Total Variants	116	0.3357778	0.006722	0.006022	0.005222	0.0248889	0.023972	0.019222	0.020222	0.018472	0.026422	69.99561	0	0.923478
	116	0	0.000817	0.001199	0.008231	0	0	0	0	0	0			
Probably Damaging nscSNPs:	117	2842.66667	15.83333	9.133333	-42.9167	-197.88889	-168.917	-198.667	-213.167	-177.417	-208.667	53.20629	0	0.901705
	117	0	0.460169	0.645822	0.04808	0	3.3E-11	0	0	6E-12	0			
Probably Damaging nscSNPs/Total nscSNPs	118	0.1638889	0.000861	0.000111	-0.00189	-0.0045556	-0.00364	-0.00289	-0.00389	-0.00439	-0.00469	8.87044	0	0.604647
	118	0	0.448213	0.915858	0.098909	2.5279E-06	0.001956	0.012751	0.000989	0.000234	3.03E-05			
Possibly Damaging nscSNPs:	119	2201.44444	17.30556	11.55556	-13.1944	-148	-120.944	-150.194	-139.694	-143.194	-133.844	27.15811	0	0.824019
	119	0	0.43806	0.576517	0.553978	3E-12	7.73E-07	3.89E-09	2.68E-08	1.41E-08	1.17E-08			
Possibly Damaging nscSNPs/Total nscSNPs	120	0.127	0.001	0.0002	-0.0005	-0.0032222	-0.00225	-0.00225	-0.0015	-0.004	-0.0022	4.90466	4.06E-05	0.45818
	120	0	0.373101	0.847379	0.655355	0.00046204	0.047649	0.047649	0.183155	0.000631	0.037252			
Protein motif damaging Variants:	121	1012.22222	-16.7222	-21.0222	-17.7222	-111.44444	-82.2222	-89.4722	-109.722	-69.4722	-108.222	18.20521	0	0.758386
	121	0	0.329232	0.187657	0.301344	6E-12	8.2E-06	1.63E-06	1.45E-08	0.000121	2.78E-09			
Protein motif damaging Variants/Total Variants	122	0.2003333	-0.00283	-0.00393	-0.00158	-0.0107778	-0.00733	-0.00633	-0.01083	-0.00608	-0.01013	6.279203	0.000002	0.519836
	122	0	0.280784	0.108589	0.545487	1.5489E-06	0.006412	0.017761	9.34E-05	0.022571	8.35E-05			
TFBS Disrupting Variants:	123	625.666667	-92.6667	-65.6667	-39.4167	-122.55556	-117.667	-74.1667	-80.6667	-56.4167	-155.667	17.4075	0	0.750081
	123	0	3.95E-06	0.000277	0.036134	3E-12	1.89E-08	0.000148	4.33E-05	0.003176	0			
TFBS Disrupting Variants/Total Variants	124	0.001	0	0	0	0	0	0	0	0	0	0.599519	0.807727	0.093682
	124	0	1	1	1	0.10989014	1	1	1	1	1			
miRNA-BS Disrupting Variants:	125	215.777778	-7.27778	-10.7778	-15.7778	-51	-42.5278	-49.7778	-51.7778	-41.0278	-66.5778	56.81906	0	0.907376
	125	0	0.145237	0.021656	0.002133	0	2E-12	0	0	7E-12	0			
miRNA-BS Disrupting Variants/Total Variants	126	0.029	-0.001	-0.0016	-0.00225	-0.005	-0.00375	-0.0045	-0.005	-0.00425	-0.0066	19.43587	0	0.770168
	126	0	0.207037	0.03154	0.005539	1.5E-11	1.01E-05	2.56E-07	1.99E-08	8.94E-07	0			
ESE-BS Disrupting Variants:	127	2912	-33.25	-6.8	-35.75	-141.88889	-138.5	-187.75	-163.25	-134.25	-140	25.7705	0	0.816284
	127	0	0.131931	0.737891	0.105731	7E-12	2.14E-08	2E-12	2.01E-10	4.71E-08	2.12E-09			
ESE-BS Disrupting Variants/Total Variants	128	0.1113333	-0.00083	6.67E-05	-0.00108	0.0004444	-8.3E-05	-0.00058	0.000167	-0.00033	0.000867	1.3104	0.246756	0.184294
	128	0	0.271876	0.924213	0.154456	0.45396129	0.912116	0.440765	0.825318	0.659084	0.218812			
ESS-BS Disrupting Variants:	129	1225.55556	-17.3056	-1.35556	15.19444	-43.555556	-40.3056	-54.0556	-71.5556	-40.3056	-84.5556	15.07624	0	0.722172
	129	0	0.171766	0.907548	0.229456	3.5477E-05	0.001995	5.38E-05	2.79E-07	0.001995	4.94E-10			
ESS-BS Disrupting Variants/Total Variants	130	0.1185556	-0.00106	0.000244	0.001694	0.0013333	0.001444	0.000944	-0.00056	0.000694	-0.00236	3.033093	0.003748	0.343378
	130	0	0.332875	0.808474	0.122116	0.12098843	0.186477	0.385933	0.609385	0.523254	0.021995			
**Total Likely Functional Variants:**	131	12983.1111	-148.111	-71.3111	-133.111	-730.11111	-599.611	-729.611	-734.861	-557.861	-783.111	52.73723	0	0.900918
	131	0	0.040634	0.282731	0.06498	0	4E-12	0	0	4.2E-11	0			
synonymous to nonsynonymous rate	132	1.060125	-0.00319	-0.00397	-0.00041	-0.011464	-0.01301	-0.00988	-0.00697	-0.00717	-0.00912	3.596875	0.000921	0.382774
	132	0	0.411924	0.272318	0.91552	0.00033459	0.001265	0.012868	0.075948	0.067974	0.01334			
Likely functional variant rate	133	0.0021487	5.3E-06	8.6E-06	-3.8E-06	0.0000212	3.65E-05	0.000034	3.86E-05	2.75E-05	3.09E-05	7.693311	1E-07	0.570157
	133	0	0.539406	0.280871	0.655044	0.00233086	6.34E-05	0.000175	2.7E-05	0.001986	0.00023			
Likely functional variant rate (minus probdam)	134	0.0017843	-2.7E-06	3.2E-06	-4.7E-06	0.0000219	3.37E-05	0.000033	3.47E-05	3.14E-05	2.59E-05	10.21933	0	0.637938
	134	0	0.721984	0.648638	0.533624	0.00046007	3.39E-05	4.85E-05	2.11E-05	0.000101	0.000477			
Functional Nonsense SNPs/Total Variants	135	0.0000194	1.5E-06	7E-07	-6E-07	-0.0000008	-8E-07	-4E-07	-6E-07	-1.1E-06	6E-07	2.173873	0.032418	0.272624
	135	0	0.073779	0.363646	0.429501	0.1889537	0.349463	0.652777	0.437451	0.187383	0.406152			
Functional Frameshift Structural Variants/Total Variants	136	0.0000745	-3E-07	1E-07	-1.9E-06	-0.0000062	-1.4E-06	-6E-07	-8E-07	6E-07	-3.9E-06	5.330345	1.54E-05	0.478902
	136	0	0.868332	0.93464	0.245894	8.3642E-06	0.385067	0.711976	0.61519	0.717649	0.012294			
Functional Insertions/Total Variants	137	0.0000337	1E-07	-6E-07	-1.2E-06	-0.0000028	-3E-07	7E-07	1E-07	3E-07	-3E-07	3.29969	0.001924	0.362616
	137	0	0.938914	0.522273	0.218408	0.00048758	0.764442	0.491051	0.918088	0.743828	0.720749			
Functional Deletions/Total Variants	138	0.000037	0	2E-07	-1.2E-06	-0.0000033	-1.9E-06	-2.2E-06	-1.1E-06	-1E-07	-3.4E-06	5.564927	9.2E-06	0.489658
	138	0	0.965838	0.823528	0.22471	4.1724E-05	0.049597	0.024191	0.260861	0.916428	0.000275			
Functional Rearrangements/Total Variants	139	0.0000038	-4E-07	5E-07	5E-07	-0.0000001	8E-07	9E-07	1E-07	4E-07	-2E-07	3.008465	0.003987	0.341543
	139	0	0.273374	0.117365	0.188365	0.69585363	0.035924	0.014214	0.688157	0.297612	0.514203			
Functional Splicing Change Variants/Total Variants	140	0.0004981	4.1E-06	7.7E-06	5.8E-06	0.0000356	3.56E-05	3.19E-05	3.06E-05	2.81E-05	3.99E-05	61.24577	0	0.913492
	140	0	0.205905	0.01186	0.074427	0	0	0	0	1E-12	0			
Probably Damaging nscSNPs/Total Variants	141	0.0004705	9.2E-06	0.000006	-3.2E-06	-0.0000021	1.3E-06	5E-07	-9E-07	-3.7E-06	1E-07	2.392361	0.018785	0.292023
	141	0	0.014841	0.084187	0.395701	0.47411732	0.716406	0.893574	0.807771	0.322633	0.977038			
Possibly Damaging nscSNPs/Total Variants	142	0.0003643	0.000008	5.4E-06	9E-07	-0.0000007	2.8E-06	0.000001	3.8E-06	-3.8E-06	0.000005	2.084081	0.040492	0.26434
	142	0	0.025249	0.099706	0.795271	0.80139425	0.42755	0.765843	0.275709	0.274837	0.12397			
Functional Protein motif damaging Variants/Total Variants	143	0.0001675	-4E-07	-1.9E-06	-1.5E-06	-0.000008	-3.4E-06	-3.1E-06	-6.3E-06	-2.4E-06	-6E-06	2.995991	0.004114	0.340609
	143	0	0.869618	0.44773	0.575	0.00029506	0.207409	0.243589	0.020196	0.371764	0.017927			
Functional TFBS Disrupting Variants/Total Variants	144	0.0001035	-1.4E-05	-0.00001	-5.7E-06	-0.0000144	-1.4E-05	-5.3E-06	-6.2E-06	-3.8E-06	-2E-05	11.5318	0	0.665355
	144	0	9.62E-06	0.000484	0.058101	3.0267E-08	1.21E-05	0.076607	0.038901	0.198774	7.35E-10			
Functional miRNA-BS Disrupting Variants/Total Variants	145	0.0000357	-7E-07	-1.5E-06	-2.3E-06	-0.0000065	-5.1E-06	-6.1E-06	-6.4E-06	-5.1E-06	-9.1E-06	33.16858	0	0.851162
	145	0	0.392091	0.066179	0.007054	0	5.15E-08	3.86E-10	9.5E-11	6.08E-08	0			
Functional ESE-BS Disrupting Variants/Total Variants	146	0.0004819	1.2E-06	3.5E-06	-1.8E-06	0.0000086	7.5E-06	3.4E-06	0.000009	4.6E-06	1.33E-05	5.333635	1.53E-05	0.479056
	146	0	0.703699	0.23166	0.559821	0.00077108	0.019247	0.287623	0.005595	0.146141	2E-05			
Functional ESS-BS Disrupting Variants/Total Variants	147	0.0002028	0	1.7E-06	4.3E-06	0.0000065	6.3E-06	5.9E-06	3.3E-06	4.8E-06	0.000001	3.947317	0.000391	0.404964
	147	0	0.985652	0.377568	0.045519	0.00018706	0.003646	0.006862	0.126088	0.026337	0.599922			
