# Supplementary material for: Clinical Implications of Human Population Differences in Genome-Wide Rates of Functional Genotypes
Source: Front Genet. 2012 Nov 1;3:211. doi: 10.3389/fgene.2012.00211 (PMC3485509; doi:10.3389/fgene.2012.00211)
Supplement: Supplementary Data Sheet S4 — Regression analysis results for ancestral allele-based variants: all homozygous variants. [file 32001_Schork_DataSheet4.PDF]

Ancestral Allele Based Variants: All Homozygous Variants
Row 1: Regression Coefficients
Row 2: P-values for Regression Coefficients

	Var Cat	Y-int	LWK	ASW	MKK	CEU	TSI	CHB	JPT	GIH	MEX	Overall F	p-val	R-Sqr
**Coding SNPs:**	1	18736.5556	159.6944	64.44444	264.4444	2335.33333	2174.694	2532.694	2526.944	1857.194	2460.444	329.4631	0	0.9827
	1	0	0.111535	0.485667	0.0095	0	0	0	0	0	0			
Nonsynonymous SNPs:	2	8833.44444	62.55556	52.75556	118.8056	1160.66667	1060.556	1236.806	1205.056	878.8056	1219.356	287.5783	0	0.98023
	2	0	0.232209	0.277265	0.025196	0	0	0	0	0	0			
Synonymous SNPs:	3	9872.22222	97.52778	14.17778	146.0278	1171.11111	1110.028	1284.528	1314.278	975.2778	1236.578	282.0506	0	0.979851
	3	0	0.077448	0.779688	0.009133	0	0	0	0	0	0			
Nonsense SNPs:	4	30.8888889	-0.38889	-2.48889	-0.38889	3.5555556	4.111111	11.36111	7.611111	3.111111	4.511111	7.720475	1E-07	0.571021
	4	0	0.855895	0.213116	0.855895	0.03730183	0.058195	1.26E-06	0.00067	0.149395	0.025902			
Untranslated Region SNPs:	5	24831.7778	138.7222	87.82222	431.7222	3502.55556	3350.222	4007.972	3956.972	3009.722	3642.222	510.9297	0	0.988776
	5	0	0.261817	0.442918	0.000777	0	0	0	0	0	0			
Non-coding RNA SNPs:	6	777642.889	8898.861	7412.111	9095.111	101894.889	100721.4	111561.6	114216.1	92072.61	107299.9	585.2983	0	0.990188
	6	0	0.008621	0.017847	0.007325	0	0	0	0	0	0			
Intronic SNPs:	7	1085836.56	7635.944	4793.644	14298.44	151122.778	145273.7	166210.7	167703.9	131574.9	153659.8	962.3611	0	0.994009
	7	0	0.04891	0.179445	0.000364	0	0	0	0	0	0			
Intergenic SNPs:	8	1492797.22	9986.028	10095.58	18026.78	205015.444	200367.5	228947.5	228514.3	181023.3	215134.8	761.9715	0	0.992446
	8	0	0.093042	0.067879	0.003038	0	0	0	0	0	0			
**Total SNPs:**	9	2628338.89	17996.11	15119.71	33044.61	362700.333	351891.1	402459.1	403471.1	318042.9	375597.7	928.5199	0	0.993792
	9	0	0.058561	0.086126	0.000748	0	0	0	0	0	0			
**Coding Insertions:**	10	175.111111	-0.36111	-7.31111	2.138889	2.3333333	5.138889	6.138889	9.138889	6.888889	-1.51111	3.65978	0.000789	0.386878
	10	0	0.920752	0.032912	0.556182	0.4136812	0.159922	0.094214	0.013861	0.061065	0.65401			
In-frame Insertions:	11	49.5555556	1.694444	0.444444	0.444444	1.7777778	1.194444	0.944444	4.944444	0.194444	2.844444	2.257593	0.026318	0.280182
	11	0	0.245885	0.741819	0.759752	0.12213284	0.412178	0.516314	0.001084	0.893539	0.037959			
Out-of-frame Insertions:	12	20.8888889	-3.13889	-2.28889	0.361111	0.1111111	4.611111	-0.38889	1.611111	0.111111	-2.28889	2.287788	0.024407	0.282869
	12	0	0.124609	0.226066	0.858542	0.94426168	0.025507	0.847792	0.427548	0.956261	0.226066			
Frameshift Insertions:	13	104.666667	1.083333	-5.46667	1.333333	0.4444444	-0.66667	5.583333	2.583333	6.583333	-2.06667	3.389196	0.00154	0.368824
	13	0	0.688129	0.031882	0.621397	0.83365793	0.804827	0.041571	0.339842	0.016911	0.410293			
Untranslated region Insertions:	14	1882.22222	-65.2222	-57.4222	-10.9722	43.7777778	43.02778	113.5278	94.77778	38.52778	4.177778	12.83838	0	0.688814
	14	0	0.005592	0.008401	0.631603	0.01691008	0.063232	4.65E-06	9.24E-05	0.095414	0.843962			
Non-coding RNA Insertions:	15	42957.4444	-1670.94	-1510.64	-407.694	568	974.8056	2520.056	2167.556	1260.806	-261.044	17.06367	0	0.746322
	15	0	0.000728	0.000974	0.39053	0.12951422	0.042654	1.18E-06	1.97E-05	0.009439	0.553056			
Intronic Insertions:	16	65001.5556	-2288.31	-2478.56	-517.056	790.333333	1221.444	3573.444	3086.444	1557.944	-294.556	19.25178	0	0.76848
	16	0	0.000608	8.08E-05	0.41905	0.11783689	0.05902	4.02E-07	7.59E-06	0.016909	0.619392			
Intergenic Insertions:	17	82923	-3543	-3132	-614.5	977.777778	1369.25	5229	4294.5	2122.75	-603.6	17.35069	0	0.749468
	17	0	0.000348	0.000624	0.515437	0.18925258	0.149797	4.99E-07	2.16E-05	0.02716	0.491356			
**Total Insertions:**	18	150237.778	-5909.53	-5691.78	-1149.53	1809.66667	2641.722	8922.472	7486.722	3730.722	-906.178	18.32001	0	0.759536
	18	0	0.000406	0.000255	0.471497	0.15082977	0.100752	4E-07	1.26E-05	0.021718	0.540631			
**Coding Deletions:**	19	93.5555556	-1.30556	-3.55556	0.944444	0.2222222	5.444444	8.694444	11.94444	9.444444	-0.15556	7.206823	3E-07	0.55408
	19	0	0.641552	0.174599	0.736195	0.91948003	0.055338	0.002712	6.12E-05	0.001201	0.95231			
In-frame Deletions:	20	7.2222222	-2.47222	-3.22222	-1.72222	-1.8888889	0.527778	-0.72222	0.777778	1.027778	-1.62222	3.63868	0.000831	0.385507
	20	0	0.031334	0.002933	0.130259	0.03585234	0.640273	0.522803	0.491441	0.363895	0.12476			
Inter-Codon Deletions:	21	6.2222222	-0.97222	0.177778	-0.47222	1.3333333	-0.22222	3.777778	0.277778	0.527778	-1.02222	2.213739	0.029358	0.276243
	21	3E-12	0.464388	0.885173	0.721904	0.20271466	0.866936	0.005654	0.834115	0.690822	0.407493			
Frameshift Deletions:	22	80.1111111	2.138889	-0.51111	3.138889	0.7777778	5.138889	5.638889	10.88889	7.888889	2.488889	3.6337	0.000841	0.385183
	22	0	0.451862	0.846124	0.27074	0.72685263	0.073515	0.05011	0.000264	0.006837	0.346189			
Untranslated region Deletions:	23	1251.11111	-59.6111	-55.7111	-6.86111	56.4444444	63.88889	135.1389	123.6389	64.63889	38.68889	26.33368	0	0.819504
	23	0	0.00179	0.001673	0.709281	0.00020613	0.000869	3.22E-10	4.29E-09	0.000763	0.026137			
Non-coding RNA Deletions:	24	30579	-2044.75	-1739.4	-379	1668.66667	2044.25	4085	3587.5	2224	791.6	29.46792	0	0.835545
	24	0	0.000307	0.00086	0.48277	0.00018328	0.000308	1.23E-10	5.62E-09	9.87E-05	0.116954			
Intronic Deletions:	25	46147.7778	-3079.53	-2968.38	-705.528	2183.88889	2390.222	5334.722	4832.472	2836.222	709.6222	27.21798	0	0.824338
	25	0	0.000199	0.000119	0.370311	0.00068981	0.003222	3.18E-09	4.34E-08	0.000556	0.3319			
Intergenic Deletions:	26	58689.5556	-4242.06	-3857.16	-629.306	3204.55556	3600.944	8071.444	6916.694	4120.194	1320.244	30.11684	0	0.838516
	26	0	0.000152	0.0002	0.553253	0.00025076	0.001105	1.06E-10	9.61E-09	0.000224	0.18256			
**Total Deletions:**	27	106337.556	-7390.31	-6889.96	-1349.06	5450.66667	6068.944	13565.44	11899.19	7042.444	2072.044	29.3054	0	0.834783
	27	0	0.000157	0.000148	0.46712	0.00035124	0.001599	3.51E-10	1.44E-08	0.000297	0.230459			
**Coding rearrangements:**	28	189.444444	1.305556	0.155556	2.305556	8	2.305556	12.30556	20.55556	14.05556	10.35556	8.618743	0	0.597746
	28	0	0.707727	0.961596	0.508396	0.00448739	0.508396	0.000713	1.18E-07	0.000134	0.001993			
In-frame rearrangements:	29	183.888889	3.361111	0.511111	1.861111	7.5555556	2.361111	11.36111	20.61111	13.86111	11.11111	9.038383	0	0.609122
	29	0	0.312563	0.868107	0.575027	0.00482154	0.477221	0.001007	3.38E-08	8.16E-05	0.00056			
Frameshift rearrangements:	30	5.5555556	-2.05556	-0.35556	0.444444	0.4444444	-0.05556	0.944444	-0.05556	0.194444	-0.75556	1.691132	0.104749	0.225751
	30	0	0.022864	0.66562	0.616163	0.52304864	0.949989	0.28835	0.949989	0.826273	0.359612			
Untranslated region rearrangements:	31	295.888889	-8.13889	-0.08889	-9.13889	8.4444444	19.86111	34.11111	26.86111	14.11111	7.511111	9.130208	0	0.611526
	31	0	0.222072	0.988473	0.170974	0.10777085	0.003706	2.34E-06	0.000127	0.036262	0.224706			
Non-coding RNA rearrangements:	32	10916.6667	-470.417	-446.867	-95.9167	537.888889	762.3333	1272.583	1170.833	733.8333	338.3333	34.1935	0	0.854976
	32	0	0.002559	0.002055	0.525003	2.1706E-05	3.25E-06	3E-12	5.5E-11	6.65E-06	0.017858			
Intronic rearrangements:	33	13727.2222	-815.722	-823.022	-227.472	706.555556	887.0278	1628.028	1641.278	838.5278	360.1778	35.19455	0	0.858518
	33	0	0.000239	7.51E-05	0.282843	5.9414E-05	7.47E-05	6.8E-11	5.3E-11	0.000166	0.06918			
Intergenic rearrangements:	34	20225.2222	-1123.47	-1030.82	-261.472	1132.55556	1357.528	2793.278	2397.778	1405.278	659.7778	39.69538	0	0.872515
	34	0	0.000351	0.000404	0.38374	7.9426E-06	2.3E-05	0	2E-11	1.28E-05	0.019988			
**Total rearrangements:**	35	34537.2222	-1948.22	-1857.82	-495.472	1860.77778	2280.278	4482.778	4096.028	2283.278	1043.778	40.14189	0	0.873754
	35	0	0.000219	0.000152	0.323791	1.0784E-05	2.12E-05	0	1E-11	2.07E-05	0.027338			
**Total number of variants:**	36	***2919451.4***	2748.056	680.1556	**30050.56**	***371821.44***	***362882***	***429430***	***426953***	***331099***	***377807***	983.2094	0	***0.99414***
	36	0	0.775661	0.93941	0.002598	0	0	0	0	0	0			
Conserved Element SNPs:	37	105679.778	505.9722	-93.9778	843.2222	15593.4444	15155.47	18430.47	18164.72	13628.22	16245.22	838.7475	0	0.993132
	37	0	0.255408	0.819142	0.060188	0	0	0	0	0	0			
TFBS SNPs:	38	4.5555556	-0.05556	1.444444	2.444444	0.6666667	1.944444	5.194444	4.194444	3.694444	1.844444	8.195732	0	0.585588
	38	1E-12	0.952217	0.096678	0.010128	0.36083469	0.039031	3.95E-07	2.39E-05	0.000161	0.035056			
TFBS SNPs/Total SNPs	39	0	0	0	0	0	0	0	0	0	0	0	1	0
	39													
miRNA-BS disrupting SNPs:	40	36.6666667	-4.16667	-2.86667	-0.16667	6.1111111	4.333333	15.33333	13.83333	7.083333	4.333333	12.44751	0	0.682149
	40	0	0.114607	0.240222	0.949204	0.00391182	0.101067	1.42E-07	1.35E-06	0.00836	0.077776			
miRNA-BS disrupting SNPs/Total SNPs	41	0.0146667	-0.00142	-0.00067	-0.00017	0.0001111	-0.00042	0.003083	0.002583	0.000833	-0.00047	3.90588	0.000432	0.402424
	41	0	0.15594	0.469433	0.866445	0.88634811	0.674334	0.002642	0.010961	0.401609	0.612233			
ESE-BS deletion SNPs:	42	578.333333	5.916667	4.866667	9.666667	96.5555556	93.41667	86.16667	92.66667	65.41667	105.6667	47.6956	0	0.89158
	42	0	0.568439	0.613165	0.352395	0	0	6E-12	0	2.28E-08	0			
ESE-BS deletion SNPs/Total SNPs	43	0.0456667	-0.00017	0.000133	8.33E-05	0.0015556	0.001583	0.000333	0.001083	0.000333	0.002133	3.695214	0.000723	0.389166
	43	0	0.800911	0.827932	0.899648	0.00365741	0.018936	0.614279	0.104528	0.614279	0.000856			
ESE-BS induction SNPs:	44	772.888889	-1.63889	-7.08889	-4.13889	72.3333333	67.36111	83.86111	86.11111	64.86111	78.91111	73.84444	0	0.927176
	44	0	0.822706	0.298286	0.571878	0	0	0	0	1E-12	0			
ESE-BS induction SNPs/Total SNPs	45	0.0608889	-0.00089	-0.00109	-0.00114	-0.0016667	-0.00189	-0.00139	-0.00114	-0.00114	-0.00149	2.698294	0.008702	0.31751
	45	0	0.123142	0.043219	0.049497	0.00039381	0.001468	0.017354	0.049497	0.049497	0.006352			
ESS-BS deletion SNPs:	46	232.333333	-0.58333	-1.53333	9.416667	40.7777778	43.16667	48.16667	44.41667	30.16667	43.26667	54.41906	0	0.903685
	46	0	0.896345	0.712292	0.038499	0	0	0	0	4.01E-09	0			
ESS-BS deletion SNPs/Total SNPs	47	0.0521111	-0.00036	-0.00031	0.001389	0.0011111	0.002139	0.001889	0.001389	0.000639	0.001889	2.674815	0.009232	0.315619
	47	0	0.671326	0.693659	0.105851	0.09924707	0.013964	0.029146	0.105851	0.453461	0.019092			
ESS-BS induction SNPs:	48	245	8	3	14	37.7777778	31.5	46.75	51	33.75	40.2	38.23071	0	0.868274
	48	0	0.097583	0.4996	0.004497	0	7.34E-09	0	0	1.05E-09	0			
ESS-BS induction SNPs/Total SNPs	49	0.0553333	0.001167	0.000267	0.001917	0	-0.00083	0.000917	0.001917	0.000667	0.000467	1.665991	0.111119	0.223144
	49	0	0.226502	0.76466	0.048969	1	0.386386	0.340971	0.048969	0.487899	0.600605			
Splicing Change SNPs:	50	1756.33333	44.16667	31.46667	17.66667	198.444444	203.4167	170.4167	180.6667	141.6667	218.4667	58.1507	0	0.909305
	50	0	0.018695	0.068721	0.338468	0	0	0	0	7.4E-11	0			
Splicing Change SNPs/Total SNPs	51	0.4757778	0.008972	0.008222	0.000722	-0.009	-0.00753	-0.02028	-0.01753	-0.01053	-0.00898	22.88625	0	0.797813
	51	0	0.003168	0.003547	0.806095	0.00021439	0.012443	2.11E-09	9.58E-08	0.00062	0.001549			
Protein Motif disrupting SNPs:	52	265.333333	-1.83333	-2.53333	5.166667	45.4444444	39.16667	67.91667	57.41667	27.41667	58.66667	35.95544	0	0.861096
	52	0	0.793641	0.697049	0.4618	7E-12	4.16E-07	0	9E-12	0.000205	0			
Protein Motif disrupting SNPs/Total SNPs	53	0.1202222	-0.00122	-2.2E-05	2.78E-05	0.0034444	0.003278	0.011278	0.008528	-0.00022	0.008378	6.540439	1.1E-06	0.530001
	53	0	0.626074	0.992378	0.991156	0.08321362	0.19373	2.61E-05	0.001086	0.929345	0.000576			
Conserved Element Insertions:	54	6957.22222	-236.972	-227.622	-36.9722	78.6666667	88.27778	388.2778	362.2778	136.0278	-16.8222	21.64876	0	0.788697
	54	0	0.000304	0.000194	0.554136	0.11152005	0.160351	3.32E-08	1.78E-07	0.032198	0.771604			
TFBS Insertions:	55	22.3333333	-4.33333	-3.53333	-1.58333	-5.1111111	-3.33333	-1.33333	1.666667	-0.83333	-4.93333	3.406313	0.001476	0.369998
	55	0	0.029124	0.054312	0.418135	0.00132041	0.090941	0.495045	0.394187	0.669455	0.00798			
TFBS Insertions/Total Insertions	56	0.002	-0.00025	0	-0.00025	-0.0005556	0	-0.00025	0	0	-0.0002	2.387323	0.019024	0.291588
	56	0	0.244466	1	0.244466	0.00143123	1	0.244466	1	1	0.315157			
miRNA-BS disrupting Insertions:	57	5.3333333	0.166667	0.266667	0.166667	-0.5555556	-0.08333	0.666667	0.666667	0.166667	-0.53333	1.939863	0.057684	0.250633
	57	0	0.721983	0.540031	0.721983	0.13364991	0.858748	0.157582	0.157582	0.721983	0.222311			
miRNA-BS disrupting Insertions/Total Insertions	58	0.0115556	0.000194	0.000644	-5.6E-05	-0.0017778	-0.00081	0.000444	0.000444	-0.00031	-0.00156	2.094641	0.039449	0.265324
	58	0	0.840571	0.473365	0.95416	0.02153137	0.405774	0.645871	0.645871	0.75197	0.086353			
ESE-BS deletion Insertions:	59	12.4444444	0.055556	0.155556	-0.44444	-0.5555556	-0.19444	-1.94444	-1.44444	0.305556	-0.44444	2.930072	0.004855	0.33563
	59	0	0.924632	0.775417	0.450122	0.23034925	0.740661	0.001444	0.01611	0.603207	0.416012			
ESE-BS deletion Insertions/Total Insertions	60	0.0382222	-0.00097	0.000178	-0.00147	-0.0034444	-0.00247	-0.00772	-0.00622	-0.00122	-0.00402	4.615437	7.95E-05	0.443134
	60	0	0.588685	0.915074	0.413539	0.01673513	0.171658	5.36E-05	0.000894	0.496911	0.018154			
ESE-BS induction Insertions:	61	24.8888889	0.611111	-0.08889	0.361111	2.1111111	3.611111	1.861111	2.111111	4.111111	3.311111	6.329476	1.8E-06	0.521826
	61	0	0.507832	0.917216	0.695258	0.00460524	0.000201	0.046578	0.02457	3E-05	0.000236			
ESE-BS induction Insertions/Total Insertions	62	0.0766667	-0.00042	-0.00107	0.000583	0.002	0.006083	0.001083	0.001833	0.007583	0.003933	2.612591	0.010798	0.310557
	62	0	0.868155	0.647289	0.816242	0.31156476	0.017652	0.666213	0.465983	0.003444	0.094759			
ESS-BS deletion Insertions:	63	45.2222222	4.777778	1.577778	0.027778	4.2222222	2.527778	1.027778	2.527778	4.277778	3.777778	2.816134	0.006467	0.326844
	63	0	0.006216	0.318402	0.986943	0.00221156	0.13965	0.545378	0.13965	0.013773	0.018852			
ESS-BS deletion Insertions/Total Insertions	64	0.1311111	0.005639	0.002889	-0.00261	0.0023333	-0.00236	-0.00411	0.001889	0.005389	-0.00031	1.02928	0.430987	0.150716
	64	0	0.215316	0.4923	0.564369	0.51161902	0.602158	0.36504	0.676537	0.236123	0.940949			
ESS-BS induction Insertions:	65	17.6666667	1.333333	-0.66667	1.333333	0.3333333	-0.16667	1.083333	-1.66667	-0.66667	0.333333	2.418553	0.01759	0.29428
	65	0	0.120831	0.400334	0.120831	0.61819116	0.844885	0.206131	0.053671	0.434851	0.673499			
ESS-BS induction Insertions/Total Insertions	66	0.0511111	0.000639	-0.00251	0.002889	-0.0025556	-0.00386	0.000139	-0.00661	-0.00436	-0.00311	2.815549	0.006477	0.326799
	66	0	0.785252	0.250788	0.22039	0.1676321	0.102938	0.952752	0.006122	0.066213	0.155864			
Splicing Change Insertions:	67	83.1111111	-2.86111	-3.31111	-1.86111	-2	1.138889	3.138889	0.388889	2.888889	-1.51111	0.57786	0.825301	0.090604
	67	0	0.50303	0.404139	0.662784	0.55050917	0.789492	0.462643	0.927348	0.498907	0.702816			
Splicing Change Insertions/Total Insertions	68	0.2025556	-0.00231	-0.00096	-0.00181	-0.0098889	-0.00406	-0.00231	-0.00681	-0.00406	-0.00816	0.483225	0.894212	0.076907
	68	0	0.780798	0.901088	0.827465	0.13130493	0.624696	0.780798	0.412447	0.624696	0.290789			
Protein motif disrupting Insertions:	69	12.7777778	-1.27778	0.022222	-1.27778	-1	-1.77778	0.222222	-0.02778	1.222222	-1.57778	0.989176	0.46316	0.145699
	69	0	0.35777	0.986209	0.35777	0.35890096	0.202057	0.872544	0.983999	0.378926	0.222305			
Protein motif disrupting Insertions/Total Insertions	70	0.5366667	-0.02467	0.006733	-0.03292	-0.0396667	-0.05292	-0.03842	-0.02067	0.009833	-0.04267	1.017005	0.440693	0.149187
	70	0	0.464002	0.829181	0.329232	0.13580423	0.118822	0.255446	0.539285	0.76997	0.174488			
Conserved Element Deletions:	71	5075.77778	-206.528	-240.778	-26.7778	312.666667	362.7222	625.9722	581.9722	317.4722	249.6222	46.92154	0	0.889988
	71	0	0.001759	0.000117	0.673985	2.768E-08	2.66E-07	0	0	4.22E-06	6.92E-05			
TFBS Deletions:	72	77.2222222	-11.9722	-11.2222	5.277778	7.8888889	10.27778	26.52778	26.02778	11.02778	1.377778	14.29466	0	0.711366
	72	0	0.015771	0.01481	0.278726	0.0412784	0.03714	6.7E-07	1E-06	0.025685	0.759693			
TFBS Deletions/Total Deletions	73	0.0057778	0.000222	0.000222	0.000722	0.0004444	0.000472	0.000722	0.000972	0.000472	0.000222	2.528646	0.013339	0.303608
	73	0	0.436211	0.401729	0.013212	0.04988612	0.10068	0.013212	0.001047	0.10068	0.401729			
miRNA-BS disrupting Deletions:	74	3.1111111	-0.36111	0.088889	-0.86111	-1	-0.11111	-1.11111	-0.61111	-1.11111	-0.71111	1.447215	0.183257	0.199693
	74	0	0.551228	0.874273	0.157862	0.03821084	0.854341	0.069765	0.314418	0.069765	0.208224			
miRNA-BS disrupting Deletions/Total Deletions	75	0.0101111	-0.00061	0.001089	-0.00236	-0.0035556	-0.00086	-0.00411	-0.00261	-0.00411	-0.00271	2.221849	0.028771	0.276975
	75	0	0.746605	0.535501	0.214353	0.01887504	0.649016	0.032567	0.17025	0.032567	0.125671			
ESE-BS deletion Deletions:	76	7.5555556	-1.55556	-1.55556	-0.30556	-0.7777778	2.444444	2.444444	0.444444	1.194444	-2.55556	3.500541	0.001168	0.37638
	76	0	0.224537	0.19109	0.810448	0.43735139	0.058301	0.058301	0.72725	0.349934	0.033577			
ESE-BS deletion Deletions/Total Deletions	77	0.0453333	-0.00458	-0.00513	0.000167	-0.006	0.011667	0.004917	-0.00458	0.003167	-0.01353	2.116755	0.03735	0.267377
	77	0	0.507831	0.4246	0.980757	0.27051827	0.094769	0.477578	0.507831	0.647006	0.03789			
ESE-BS induction Deletions:	78	11.4444444	-1.69444	-0.44444	-1.69444	0.6666667	1.555556	2.305556	0.555556	-1.19444	-0.84444	1.252403	0.278664	0.177585
	78	0	0.311653	0.774171	0.311653	0.61082113	0.352689	0.17	0.739236	0.474866	0.585948			
ESE-BS induction Deletions/Total Deletions	79	0.0683333	-0.00133	0.004267	-0.00583	0.0026667	0.005917	0.001417	-0.00683	-0.01158	0.000467	0.626679	0.784957	0.097512
	79	0	0.889972	0.633672	0.545543	0.72441804	0.539835	0.883144	0.479124	0.231902	0.958395			
ESS-BS deletion Deletions:	80	18.6666667	-3.16667	-2.66667	-2.66667	-0.7777778	0.833333	1.833333	3.333333	-0.41667	0.933333	1.825723	0.076043	0.239416
	80	0	0.146281	0.186873	0.22012	0.64685689	0.700127	0.397818	0.126522	0.847229	0.642203			
ESS-BS deletion Deletions/Total Deletions	81	0.1292222	-0.00222	-0.00502	-0.01297	-0.0103333	-0.00397	-0.01047	0.000278	-0.01172	0.019778	1.110655	0.370019	0.160716
	81	0	0.871009	0.692717	0.344763	0.33741703	0.771674	0.445103	0.983805	0.39295	0.122779			
ESS-BS induction Deletions:	82	5.1111111	-0.36111	-0.11111	-0.36111	-0.8888889	0.888889	1.888889	0.138889	-2.36111	-1.71111	2.551223	0.012602	0.305491
	82	1E-12	0.732403	0.909719	0.732403	0.28515909	0.401011	0.076997	0.895331	0.02807	0.084199			
ESS-BS induction Deletions/Total Deletions	83	0.0352222	0.003778	0.003378	-0.00047	-0.0072222	0.003278	0.005278	-0.00397	-0.01747	-0.01082	2.291919	0.024156	0.283236
	83	0	0.58928	0.602992	0.946138	0.19064096	0.63939	0.451173	0.570305	0.014532	0.098732			

Splicing Change Deletions:	84	162.444444	0.055556	-5.84444	-8.19444	3.2222222	8.305556	9.555556	9.305556	1.055556	9.755556	5.661094	7.4E-06	0.49394
	84	0	0.988525	0.106489	0.036903	0.28964092	0.034498	0.015537	0.018328	0.784706	0.008059			
Splicing Change Deletions/Total Deletions	85	0.4624444	0.015306	0.006156	-0.00469	-0.0005556	0.004556	-0.02069	-0.01319	-0.00769	0.019956	3.473104	0.00125	0.374535
	85	0	0.101854	0.474827	0.612603	0.93905243	0.623138	0.02823	0.157392	0.407324	0.022832			
Protein motif disrupting Deletions:	86	9.5555556	0.944444	-2.75556	-2.55556	-0.4444444	0.444444	0.194444	-0.55556	-0.55556	-1.35556	1.257309	0.275844	0.178157
	86	0	0.546536	0.061072	0.105719	0.71733565	0.776374	0.901077	0.722591	0.722591	0.352052			
Protein motif disrupting Deletions/Total Deletions	87	0.6681111	0.079639	-0.03691	-0.05561	-0.0045556	-0.00536	-0.07586	-0.10161	-0.12511	-0.02631	1.826608	0.075881	0.239505
	87	0	0.189781	0.510587	0.358283	0.92333708	0.92921	0.211384	0.095648	0.041257	0.638813			
Conserved Element Rearrangements:	88	1129.44444	-56.1944	-56.8444	-12.6944	50.6666667	83.30556	160.3056	160.3056	65.55556	37.75556	23.77868	0	0.803913
	88	0	0.013929	0.007608	0.570253	0.00500552	0.000379	6.52E-10	6.52E-10	0.004423	0.072001			
TFBS Rearrangements:	89	15.2222222	-0.97222	-2.22222	0.027778	-0.8888889	0.527778	7.027778	6.027778	2.027778	1.177778	5.357659	1.45E-05	0.480178
	89	0	0.598741	0.197369	0.987992	0.53983258	0.77498	0.000293	0.001659	0.27407	0.492534			
TFBS Rearrangements/Total Rearrangements	90	0.0024444	0.000306	0.000156	0.000306	-0.0002222	5.56E-05	0.000806	0.000556	5.56E-05	0.000356	1.769596	0.08698	0.233777
	90	0	0.382095	0.630976	0.382095	0.41756916	0.873386	0.023421	0.114372	0.873386	0.273968			
miRNA-BS disrupting Rearrangements:	91	0	0	0	0.25	0	0	0.25	0.5	0	0	2.766154	0.007335	0.322917
	91	1	1	1	0.092791	1	1	0.092791	0.001103	1	1			
miRNA-BS disrupting Rearrangements/Total Rearrangements	92	0	0	0	0.01125	0	0	0.0085	0.017	0	0	2.502146	0.014258	0.301386
	92	1	1	1	0.045964	1	1	0.129142	0.003064	1	1			
ESE-BS deletion Rearrangements:	93	15.3333333	-0.33333	0.266667	-1.33333	1.4444444	0.916667	3.416667	4.166667	2.416667	1.066667	2.599561	0.011159	0.309488
	93	0	0.821955	0.846205	0.369412	0.21639537	0.536531	0.023648	0.006242	0.106133	0.438805			
ESE-BS deletion Rearrangements/Total Rearrangements	94	0.0626667	-0.00067	0.000933	-0.00492	0.0018889	-0.00017	0.008833	0.010833	0.005083	0.001133	1.313051	0.245371	0.184598
	94	0	0.905326	0.857639	0.381749	0.66770617	0.976278	0.118388	0.056586	0.365915	0.82758			
ESE-BS induction Rearrangements:	95	15.7777778	-0.27778	0.422222	-0.02778	0.5555556	1.472222	1.472222	1.972222	1.222222	0.222222	0.679807	0.738557	0.104912
	95	0	0.834647	0.732542	0.983343	0.59491946	0.270681	0.270681	0.141475	0.359804	0.857213			
ESE-BS induction Rearrangements/Total Rearrangements	96	0.0647778	-0.00078	0.001222	-2.8E-05	-0.0016667	0.001722	0.000972	0.001722	-2.8E-05	-0.00278	0.18006	0.997095	0.03011
	96	0	0.882725	0.802814	0.995796	0.6871356	0.744017	0.85371	0.744017	0.995796	0.570739			
ESS-BS deletion Rearrangements:	97	9.6666667	-0.91667	0.533333	0.083333	3.1111111	3.083333	3.583333	2.833333	2.333333	3.133333	5.632314	7.9E-06	0.492666
	97	0	0.391095	0.590238	0.937684	0.00039036	0.004992	0.001234	0.009557	0.031462	0.002238			
ESS-BS deletion Rearrangements/Total Rearrangements	98	0.0757778	-0.00403	0.007022	-2.8E-05	0.0233333	0.020722	0.018472	0.010472	0.012222	0.022822	5.354606	1.46E-05	0.480035
	98	0	0.565702	0.282129	0.996835	6.4633E-05	0.004132	0.010102	0.138089	0.084407	0.000772			
ESS-BS induction Rearrangements:	99	14.8888889	-1.63889	-1.88889	-1.88889	-1.4444444	-1.38889	-0.38889	0.611111	-0.38889	-3.68889	2.026442	0.046669	0.258923
	99	0	0.209607	0.120425	0.148947	0.15929334	0.286865	0.76465	0.638196	0.76465	0.003072			
ESS-BS induction Rearrangements/Total Rearrangements	100	0.1177778	-0.00928	-0.01218	-0.01703	-0.0138889	-0.01603	-0.01453	-0.01028	-0.01128	-0.03218	2.265703	0.025791	0.280906
	100	0	0.299264	0.143735	0.059119	0.04996375	0.075217	0.106091	0.250619	0.207899	0.000219			
Splicing Change Rearrangements:	101	14.1111111	-0.36111	-0.51111	1.138889	-1.1111111	-0.36111	1.888889	2.138889	-0.61111	0.888889	2.337874	0.021533	0.287283
	101	0	0.741369	0.614963	0.299679	0.19809062	0.741369	0.087602	0.053803	0.576772	0.382602			
Splicing Change Rearrangements/Total Rearrangements	102	0.2515556	0.009944	0.011244	0.020194	-0.0302222	-0.01731	-0.00381	0.000444	-0.01931	0.009444	1.855893	0.070713	0.242414
	102	0	0.599798	0.522912	0.288193	0.04504762	0.362202	0.840724	0.981272	0.309767	0.591381			
Protein motif disrupting Rearrangements:	103	18	-2.5	-3.2	0	0	-3.25	1.25	1.25	1.25	-2.4	2.176187	0.032232	0.272836
	103	0	0.168999	0.059446	1	1	0.075163	0.489324	0.489324	0.489324	0.155065			
Protein motif disrupting Rearrangements/Total Rearrangements	104	0.417	-0.0615	-0.0492	-0.02025	-0.0043333	-0.06325	0.02775	-0.01625	-0.00825	-0.051	2.042055	0.044911	0.260398
	104	0	0.050045	0.09005	0.513375	0.85828617	0.044042	0.371104	0.599732	0.789751	0.079129			
Nonsense SNPs:	105	30.8888889	-0.38889	-2.48889	-0.38889	3.5555556	4.111111	11.36111	7.611111	3.111111	4.511111	7.720475	1E-07	0.571021
	105	0	0.855895	0.213116	0.855895	0.03730183	0.058195	1.26E-06	0.00067	0.149395	0.025902			
Nonsense SNPs/Total SNPs	106	0.0018889	-0.00014	-0.00049	-0.00039	-0.0001111	0.000111	0.000111	0.000111	-0.00014	0.000111	1.824148	0.076331	0.239259
	106	0	0.547706	0.02508	0.095301	0.53982687	0.630377	0.630377	0.630377	0.547706	0.604213			
Frameshift Structural Variants:	107	190.333333	1.166667	-6.33333	4.916667	1.6666667	4.416667	12.16667	13.41667	14.66667	-0.33333	6.307782	1.8E-06	0.520969
	107	0	0.765704	0.084704	0.211695	0.58763927	0.261364	0.002664	0.001003	0.000357	0.926891			
Frameshift Structural Variants/Total Variants	108	0.4153333	0.003167	-0.00433	0.005667	-0.0058889	-0.00158	0.002167	-0.00783	0.004167	-0.00833	1.589259	0.132817	0.215077
	108	0	0.591324	0.429195	0.337798	0.2053241	0.788178	0.713189	0.186534	0.480242	0.130824			
Frameshift Insertions:	109	104.666667	1.083333	-5.46667	1.333333	0.4444444	-0.66667	5.583333	2.583333	6.583333	-2.06667	3.389196	0.00154	0.368824
	109	0	0.688129	0.031882	0.621397	0.83365793	0.804827	0.041571	0.339842	0.016911	0.410293			
Frameshift Insertions/Total Insertions	110	0.5975954	0.007779	-0.00645	0.000138	-0.0049943	-0.02066	0.010856	-0.01574	0.013665	-0.00636	2.27653	0.025103	0.28187
	110	0	0.435746	0.486328	0.988966	0.52324996	0.04116	0.277774	0.117286	0.172988	0.492015			
Frameshift Deletions:	111	80.1111111	2.138889	-0.51111	3.138889	0.7777778	5.138889	5.638889	10.88889	7.888889	2.488889	3.6337	0.000841	0.385183
	111	0	0.451862	0.846124	0.27074	0.72685263	0.073515	0.05011	0.000264	0.006837	0.346189			
Frameshift Deletions/Total Deletions	112	0.8568434	0.034779	0.027034	0.02432	0.005874	0.005333	-0.01838	0.004925	-0.00255	0.027765	1.891227	0.064922	0.245894
	112	0	0.0489	0.097637	0.165326	0.66721179	0.759327	0.292869	0.777255	0.883597	0.089078			
Frameshift Rearrangements:	113	5.5555556	-2.05556	-0.35556	0.444444	0.4444444	-0.05556	0.944444	-0.05556	0.194444	-0.75556	1.691132	0.104749	0.225751
	113	0	0.022864	0.66562	0.616163	0.52304864	0.949989	0.28835	0.949989	0.826273	0.359612			
Frameshift Rearrangements/Total Rearrangements	114	0.0291358	-0.0108	-0.0017	0.00225	0.0012318	-0.00049	0.003056	-0.00292	-0.00084	-0.0052	1.642595	0.117364	0.220702
	114	0	0.017752	0.681215	0.614355	0.72498024	0.911889	0.494162	0.51412	0.849862	0.211841			
Splicing Change Variants:	115	2016	41	21.8	8.75	198.555556	212.5	185	192.5	145	227.6	60.04967	0	0.911921
	115	0	0.035648	0.223635	0.648717	0	0	0	0	1.36E-10	0			
Splicing Change Variants/Total Variants	116	0.447	0.0095	0.0082	0.0005	-0.0064444	-0.0045	-0.01675	-0.01425	-0.009	-0.0038	21.23933	0	0.785498
	116	0	0.000532	0.001195	0.84866	0.00245818	0.089299	1.65E-08	7.5E-07	0.00098	0.121463			
Probably Damaging nscSNPs:	117	985	0.75	-20.8	25.75	202.777778	191	243.75	191.75	160	207.6	200.1863	0	0.971843
	117	0	0.948082	0.055011	0.028139	0	0	0	0	0	0			
Probably Damaging nscSNPs/Total nscSNPs	118	0.1114444	-0.00069	-0.00264	0.001556	0.0074444	0.007306	0.010806	0.005806	0.006306	0.007156	48.62949	0	0.89344
	118	0	0.471288	0.004103	0.109317	0	1.16E-10	0	7.07E-08	8.54E-09	2E-11			
Possibly Damaging nscSNPs:	119	880.111111	7.138889	8.488889	1.138889	143.222222	131.1389	170.1389	167.8889	97.88889	159.4889	95.52843	0	0.94276
	119	0	0.550084	0.444287	0.923944	0	0	0	0	9E-12	0			
Possibly Damaging nscSNPs/Total nscSNPs	120	0.0995556	0.000444	0.000444	-0.00106	0.0027778	0.002694	0.004694	0.004694	0.000944	0.003844	8.088954	1E-07	0.582402
	120	0	0.675162	0.651666	0.321061	0.00131594	0.013004	3.37E-05	3.37E-05	0.374282	0.000209			
Protein motif damaging Variants:	121	305.666667	-4.66667	-8.46667	1.333333	44	34.58333	69.58333	58.08333	29.33333	53.33333	27.65274	0	0.826621
	121	0	0.569273	0.26758	0.870685	2.541E-09	7.04E-05	3E-12	9.28E-10	0.000614	1.27E-09			
Protein motif damaging Variants/Total Variants	122	0.1338889	-0.00239	-0.00229	-0.00189	0.001	-0.00039	0.009361	0.006361	-0.00164	0.004511	3.85018	0.000495	0.398975
	122	0	0.407285	0.392399	0.511909	0.6577546	0.892421	0.001711	0.029766	0.56916	0.094414			
TFBS Disrupting Variants:	123	126.444444	-19.6944	-18.6444	6.055556	1.5555556	7.055556	36.80556	36.55556	15.05556	-3.24444	16.22518	0	0.736665
	123	0	0.004179	0.003523	0.365065	0.76614906	0.291803	5.42E-07	6.29E-07	0.026602	0.600346			
TFBS Disrupting Variants/Total Variants	124	0.0006667	-0.00067	-0.00047	0.000333	-0.0004444	-0.00042	0.000333	0.000333	8.33E-05	-0.00047	5.528882	9.9E-06	0.488034
	124	3.9966E-06	0.006917	0.039338	0.16809	0.02075687	0.086131	0.16809	0.16809	0.728661	0.039338			
miRNA-BS Disrupting Variants:	125	45.1111111	-4.36111	-2.51111	-0.61111	4.5555556	4.138889	15.13889	14.38889	6.138889	3.088889	12.0524	0	0.675114
	125	0	0.100988	0.305962	0.816449	0.03020687	0.119221	2.19E-07	6.75E-07	0.022223	0.208839			
miRNA-BS Disrupting Variants/Total Variants	126	0.0137778	-0.00103	-0.00038	-0.00028	-0.0004444	-0.00053	0.002222	0.001972	0.000222	-0.00078	3.866431	0.000476	0.399985
	126	0	0.189752	0.601585	0.721437	0.46777236	0.49867	0.005575	0.013337	0.775429	0.283979			
ESE-BS Disrupting Variants:	127	1438.66667	1.083333	-3.46667	2.083333	172.333333	170.5833	179.5833	186.5833	138.3333	185.3333	120.3749	0	0.954032
	127	0	0.931952	0.76853	0.869575	0	0	0	0	0	0			
ESE-BS Disrupting Variants/Total Variants	128	0.1071111	-0.00061	-0.00031	-0.00111	-0.0001111	0.000389	-0.00086	-0.00061	-0.00061	8.89E-05	1.000424	0.454004	0.147112
	128	0	0.360503	0.615223	0.098788	0.83166526	0.559906	0.198954	0.360503	0.360503	0.88571			
ESS-BS Disrupting Variants:	129	588.555556	7.444444	-1.75556	19.94444	83.1111111	80.44444	103.9444	103.1944	66.69444	86.24444	104.7804	0	0.94755
	129	0	0.261443	0.774434	0.003431	0	0	0	0	0	0			
ESS-BS Disrupting Variants/Total Variants	130	0.1164444	0.000806	-0.00024	0.001806	-0.0002222	0.000556	0.001306	0.001806	-0.00019	0.000756	1.263457	0.272342	0.178872
	130	0	0.450711	0.804872	0.093681	0.79044958	0.602547	0.22316	0.093681	0.855251	0.445994			
**Total Likely Functional Variants:**	131	***6103.1111***	30.38889	-19.7111	62.13889	***772***	***762.389***	***907.389***	***877.889***	***606.139***	***835.089***	334.3315	0	***0.98295***
	131	0	0.380851	0.539684	0.075757	0	0	0	0	0	0			
**Total genotypes (not just homozygous)**	132	6042271	-83419.3	-57488	-51149.3	-395516.33	-375283	-428364	-442378	-332702	-444922	222.3361	0	0.974577
	132	0	8.19E-05	0.002709	0.012324	0	0	0	0	0	0			
synonymous to nonsynonymous rate	133	1.1175796	0.003131	-0.00493	0.001511	-0.01258	-0.00754	-0.00962	-0.00321	-0.00066	-0.01253	4.460261	0.000115	0.434712
	133	0	0.469667	0.221603	0.726823	0.00040411	0.084479	0.028794	0.458578	0.879001	0.002551			
Likely functional variant rate	134	0.0010102	1.91E-05	6.4E-06	1.89E-05	0.0002074	0.000201	0.000239	0.000237	0.000165	0.000229	393.7368	0	0.985483
	134	0	0.02457	0.408113	0.02654	0	0	0	0	0	0			
Likely functional variant rate (minus probdam)	135	0.0008645	1.59E-05	3.6E-06	1.75E-05	0.0001718	0.000169	0.000197	0.000195	0.000139	0.000189	383.6934	0	0.985109
	135	0	0.025894	0.58146	0.014894	0	0	0	0	0	0			
Functional Nonsense SNPs/Total Variants	136	0.0000051	0	-4E-07	0	0.000001	1.1E-06	2.4E-06	1.8E-06	8E-07	1.2E-06	12.13608	0	0.676629
	136	0	0.981384	0.285741	0.953314	0.00108809	0.005224	9.49E-09	9.69E-06	0.025172	0.000723			
Functional Frameshift Structural Variants/Total Variants	137	0.0000315	6E-07	-8E-07	1.1E-06	0.0000025	2.9E-06	4.6E-06	4.9E-06	4.4E-06	2.4E-06	19.72561	0	0.772777
	137	0	0.341666	0.222483	0.105226	9.1351E-06	5.25E-05	2.33E-09	3.3E-10	6.58E-09	0.000177			
Functional Insertions/Total Variants	138	0.0000173	4E-07	-7E-07	4E-07	0.0000013	0.000001	2.3E-06	1.8E-06	2.2E-06	0.000001	10.51088	0	0.644409
	138	0	0.346354	0.076245	0.409257	0.00046918	0.024578	2.25E-06	0.000119	8.22E-06	0.017864			
Functional Deletions/Total Variants	139	0.0000133	5E-07	0	6E-07	0.0000011	1.8E-06	0.000002	0.000003	2.2E-06	1.5E-06	8.639792	0	0.598332
	139	0	0.28222	0.926961	0.209047	0.00856776	0.000671	0.000146	9.57E-08	5.63E-05	0.001962			
Functional Rearrangements/Total Variants	140	0.0000009	-3E-07	-1E-07	1E-07	0.0000001	1E-07	2E-07	1E-07	1E-07	-1E-07	2.026827	0.046624	0.258959
	140	0	0.031421	0.711349	0.587205	0.23106371	0.739313	0.118933	0.67884	0.562835	0.662684			
Functional Splicing Change Variants/Total Variants	141	0.0003337	1.15E-05	6.8E-06	4.3E-06	0.0000585	5.96E-05	5.84E-05	6.07E-05	4.48E-05	6.71E-05	129.9702	0	0.957281
	141	0	0.003929	0.06021	0.27249	0	0	0	0	0	0			
Probably Damaging nscSNPs/Total Variants	142	0.000163	2.4E-06	-1.9E-06	5.7E-06	0.0000473	4.45E-05	5.58E-05	4.71E-05	3.75E-05	0.00005	258.0904	0	0.978021
	142	0	0.308554	0.382897	0.01734	0	0	0	0	0	0			
Possibly Damaging nscSNPs/Total Variants	143	0.0001457	3.2E-06	2.8E-06	1.4E-06	0.0000355	3.28E-05	4.14E-05	4.15E-05	2.56E-05	0.00004	148.3005	0	0.962362
	143	0	0.171071	0.193309	0.546475	0	0	0	0	0	0			
Functional Protein motif damaging Variants/Total Variants	144	0.0000506	-1E-07	-9E-07	6E-07	0.0000113	9.4E-06	1.63E-05	1.44E-05	8.1E-06	1.35E-05	50.15779	0	0.89635
	144	0	0.954652	0.487302	0.654771	0	9.32E-09	0	0	4.2E-07	0			
Functional TFBS Disrupting Variants/Total Variants	145	0.0000209	-3E-06	-2.9E-06	1.2E-06	0.0000017	2.6E-06	8.2E-06	8.2E-06	3.9E-06	1.1E-06	22.31405	0	0.793697
	145	0	0.010402	0.0076	0.297935	0.05574434	0.023506	7.87E-10	7.09E-10	0.00117	0.307007			
Functional miRNA-BS Disrupting Variants/Total Variants	146	0.0000075	-6E-07	-3E-07	0	0.0000013	1.2E-06	3.3E-06	3.2E-06	1.5E-06	1.1E-06	18.59608	0	0.762257
	146	0	0.165451	0.40291	0.932798	0.00032479	0.00773	4.2E-10	1.12E-09	0.001222	0.007392			
Functional ESE-BS Disrupting Variants/Total Variants	147	0.0002381	3.5E-06	1.7E-06	2.4E-06	0.0000472	4.59E-05	5.01E-05	5.21E-05	3.81E-05	0.000052	212.2103	0	0.973396
	147	0	0.174787	0.470599	0.359411	0	0	0	0	0	0			
Functional ESS-BS Disrupting Variants/Total Variants	148	0.0000974	2.6E-06	6E-07	4.2E-06	0.0000215	2.06E-05	2.59E-05	2.61E-05	1.73E-05	2.31E-05	189.5775	0	0.970314
	148	0	0.039509	0.582063	0.001348	0	0	0	0	0	0			
