# Supplementary material for: Clinical Implications of Human Population Differences in Genome-Wide Rates of Functional Genotypes
Source: Front Genet. 2012 Nov 1;3:211. doi: 10.3389/fgene.2012.00211 (PMC3485509; doi:10.3389/fgene.2012.00211)
Supplement: Supplementary Data Sheet S5 — Regression analysis results for ancestral allele-based variants: novel variants. [file 32001_Schork_DataSheet5.PDF]

Ancestral Allele Based Variants: All Novel Variants
Row 1: Regression Coefficients
Row 2: P-values for Regression Coefficients

	Var Cat	Y-int	LWK	ASW	MKK	CEU	TSI	CHB	JPT	GIH	MEX	Overall F	p-val	R-Sqr
Coding SNPs:	1	1505.222	186.277778	-81.8222	-200.222	-797.33333	-705.472	-576.9722	-592.4722	-507.972	-733.82222	39.46296	0	0.87186
	1	0	0.03835723	0.32101	0.026376	0	2.4E-11	9.84E-09	4.795E-09	2.3E-07	0			
Nonsynonymous SNPs:	2	861.4444	106.055556	-43.8444	-115.944	-422.44444	-351.694	-283.4444	-298.4444	-246.694	-380.04444	29.1159	0	0.833887
	2	0	0.05066041	0.37858	0.033111	0	7.84E-09	1.3E-06	4.331E-07	1.74E-05	9E-11			
Synonymous SNPs:	3	629.5556	75.6944444	-37.7556	-81.5556	-369.88889	-349.556	-291.5556	-291.0556	-257.056	-351.75556	49.17798	0	0.894503
	3	0	0.04807127	0.283105	0.033595	0	0	6.6E-11	7E-11	2.95E-09	0			
Nonsense SNPs:	4	14.22222	4.5277778	-0.22222	-2.72222	-5	-4.22222	-1.972222	-2.972222	-4.22222	-2.0222222	2.765212	0.007352	0.322842
	4	0	0.07543307	0.924212	0.28149	0.013337	0.096838	0.43429	0.2400316	0.096838	0.3879827			
Untranslated Region SNPs:	5	1961.556	190.194444	-108.556	-208.806	-1166.8889	-1089.56	-934.5556	-938.5556	-799.056	-1081.1556	48.48056	0	0.893148
	5	0	0.1128856	0.326798	0.082357	0	0	3.7E-11	3.3E-11	4.25E-09	0			
Non-coding RNA SNPs:	6	50304.11	5899.38889	-3744.51	-6303.36	-30809.444	-28426.4	-24717.61	-25660.36	-22530.6	-29405.511	65.45179	0	0.918599
	6	0	0.03338537	0.142126	0.023343	0	0	0	0	7E-12	0			
Intronic SNPs:	7	74366.78	9173.22222	-5245.98	-8668.03	-45147	-42273.3	-36462.53	-37145.53	-32940.3	-42798.178	60.74704	0	0.912844
	7	0	0.03073693	0.178438	0.040831	0	0	1E-12	0	3.3E-11	0			
Intergenic SNPs:	8	98132.44	11656.0556	-6400.84	-11211.4	-60268.667	-55864.4	-49392.94	-50657.19	-44508.9	-57447.844	60.78594	0	0.912895
	8	0	0.04007745	0.21979	0.048049	0	0	1E-12	0	2.5E-11	0			
**Total SNPs:**	9	176332.8	21233.2222	-11878.4	-20355	-107593.89	-100136	-87538.28	-89529.28	-78914	-102281.18	60.85064	0	0.912979
	9	0	0.0358206	0.201115	0.043927	0	0	1E-12	0	2.8E-11	0			
Coding Insertions:	10	34.44444	-3.9444444	-3.64444	0.055556	-14.222222	-3.19444	-3.194444	-8.944444	-3.94444	-4.8444444	4.529717	9.72E-05	0.438513
	10	0	0.28151724	0.283706	0.987845	4.546E-06	0.382415	0.382415	0.0164015	0.281517	0.1555023			
In-frame Insertions:	11	4.111111	-2.3611111	-0.91111	0.138889	-2.2222222	-2.36111	-1.611111	-1.361111	-1.61111	-2.9111111	1.907099	0.062469	0.247447
	11	2.96E-08	0.04974668	0.409061	0.906779	0.0193026	0.049747	0.177278	0.2534346	0.177278	0.00991593			
Out-of-frame Insertions:	12	8.888889	-4.8888889	-3.68889	-1.88889	-4.7777778	-3.63889	-2.888889	-5.138889	-2.13889	-5.4888889	4.325332	0.000158	0.427179
	12	0	0.00117875	0.00755	0.194866	7.46E-05	0.014032	0.049265	0.0006824	0.142837	0.00011426			
Frameshift Insertions:	13	21.44444	3.3055556	0.955556	1.805556	-7.2222222	2.805556	1.305556	-2.444444	-0.19444	3.5555556	2.917767	0.005008	0.334692
	13	0	0.32537744	0.758617	0.590192	0.0074604	0.403406	0.696817	0.4663288	0.9537	0.2550042			
Untranslated region Insertions:	14	224.8889	-7.3888889	-9.48889	-12.3889	-104.88889	-83.6389	-74.13889	-82.88889	-63.1389	-74.688889	24.67602	0	0.809687
	14	0	0.57612957	0.439729	0.349598	0	2.08E-08	3.75E-07	2.626E-08	9.24E-06	5.5221E-08			
Non-coding RNA Insertions:	15	5711.889	-419.63889	-487.689	-662.639	-2460.8889	-1946.89	-2055.889	-2192.389	-1659.14	-2119.2889	51.00964	0	0.897905
	15	0	0.05235936	0.015934	0.002674	0	0	0	0	5.3E-11	0			
Intronic Insertions:	16	9014.222	-764.22222	-778.622	-1040.72	-3877.6667	-3072.97	-3186.222	-3438.472	-2736.47	-3444.2222	45.84126	0	0.887687
	16	0	0.03395331	0.020345	0.004395	0	1E-12	0	0	6.7E-11	0			
Intergenic Insertions:	17	11196.33	-739.83333	-881.533	-1274.08	-4961	-3924.83	-4088.833	-4363.833	-3422.08	-4253.9333	62.37356	0	0.914923
	17	0	0.06236132	0.017646	0.001731	0	0	0	0	1E-12	0			
**Total Insertions:**	18	20497.44	-1511.1944	-1676.84	-2327.44	-8970.4444	-7095.44	-7363.944	-7907.694	-6233.69	-7787.4444	54.41076	0	0.903672
	18	0	0.0486687	0.019153	0.002891	0	0	0	0	7E-12	0			
Coding Deletions:	19	37	0.25	2.6	0	-14.777778	-8.75	-8.5	-8	-0.75	-8.4	6.340316	1.7E-06	0.522253
	19	0	0.94801452	0.465942	1	5.667E-06	0.025141	0.029443	0.0400242	0.844942	0.02072232			
In-frame Deletions:	20	3.111111	2.6388889	2.088889	-0.86111	-0.5555556	-0.11111	-0.611111	-0.361111	0.388889	-0.7111111	2.125622	0.036539	0.268196
	20	1.19E-05	0.02933692	0.061881	0.470008	0.5521456	0.925585	0.6078	0.76154	0.743831	0.52020104			
Inter-Codon Deletions:	21	8.111111	-1.8611111	-0.51111	2.638889	-4.3333333	-4.11111	-0.861111	-0.361111	-0.86111	-2.7111111	3.988539	0.000354	0.40747
	21	0	0.254213	0.734719	0.107654	0.0010926	0.013396	0.596409	0.8241028	0.596409	0.07558394			
Frameshift Deletions:	22	25.77778	-0.5277778	1.022222	-1.77778	-9.8888889	-4.52778	-7.027778	-7.277778	-0.27778	-4.9777778	4.631727	7.65E-05	0.444004
	22	0	0.85672703	0.706486	0.543603	5.12E-05	0.124695	0.018548	0.0149049	0.924291	0.06995804			
Untranslated region Deletions:	23	279.7778	30.2222222	-7.17778	-30.7778	-148.88889	-134.278	-141.0278	-123.7778	-120.278	-129.37778	61.88383	0	0.914308
	23	0	0.0326243	0.578497	0.02966	0	0	0	0	1E-12	0			
Non-coding RNA Deletions:	24	7347.111	258.888889	-524.111	-767.861	-3777.2222	-3369.86	-3461.361	-3615.611	-3060.11	-3254.5111	119.2836	0	0.953631
	24	0	0.28872021	0.022688	0.002286	0	0	0	0	0	0			
Intronic Deletions:	25	11336.33	277.916667	-822.533	-1432.58	-5890.3333	-5310.08	-5410.333	-5507.083	-4664.33	-5128.3333	111.8636	0	0.950707
	25	0	0.47246364	0.024333	0.000403	0	0	0	0	0	0			
Intergenic Deletions:	26	14479.56	354.694444	-992.756	-1562.06	-7426.5556	-6708.31	-6844.056	-7114.306	-5996.56	-6449.1556	106.3183	0	0.948269
	26	0	0.48333233	0.037235	0.002794	0	0	0	0	0	0			
**Total Deletions:**	27	26165.44	668.555556	-1816.84	-3029.19	-13499.222	-12181.4	-12416.19	-12771.94	-10797.9	-11724.844	109.6261	0	0.949751
	27	0	0.45842201	0.03251	0.001216	0	0	0	0	0	0			
Coding rearrangements:	28	27.22222	-2.4722222	4.377778	-3.72222	-11.222222	-0.97222	-4.722222	-5.972222	-5.72222	-10.422222	5.679571	7.1E-06	0.494755
	28	0	0.45642209	0.157619	0.263406	5.016E-05	0.76922	0.157119	0.0748417	0.087542	0.00113125			
In-frame rearrangements:	29	22.77778	-1.7777778	2.022222	-4.52778	-9.8888889	-4.52778	-4.777778	-4.027778	-5.02778	-8.7777778	4.586507	0.000085	0.441583
	29	0	0.54277546	0.456061	0.123934	4.961E-05	0.123934	0.104846	0.1703459	0.088217	0.00178397			
Frameshift rearrangements:	30	4.444444	-0.6944444	2.355556	0.805556	-1.3333333	3.555556	0.055556	-1.944444	-0.69444	-1.6444444	5.165249	2.24E-05	0.471056
	30	7.3E-10	0.53602888	0.026227	0.473074	0.1326017	0.002199	0.960459	0.0861499	0.536029	0.11723499			
Untranslated region rearrangements:	31	64.33333	-6.8333333	-5.93333	-11.0833	-31.222222	-28.0833	-22.58333	-26.08333	-20.0833	-35.533333	26.17425	0	0.818604
	31	0	0.08866648	0.110768	0.006632	0	1E-09	2.82E-07	8.003E-09	3.27E-06	0			
Non-coding RNA rearrangements:	32	2240.889	-220.63889	-256.489	-306.139	-1085.6667	-924.389	-819.6389	-983.1389	-787.889	-1139.6889	49.95233	0	0.895969
	32	0	0.02760718	0.006304	0.002631	0	0	5E-12	0	2E-11	0			
Intronic rearrangements:	33	3085.667	-248.66667	-452.667	-462.917	-1607.7778	-1350.17	-1323.667	-1407.167	-1203.17	-1578.2667	60.44484	0	0.912446
	33	0	0.05966984	0.000363	0.000674	0	0	0	0	0	0			
Intergenic rearrangements:	34	4422.222	-453.72222	-529.422	-617.472	-2227.1111	-1880.47	-1762.722	-1938.222	-1621.97	-2263.4222	56.44795	0	0.906824
	34	0	0.01787286	0.003254	0.001531	0	0	0	0	1E-12	0			
**Total rearrangements:**	35	7612.778	-709.02778	-984.778	-1098.78	-3883.4444	-3261.78	-3117.028	-3383.028	-2855.53	-3894.9778	59.08715	0	0.910614
	35	0	0.02899262	0.001375	0.000949	0	0	0	0	0	0			
**Total number of variants:**	36	230608.4	19681.5556	-16356.8	***-26810.4***	***-133947***	***-122674***	***-110435***	***-113591.9***	***-98801.2***	***-125688.44***	68.23478	0	***0.92166***
	36	0	0.08976849	0.127927	0.021993	0	0	0	0	2E-12	0			
Conserved Element SNPs:	37	10997	1372.5	-668.8	-1174.25	-6364.4444	-5818.5	-5018.75	-5135.25	-4478.25	-6024.6	52.90963	0	0.901209
	37	0	0.03184085	0.253869	0.065071	0	0	2.2E-11	1E-11	8.06E-10	0			
TFBS SNPs:	38	4.222222	0.2777778	-0.22222	-0.97222	-2.6666667	-0.72222	-1.722222	-1.472222	-1.22222	-2.6222222	2.701536	0.008631	0.31777
	38	3.9E-10	0.78995575	0.818406	0.352623	0.0016859	0.489253	0.101973	0.1609981	0.243473	0.00830962			
TFBS SNPs/Total SNPs	39	0	0	0	0	0	0	0	0	0	0	0	1	0
	39													
miRNA-BS disrupting SNPs:	40	20.33333	1.9166667	0.466667	-0.58333	-9.8888889	-9.08333	-10.58333	-6.583333	-5.08333	-10.533333	9.330221	0	0.616661
	40	0	0.45431805	0.844095	0.819523	5.287E-06	0.000673	9.4E-05	0.0119185	0.049994	3.252E-05			
miRNA-BS disrupting SNPs/Total SNPs	41	0.058667	-0.0044167	0.001933	0.001583	0.0032222	0.003833	-0.012167	0.0070833	0.008333	-0.0028667	0.876627	0.559722	0.131298
	41	0	0.61431513	0.812015	0.856523	0.639274	0.66177	0.167718	0.4196819	0.342878	0.72442544			
ESE-BS deletion SNPs:	42	55.88889	1.6111111	3.511111	-7.38889	-28.222222	-25.6389	-20.88889	-20.13889	-21.1389	-27.888889	16.50144	0	0.739927
	42	0	0.74881443	0.452949	0.145011	7.22E-10	2.81E-06	8.99E-05	0.0001505	7.55E-05	9.0044E-08			
ESE-BS deletion SNPs/Total SNPs	43	0.054778	-0.0055278	0.005822	-0.00253	0.0015556	0.000472	0.000472	0.0022222	-0.00403	-0.0023778	0.954222	0.49224	0.141278
	43	0	0.26015584	0.201947	0.605256	0.6850274	0.923005	0.923005	0.649481	0.410907	0.60042747			
ESE-BS induction SNPs:	44	68.77778	7.2222222	-9.97778	-4.02778	-33.666667	-27.2778	-23.27778	-27.77778	-26.0278	-34.377778	12.58429	0	0.684513
	44	0	0.28239206	0.111452	0.547646	1.504E-08	0.000117	0.000852	9.009E-05	0.000221	5.1242E-07			
ESE-BS induction SNPs/Total SNPs	45	0.067	-0.00175	-0.0072	0.0045	0.0044444	0.00875	0.005	-0.00225	-0.0045	-0.003	1.542206	0.147953	0.210047
	45	0	0.766746	0.191259	0.446439	0.3383867	0.141125	0.397814	0.7029775	0.446439	0.5840896			
ESS-BS deletion SNPs:	46	20	3.75	-0.4	-3	-12.111111	-10	-9.5	-10.75	-8.5	-12.4	13.76331	0	0.703527
	46	0	0.13129121	0.861167	0.225925	2.765E-08	0.000125	0.000249	4.278E-05	0.000936	8.0256E-07			
ESS-BS deletion SNPs/Total SNPs	47	0.053333	0.0016667	0.001467	-0.00283	-0.0075556	-0.00308	-0.006083	-0.011833	-0.00758	-0.0115333	1.112133	0.368967	0.160896
	47	0	0.81525392	0.824682	0.691325	0.1797838	0.665709	0.394939	0.1004859	0.28966	0.08489815			
ESS-BS induction SNPs:	48	26.11111	9.1388889	-2.91111	-3.36111	-13.333333	-11.3611	-9.361111	-12.61111	-12.6111	-13.511111	11.19376	0	0.658698
	48	0	0.00841216	0.354745	0.321549	3.622E-06	0.001229	0.007022	0.0003748	0.000375	5.1901E-05			
ESS-BS induction SNPs/Total SNPs	49	0.069222	0.0132778	-0.00462	0.004028	0.0037778	0.004278	0.006028	-0.008472	-0.01497	-0.0008222	0.735608	0.688108	0.112554
	49	0	0.30168543	0.697501	0.753181	0.7069889	0.738418	0.638091	0.5088784	0.244678	0.94484547			
Splicing Change SNPs:	50	3.666667	1.0833333	0.933333	-1.41667	-1.3333333	-2.41667	-1.666667	-1.916667	-0.66667	-0.6666667	2.081494	0.040751	0.264099
	50	3.82E-07	0.35928259	0.394617	0.231641	0.1522157	0.043369	0.160213	0.1071338	0.571904	0.54261564			
Splicing Change SNPs/Total SNPs	51	0.013111	0.0021389	0.004489	-0.00111	0.0056667	-0.00361	-0.000611	-0.002111	0.003139	0.0092889	0.927057	0.515444	0.13781
	51	0.00031	0.73169169	0.439011	0.858586	0.2490243	0.562987	0.921929	0.7350414	0.615016	0.11188768			
Protein Motif disrupting SNPs:	52	84.55556	17.6944444	-3.55556	-1.05556	-35.666667	-23.5556	-17.80556	-18.55556	-16.8056	-30.355556	14.01831	0	0.707341
	52	0	0.01169543	0.576542	0.877571	6.1E-09	0.000972	0.011203	0.0083437	0.016395	9.5658E-06			
Protein Motif disrupting SNPs/Total SNPs	53	0.278333	0.0159167	-0.00333	0.034167	0.0132222	0.030167	0.022417	0.0251667	0.017917	0.0170667	0.992843	0.460164	0.14616
	53	0	0.39266088	0.84667	0.069187	0.3655186	0.107662	0.229881	0.1782772	0.336296	0.32385316			
Conserved Element Insertions:	54	801.4444	11.3055556	-37.4444	-56.1944	-343.44444	-222.694	-261.4444	-268.4444	-211.944	-247.64444	43.69846	0	0.882825
	54	0	0.7283772	0.217687	0.08762	0	2.59E-09	1.8E-11	8E-12	1.01E-08	9E-12			
TFBS Insertions:	55	9.222222	-4.2222222	-2.02222	-2.72222	-4.7777778	-2.22222	-3.972222	-3.972222	-4.22222	-4.4222222	1.893024	0.064639	0.24607
	55	3E-12	0.03503562	0.270865	0.169969	0.0027989	0.26149	0.046937	0.0469371	0.035036	0.01787992			
TFBS Insertions/Total Insertions	56	0.003	-0.00125	-0.0004	-0.00075	-0.0001111	0.0005	-0.00025	0	-0.00025	-0.0002	0.613304	0.796264	0.09563
	56	8.95E-09	0.13377676	0.602519	0.365738	0.8639709	0.545831	0.762408	1	0.762408	0.79440869			
miRNA-BS disrupting Insertions:	57	0.555556	-0.0555556	-0.15556	-0.05556	-0.3333333	0.194444	-0.055556	-0.305556	-0.55556	-0.3555556	0.824603	0.606641	0.124476
	57	0.005667	0.87449028	0.633977	0.87449	0.2294757	0.580763	0.87449	0.3862708	0.117533	0.27817231			
miRNA-BS disrupting Insertions/Total Insertions	58	0.011333	-0.0020833	-0.00413	-0.00333	-0.0034444	0.007917	0.000167	-0.005583	-0.01133	-0.0065333	0.738363	0.685591	0.112928
	58	0.01409	0.79792516	0.584524	0.682176	0.5897777	0.33218	0.983654	0.4932626	0.166613	0.38822909			
ESE-BS deletion Insertions:	59	0.111111	0.6388889	-0.11111	-0.11111	0	0.138889	-0.111111	0.3888889	0.388889	0.0888889	2.476684	0.015201	0.299236
	59	0.384022	0.00676644	0.602241	0.628511	1	0.545524	0.628511	0.093541	0.093541	0.67660696			
ESE-BS deletion Insertions/Total Insertions	60	0.005556	0.0344444	-0.00556	-0.00556	0.0018889	0.003444	-0.005556	0.0369444	0.019944	0.0008444	2.747072	0.007696	0.321405
	60	0.46451	0.01376313	0.661615	0.684526	0.8601497	0.801042	0.684526	0.008452	0.147609	0.94692024			
ESE-BS induction Insertions:	61	0.222222	-0.2222222	-0.22222	-0.22222	-0.1111111	0.027778	-0.222222	0.0277778	0.027778	-0.0222222	0.594571	0.811791	0.092981
	61	0.069724	0.31025917	0.274612	0.310259	0.5168445	0.898688	0.310259	0.898688	0.898688	0.91261809			
ESE-BS induction Insertions/Total Insertions	62	0.010556	-0.0105556	-0.01056	-0.01056	-0.0046667	-0.00106	-0.010556	0.0041944	0.004194	-0.0041556	0.627904	0.783913	0.097684
	62	0.08074	0.32885255	0.29309	0.328853	0.5811978	0.92194	0.328853	0.697148	0.697148	0.67787755			
ESS-BS deletion Insertions:	63	0.111111	0.1388889	0.088889	-0.11111	-0.1111111	-0.11111	-0.111111	0.1388889	0.138889	-0.1111111	0.854551	0.579508	0.128416
	63	0.280287	0.45306277	0.604508	0.548032	0.444204	0.548032	0.548032	0.4530628	0.453063	0.51760325			
ESS-BS deletion Insertions/Total Insertions	64	0.012333	0.0054167	6.67E-05	-0.01233	-0.0123333	-0.01233	-0.012333	0.0104167	0.006917	-0.0123333	0.751093	0.673943	0.114652
	64	0.159885	0.73024729	0.99635	0.433266	0.3185279	0.433266	0.433266	0.5077954	0.659825	0.3987095			
ESS-BS induction Insertions:	65	0.222222	-0.2222222	-0.02222	0.027778	-0.2222222	-0.22222	-0.222222	-0.222222	-0.22222	0.1777778	1.349975	0.226737	0.188808
	65	0.041148	0.25219841	0.901344	0.885639	0.1456152	0.252198	0.252198	0.2521984	0.252198	0.32308768			
ESS-BS induction Insertions/Total Insertions	66	0.019222	-0.0192222	0.000778	-0.00147	-0.0192222	-0.01922	-0.019222	-0.019222	-0.01922	0.0115778	1.196135	0.312635	0.170971
	66	0.039909	0.24920747	0.959738	0.929327	0.1430972	0.249207	0.249207	0.2492075	0.249207	0.45332672			
Splicing Change Insertions:	67	23.44444	-4.4444444	-1.64444	-4.94444	-9.6666667	-5.44444	-7.194444	-8.694444	-8.19444	-10.244444	4.116608	0.00026	0.415123
	67	0	0.13095507	0.544238	0.093563	7.018E-05	0.065416	0.015853	0.0038853	0.006324	0.00031762			
Splicing Change Insertions/Total Insertions	68	0.440333	-0.0720833	-0.00833	-0.04658	-0.0081111	0.012667	-0.012833	-0.028083	-0.04383	-0.0737333	0.876916	0.559465	0.131335
	68	0	0.13469713	0.850988	0.33135	0.8287224	0.791005	0.788322	0.5572441	0.360495	0.09986246			
Protein motif disrupting Insertions:	69	9.555556	2.4444444	0.444444	2.194444	-2.1111111	0.194444	-1.805556	-2.305556	0.944444	0.6444444	1.707829	0.100707	0.227473
	69	0	0.20234619	0.801656	0.251855	0.1609847	0.918733	0.345021	0.2288745	0.620502	0.71574494			
Protein motif disrupting Insertions/Total Insertions	70	0.833111	0.0711389	0.010889	-0.02111	0.066	0.102389	-0.041361	0.0641389	0.071639	0.1382889	1.381125	0.211947	0.192327
	70	0	0.31899527	0.868998	0.766668	0.2392666	0.153112	0.561359	0.3686035	0.315631	0.03925425			
Conserved Element Deletions:	71	1140.778	111.472222	-37.1778	-114.528	-593.88889	-530.278	-537.5278	-531.2778	-475.778	-490.97778	70.70252	0	0.924186
	71	0	0.03252049	0.435511	0.028199	0	0	0	0	0	0			
TFBS Deletions:	72	51.66667	-9.6666667	0.333333	-5.16667	-22.222222	-24.4167	-21.41667	-18.41667	-15.4167	-25.866667	16.966	0	0.745234
	72	0	0.01616676	0.927234	0.191712	5.84E-10	3.48E-08	7.3E-07	1.334E-05	0.0002	9.48E-10			
TFBS Deletions/Total Deletions	73	0.006667	-0.0009167	0.000733	8.33E-05	0.0005556	-0.00017	0.000333	0.0013333	0.000833	-0.0004667	1.909118	0.062164	0.247644
	73	0	0.1792659	0.246235	0.902173	0.2981488	0.805843	0.623249	0.0524908	0.221575	0.45923559			
miRNA-BS disrupting Deletions:	74	2.222222	1.0277778	-0.62222	-0.72222	-1.6666667	-0.97222	-1.722222	-1.472222	-1.22222	-1.6222222	4.786588	5.33E-05	0.452137
	74	6.84E-09	0.09345159	0.271006	0.235999	0.0007869	0.112166	0.00578	0.0174479	0.047	0.00513293			
miRNA-BS disrupting Deletions/Total Deletions	75	0.035667	0.0090833	-0.01027	-0.00492	-0.0163333	0.004583	-0.021167	-0.017917	-0.00967	-0.0164667	1.250643	0.279681	0.17738
	75	9.44E-06	0.5005191	0.412403	0.715046	0.1252241	0.733586	0.119184	0.1860539	0.473513	0.19035978			
ESE-BS deletion Deletions:	76	5.666667	0.0833333	0.133333	2.333333	-3.4444444	-3.41667	-4.666667	-3.416667	-3.16667	-2.0666667	4.767494	5.58E-05	0.451147
	76	3.51E-09	0.9559547	0.924153	0.125312	0.0047506	0.026242	0.002792	0.0262417	0.038897	0.14323532			
ESE-BS deletion Deletions/Total Deletions	77	0.078111	-0.0061111	0.004489	0.028889	-0.0148889	-0.02811	-0.055361	-0.039111	-0.02661	0.0014889	2.385351	0.019118	0.291417
	77	1.46E-08	0.78048224	0.825425	0.190413	0.3879294	0.202473	0.013591	0.077841	0.227321	0.94166922			
ESE-BS induction Deletions:	78	6.333333	1.1666667	-2.13333	-0.58333	-3.8888889	-3.58333	-2.833333	-2.583333	-4.83333	-2.1333333	4.000981	0.000343	0.408223
	78	2.1E-11	0.4147641	0.110646	0.682882	0.0008671	0.014106	0.05034	0.0736707	0.00114	0.11064585			
ESE-BS induction Deletions/Total Deletions	79	0.085889	0.0091111	-0.02629	-0.00264	-0.0185556	-0.02014	-0.010139	-0.001889	-0.05289	0.0051111	1.261643	0.273372	0.178661
	79	5.97E-09	0.69612009	0.226991	0.909888	0.3121828	0.38903	0.663881	0.9354302	0.025988	0.81332981			
ESS-BS deletion Deletions:	80	8.555556	-1.3055556	0.244444	-2.55556	-4.5555556	-2.30556	-4.305556	-2.555556	-5.05556	-3.3555556	5.599958	8.5E-06	0.491226
	80	0	0.30698176	0.836128	0.047915	2.086E-05	0.073547	0.001158	0.047915	0.000168	0.00579523			
ESS-BS deletion Deletions/Total Deletions	81	0.119111	-0.0188611	-0.00291	-0.02261	-0.0153333	0.029139	-0.022861	0.0258889	-0.04561	-0.0123111	2.619824	0.010604	0.31115
	81	0	0.32807242	0.870363	0.241749	0.310935	0.132703	0.236637	0.1808306	0.020042	0.49082231			
ESS-BS induction Deletions:	82	5.333333	-0.8333333	-1.53333	-1.58333	-3.4444444	-2.83333	-3.583333	-3.583333	-3.58333	-3.3333333	4.615955	7.94E-05	0.443162
	82	0	0.42375938	0.115313	0.130924	6.988E-05	0.007943	0.000942	0.0009419	0.000942	0.00091958			
ESS-BS induction Deletions/Total Deletions	83	0.077333	-0.0110833	-0.02513	-0.01808	-0.029	-0.02058	-0.036083	-0.035083	-0.03933	-0.0343333	1.171048	0.328753	0.167987
	83	6.3E-10	0.56829422	0.165866	0.352867	0.0600825	0.290751	0.066309	0.0739929	0.045819	0.05993677			

Splicing Change Deletions:	84	16.88889	5.6111111	6.311111	-3.13889	-7.5555556	-5.88889	-8.138889	-7.138889	-6.88889	-7.0888889	8.855432	0	0.604242
	84	0	0.04527336	0.015971	0.257804	0.0008269	0.035898	0.004259	0.0115885	0.01469	0.00710883			
Splicing Change Deletions/Total Deletions	85	0.354556	0.0501944	0.069244	-0.03781	0.0006667	0.002944	-0.019556	0.0114444	0.000444	-0.0185556	0.733775	0.689781	0.112305
	85	0	0.35064279	0.167043	0.481451	0.9873508	0.956195	0.715382	0.8309637	0.993385	0.70933446			
Protein motif disrupting Deletions:	86	7.888889	2.6111111	1.111111	1.111111	-2.8888889	0.861111	0.361111	-1.138889	1.111111	-1.6888889	1.805275	0.079868	0.237372
	86	5.65E-10	0.18834516	0.544387	0.573591	0.0652322	0.66258	0.854724	0.5640714	0.573591	0.35769982			
Protein motif disrupting Deletions/Total Deletions	87	0.643111	0.1068889	0.111889	0.098889	0.2071111	0.152389	-0.007611	0.0593889	0.216889	0.1646889	1.648876	0.115657	0.221359
	87	0	0.28682914	0.230216	0.324139	0.009981	0.130556	0.939289	0.552827	0.032887	0.07924583			
Conserved Element Rearrangements:	88	306.8889	-10.888889	-22.6889	-27.1389	-128.11111	-103.389	-97.88889	-106.6389	-79.8889	-135.68889	22.62347	0	0.795943
	88	0	0.54020448	0.171537	0.129631	0	1.64E-07	5.6E-07	7.886E-08	2.61E-05	8E-12			
TFBS Rearrangements:	89	15.11111	-2.8611111	-2.51111	-4.11111	-5.5555556	-6.61111	-5.861111	-6.361111	-4.36111	-6.5111111	4.722053	0.000062	0.448777
	89	0	0.09867419	0.118027	0.018883	9.756E-05	0.00025	0.001036	0.0004054	0.012979	0.00011177			
TFBS Rearrangements/Total Rearrangements	90	0.005111	-0.0001111	0.000289	-0.00061	0.0017778	0.000139	0.000389	0.0001389	0.000889	0.0012889	1.473915	0.172656	0.20263
	90	0	0.91005971	0.751757	0.534975	0.0238167	0.887712	0.692726	0.8877118	0.36759	0.16108857			
miRNA-BS disrupting Rearrangements:	91	0.333333	0.1666667	0.466667	0.416667	0.2222222	-0.08333	-0.083333	-0.333333	0.166667	-0.1333333	0.945513	0.499625	0.140169
	91	0.110677	0.65534883	0.180764	0.266345	0.4487077	0.823304	0.823304	0.3730921	0.655349	0.70040484			
miRNA-BS disrupting Rearrangements/Total Rearrangements	92	0.032333	0.0201667	0.048267	0.056167	0.1491111	0.017667	-0.007333	-0.032333	0.048917	0.0342667	0.820012	0.610825	0.123869
	92	0.552478	0.83697425	0.596042	0.567005	0.0557292	0.856945	0.940347	0.7415342	0.617976	0.70651599			
ESE-BS deletion Rearrangements:	93	4.444444	-0.6944444	0.555556	-0.94444	-1.7777778	-1.44444	-0.444444	-1.944444	-1.94444	-2.8444444	2.437143	0.016788	0.295872
	93	7.6E-11	0.50553866	0.565905	0.365881	0.0324279	0.168418	0.669721	0.0652457	0.065246	0.00432402			
ESE-BS deletion Rearrangements/Total Rearrangements	94	0.111111	-0.0116111	0.014289	-0.00486	0.0065556	-0.01786	0.010889	-0.011611	-0.02561	-0.0287111	0.577618	0.825494	0.09057
	94	2.48E-09	0.69130117	0.598723	0.8679	0.7749744	0.541636	0.709582	0.6913012	0.382181	0.29184952			
ESE-BS induction Rearrangements:	95	3.555556	-0.8055556	-0.55556	-0.55556	-1.4444444	-1.30556	0.444444	-0.555556	-0.80556	-2.3555556	1.174153	0.326725	0.168358
	95	2.72E-07	0.47488953	0.595162	0.621811	0.1051639	0.248304	0.693021	0.6218108	0.47489	0.02682721			
ESE-BS induction Rearrangements/Total Rearrangements	96	0.086667	-0.0109167	-0.00207	0.001333	0.003	-0.02042	0.030833	0.0375833	0.001583	-0.0234667	0.740756	0.683404	0.113252
	96	4.09E-06	0.72699245	0.943207	0.965972	0.9026187	0.514268	0.325625	0.2316729	0.959597	0.41969584			
ESS-BS deletion Rearrangements:	97	2.111111	0.6388889	0.888889	-0.36111	-0.8888889	0.388889	-1.111111	-0.861111	-1.61111	-1.9111111	2.886231	0.005421	0.332277
	97	1.54E-05	0.43656216	0.244862	0.659629	0.1696835	0.635322	0.178007	0.2952417	0.052535	0.0140368			
ESS-BS deletion Rearrangements/Total Rearrangements	98	0.099556	0.0364444	0.023044	-0.00631	-0.0153333	0.050944	-0.036556	0.0021944	-0.05606	-0.0813556	1.990993	0.050909	0.255551
	98	4.48E-05	0.37845753	0.547887	0.878543	0.6359644	0.219533	0.377014	0.9575831	0.177223	0.03664891			
ESS-BS induction Rearrangements:	99	1.777778	0.2222222	-0.37778	-0.27778	-0.4444444	-0.27778	-1.527778	-0.277778	-0.52778	-0.9777778	0.828091	0.603467	0.124937
	99	0.000103	0.77550711	0.601715	0.721532	0.4679306	0.721532	0.053146	0.7215317	0.498823	0.1792308			
ESS-BS induction Rearrangements/Total Rearrangements	100	0.071778	0.0277222	-0.00978	0.012972	0.0491111	0.002222	-0.061028	0.0419722	0.053722	0.0124222	0.866678	0.568613	0.130001
	100	0.017408	0.6031106	0.843247	0.807627	0.2422751	0.966722	0.254221	0.4317713	0.315009	0.80165979			
Splicing Change Rearrangements:	101	2.666667	-0.1666667	-1.66667	-0.91667	-1	-0.41667	2.583333	-0.916667	-1.16667	-2.2666667	3.380326	0.001574	0.368214
	101	8.93E-06	0.86807379	0.076933	0.362384	0.2065914	0.678114	0.011935	0.3623842	0.247264	0.01720119			
Splicing Change Rearrangements/Total Rearrangements	102	0.304333	-0.0503333	-0.14713	-0.13608	-0.0728889	-0.05008	0.163917	-0.063833	-0.11258	-0.1977333	1.771962	0.08649	0.234016
	102	2.35E-06	0.6371952	0.140368	0.204631	0.3849138	0.638866	0.127558	0.5499627	0.29307	0.04897962			
Protein motif disrupting Rearrangements:	103	8.555556	-1.3055556	0.044444	-3.05556	-3.4444444	-0.55556	-3.305556	-4.305556	-2.30556	-4.7555556	2.81623	0.006466	0.326852
	103	0	0.43336789	0.977024	0.069503	0.0100109	0.738373	0.050043	0.0114785	0.168555	0.00288732			
Protein motif disrupting Rearrangements/Total Rearrangements	104	0.774222	0.0100278	-0.01322	-0.16622	-0.0897778	-0.00322	-0.041472	-0.122972	-0.10122	-0.1616222	0.98564	0.46606	0.145254
	104	0	0.92153413	0.888721	0.105913	0.2631682	0.974749	0.683907	0.2295798	0.321849	0.09062251			
Nonsense SNPs:	105	14.22222	4.5277778	-0.22222	-2.72222	-5	-4.22222	-1.972222	-2.972222	-4.22222	-2.0222222	2.765212	0.007352	0.322842
	105	0	0.07543307	0.924212	0.28149	0.013337	0.096838	0.43429	0.2400316	0.096838	0.3879827			
Nonsense SNPs/Total SNPs	106	0.009444	0.0018056	0.000356	-0.00019	0.0035556	0.003056	0.004056	0.0028056	0.000556	0.0063556	1.909417	0.062119	0.247673
	106	7.55E-10	0.4497901	0.872353	0.934994	0.060629	0.202687	0.092346	0.2416737	0.815765	0.00529016			
Frameshift Structural Variants:	107	51.66667	2.0833333	4.333333	0.833333	-18.444444	1.833333	-5.666667	-11.66667	-1.16667	-3.0666667	5.665148	7.3E-06	0.494119
	107	0	0.68786315	0.36912	0.872263	2.285E-05	0.723643	0.276339	0.0271019	0.821919	0.52441496			
Frameshift Structural Variants/Total Variants	108	0.522667	0.0590833	0.027733	0.028833	0.047	0.100583	0.043583	0.0143333	0.045333	0.1181333	3.232311	0.002276	0.357861
	108	0	0.06533956	0.346777	0.363803	0.0617399	0.002167	0.171525	0.6509092	0.155197	0.00014202			
Frameshift Insertions:	109	21.44444	3.3055556	0.955556	1.805556	-7.2222222	2.805556	1.305556	-2.444444	-0.19444	3.5555556	2.917767	0.005008	0.334692
	109	0	0.32537744	0.758617	0.590192	0.0074604	0.403406	0.696817	0.4663288	0.9537	0.2550042			
Frameshift Insertions/Total Insertions	110	0.623636	0.1874074	0.096072	0.052886	0.083606	0.144775	0.109321	0.1321184	0.067585	0.2108318	2.582314	0.011654	0.308067
	110	0	0.00443613	0.108531	0.408876	0.0986303	0.026106	0.090425	0.0417155	0.292017	0.00066709			
Frameshift Deletions:	111	25.77778	-0.5277778	1.022222	-1.77778	-9.8888889	-4.52778	-7.027778	-7.277778	-0.27778	-4.9777778	4.631727	7.65E-05	0.444004
	111	0	0.85672703	0.706486	0.543603	5.12E-05	0.124695	0.018548	0.0149049	0.924291	0.06995804			
Frameshift Deletions/Total Deletions	112	0.697082	-0.0140659	-0.0151	-0.04951	0.0232184	0.051088	-0.041832	-0.060248	0.001296	0.0235217	1.003017	0.451907	0.147437
	112	0	0.77987039	0.746523	0.326816	0.5568591	0.311769	0.406937	0.2336197	0.979456	0.6148238			
Frameshift Rearrangements:	113	4.444444	-0.6944444	2.355556	0.805556	-1.3333333	3.555556	0.055556	-1.944444	-0.69444	-1.6444444	5.165249	2.24E-05	0.471056
	113	7.3E-10	0.53602888	0.026227	0.473074	0.1326017	0.002199	0.960459	0.0861499	0.536029	0.11723499			
Frameshift Rearrangements/Total Rearrangements	114	0.171691	-0.0232244	0.038996	0.055675	0.0244762	0.137634	0.032942	-0.053922	-0.01015	-0.005471	2.724155	0.008153	0.319581
	114	8.59E-10	0.5940419	0.336123	0.20361	0.4743384	0.002273	0.450131	0.2180405	0.815618	0.89229119			
Splicing Change Variants:	115	46.66667	2.0833333	3.933333	-10.4167	-19.555556	-14.1667	-14.41667	-18.66667	-16.9167	-20.266667	10.92459	0	0.653205
	115	0	0.64461457	0.34933	0.023583	5.393E-07	0.002434	0.002061	9.536E-05	0.000356	7.5099E-06			
Splicing Change Variants/Total Variants	116	0.119333	-0.0045833	0.013067	-0.01083	0.0233333	0.033417	0.017167	0.0089167	-0.00033	0.0084667	1.823061	0.076531	0.239151
	116	0	0.75708816	0.343553	0.465458	0.0478636	0.026792	0.248857	0.5477454	0.982047	0.53860412			
Probably Damaging nscSNPs:	117	274.4444	46.0555556	-1.64444	-53.6944	-107.88889	-94.4444	-69.69444	-72.69444	-74.6944	-96.644444	17.77639	0	0.753991
	117	0	0.01682303	0.925137	0.005666	4.01E-10	3.94E-06	0.000423	0.0002491	0.000174	5.4165E-07			
Probably Damaging nscSNPs/Total nscSNPs	118	0.319	0.01225	0.0146	-0.026	0.0605556	0.0325	0.03575	0.03875	0.00625	0.0512	7.688728	1E-07	0.570011
	118	0	0.4181701	0.299311	0.088413	2.64E-06	0.034253	0.020297	0.0121774	0.679011	0.00048502			
Possibly Damaging nscSNPs:	119	154.2222	22.5277778	-5.02222	-31.2222	-73.444444	-63.9722	-53.22222	-50.72222	-64.2222	-69.422222	23.10411	0	0.799337
	119	0	0.04535199	0.625848	0.006196	3E-12	2.04E-07	8.65E-06	1.99E-05	1.86E-07	3.883E-09			
Possibly Damaging nscSNPs/Total nscSNPs	120	0.179111	0.0033889	0.003289	-0.01536	0.0047778	-0.00261	-0.003611	0.0051389	-0.03311	-0.0035111	3.414424	0.001446	0.370552
	120	0	0.70626958	0.693561	0.090861	0.4987183	0.771481	0.688012	0.5679391	0.00044	0.67403567			
Protein motif damaging Variants:	121	110.5556	21.4444444	-1.95556	-0.80556	-44.111111	-23.0556	-22.55556	-26.30556	-17.0556	-36.155556	12.66235	0	0.685847
	121	0	0.01771499	0.811919	0.927495	1.948E-08	0.011035	0.012811	0.0039816	0.057351	3.7442E-05			
Protein motif damaging Variants/Total Variants	122	0.327	0.0165	0.0022	0.03425	0.024	0.05325	0.0235	0.02075	0.032	0.0296	1.436777	0.187553	0.198538
	122	0	0.41460115	0.90648	0.093008	0.1326781	0.01005	0.246468	0.3056093	0.116071	0.11730718			
TFBS Disrupting Variants:	123	86.22222	-17.222222	-5.82222	-14.9722	-38.555556	-36.9722	-34.97222	-33.72222	-26.9722	-43.222222	25.64773	0	0.815567
	123	0	0.00091455	0.210647	0.003608	0	2.36E-10	1.25E-09	3.523E-09	8.27E-07	0			
TFBS Disrupting Variants/Total Variants	124	0.002	-0.001	0	-0.00025	0.0001111	0	0	0	0	0	14.34498	0	0.712087
	124	0	4E-12	1	0.03895	0.2369988	1	1	1	1	1			
miRNA-BS Disrupting Variants:	125	23.44444	3.0555556	0.155556	-0.94444	-11.666667	-9.94444	-12.44444	-8.694444	-6.69444	-12.644444	11.68286	0	0.668247
	125	0	0.26246674	0.950761	0.727959	6.402E-07	0.00047	1.91E-05	0.0020046	0.015822	3.7879E-06			
miRNA-BS Disrupting Variants/Total Variants	126	0.054111	-0.0026111	0.001489	0.006139	0.008	0.006139	-0.010611	0.0046389	0.009889	-0.0009111	1.367565	0.218283	0.190799
	126	0	0.72745207	0.830419	0.413527	0.1762288	0.413527	0.159591	0.5362021	0.189523	0.89572013			
ESE-BS Disrupting Variants:	127	145	9	-8.8	-11.5	-72.555556	-62.5	-52	-56	-57.5	-71.6	20.00421	0	0.77523
	127	0	0.42891623	0.404792	0.312836	1.1E-11	5.77E-07	1.94E-05	5.244E-06	3.18E-06	3.157E-09			
ESE-BS Disrupting Variants/Total Variants	128	0.124889	-0.0061389	-0.00249	0.002861	0.004	0.002861	0.002611	-0.000889	-0.01339	-0.0058889	1.193945	0.314018	0.170711
	128	0	0.3996077	0.712339	0.694028	0.4837914	0.694028	0.719554	0.9026741	0.068886	0.384096			
ESS-BS Disrupting Variants:	129	64.22222	11.5277778	-4.02222	-11.2222	-35.111111	-26.7222	-29.72222	-30.72222	-31.9722	-35.422222	26.34306	0	0.819557
	129	0	0.02816927	0.402015	0.032458	1E-12	2.04E-06	2.09E-07	9.638E-08	3.62E-08	2.59E-10			
ESS-BS Disrupting Variants/Total Variants	130	0.133	0.00875	-0.0034	-0.00725	-0.0065556	0.00425	-0.01475	-0.01675	-0.03125	-0.0182	1.909714	0.062074	0.247702
	130	0	0.47135891	0.762642	0.550401	0.4914438	0.726064	0.226333	0.1701432	0.011857	0.10923002			
**Total Likely Functional Novel Variants:**	131	798.7778	66.2222222	-22.1778	**-125.028**	***-360.5556***	***-290.778***	***-251.028***	***-263.7778***	***-250.028***	***-331.77778***	30.3061	0	***0.83936***
	131	0	0.14019574	0.591949	0.00634	0	9.42E-09	3.44E-07	1.101E-07	3.76E-07	1.9E-11			
**Total Genotypes (not just novel)**	132	6042271	***-83419.25***	**-57488**	**-51149.3**	***-395516.3***	***-375283***	***-428364***	***-442378***	***-332702***	***-444921.8***	222.3361	0	***0.97458***
	132	0	8.1932E-05	0.002709	0.012324	0	0	0	0	0	0			
synonymous to nonsynonymous rate	133	0.732889	-0.0042172	-0.00782	0.006695	-0.140776	-0.18184	-0.14756	-0.129663	-0.12524	-0.1533821	17.80214	0	0.75426
	133	0	0.87937151	0.761941	0.809639	1.261E-08	8.87E-09	1.24E-06	1.42E-05	2.54E-05	1.0017E-07			
Functional Variant/Total Variants	134	0.000132	0.000013	-2.5E-06	-2E-05	-0.0000546	-4.3E-05	-3.46E-05	-3.67E-05	-3.6E-05	-0.0000488	24.71877	0	0.809953
	134	0	0.08539274	0.719894	0.009621	0	2.53E-07	1.5E-05	5.413E-06	7.26E-06	1.069E-09			
Functional Variant - Prob Damaging/Total Variants	135	0.000107	0.0000088	-1.9E-06	-1.5E-05	-0.0000434	-3.3E-05	-2.71E-05	-2.96E-05	-2.6E-05	-0.0000384	24.50379	0	0.808605
	135	0	0.13457449	0.729554	0.013507	0	3.43E-07	1.59E-05	3.137E-06	2.56E-05	9.81E-10			
Functional Nonsense SNPs/Total Variants	136	2.4E-06	0.0000008	0	-4E-07	-0.0000007	-6E-07	-2E-07	-3E-07	-6E-07	-0.0000002	2.256718	0.026376	0.280104
	136	0	0.07013828	0.957389	0.312888	0.0359211	0.173851	0.684173	0.4199913	0.163657	0.65926819			
Functional Frameshift Structural Variants/Total Variants	137	8.5E-06	0.0000005	8E-07	2E-07	-0.0000027	9E-07	-4E-07	-1.4E-06	3E-07	0.0000001	4.675618	6.91E-05	0.446333
	137	0	0.59764303	0.335644	0.810803	0.0002727	0.316847	0.690742	0.1165748	0.742183	0.87265161			
Functional Insertions/Total Variants	138	3.5E-06	0.0000006	2E-07	3E-07	-0.000001	7E-07	5E-07	-2E-07	2E-07	0.0000009	2.82991	0.006247	0.327919
	138	0	0.29535879	0.725355	0.562756	0.0256204	0.206049	0.38103	0.7917645	0.760723	0.08976376			
Functional Deletions/Total Variants	139	4.3E-06	0	2E-07	-3E-07	-0.0000015	-5E-07	-9E-07	-0.000001	2E-07	-0.0000006	3.47615	0.001241	0.374741
	139	0	0.95204227	0.653979	0.603217	0.0004544	0.305713	0.069471	0.0586634	0.695726	0.2410774			
Functional Rearrangements/Total Variants	140	7E-07	-0.0000001	4E-07	1E-07	-0.0000002	7E-07	1E-07	-3E-07	-1E-07	-0.0000002	5.035655	3.01E-05	0.46473
	140	2.03E-09	0.57777222	0.028243	0.466233	0.221296	0.000752	0.734153	0.1359658	0.674512	0.18876176			
Functional Splicing Change Variants/Total Variants	141	7.7E-06	0.0000005	7E-07	-1.7E-06	-0.0000029	-2E-06	-0.000002	-2.7E-06	-2.5E-06	-0.000003	8.653638	0	0.598717
	141	0	0.55384821	0.309089	0.032553	7.37E-06	0.011625	0.012014	0.0007064	0.001682	7.4303E-05			
Probably Damaging nscSNPs/Total Variants	142	4.54E-05	0.0000084	1E-07	-8.6E-06	-0.0000159	-1.4E-05	-0.000009	-9.4E-06	-1E-05	-0.0000137	14.07348	0	0.708154
	142	0	0.01058484	0.962003	0.008614	1.803E-08	5.64E-05	0.006304	0.0042633	0.001608	1.6866E-05			
Possibly Damaging nscSNPs/Total Variants	143	2.55E-05	0.0000041	-6E-07	-5E-06	-0.0000112	-9.6E-06	-7.5E-06	-0.000007	-9.8E-06	-0.0000104	19.4933	0	0.77069
	143	0	0.02906017	0.726866	0.008802	7.7E-11	2.2E-06	0.000128	0.0003116	1.58E-06	7.7238E-08			
Functional Protein motif damaging Variants/Total Variants	144	1.83E-05	0.0000039	-2E-07	0	-0.0000065	-2.8E-06	-2.6E-06	-3.2E-06	-1.9E-06	-0.000005	9.952207	0	0.631798
	144	0	0.01189989	0.913583	0.986609	5E-07	0.061267	0.084622	0.0333605	0.204488	0.00059479			
Functional TFBS Disrupting Variants/Total Variants	145	1.43E-05	-0.0000027	-8E-07	-2.4E-06	-0.0000058	-5.6E-06	-5.1E-06	-4.9E-06	-3.9E-06	-0.0000066	20.061	0	0.775724
	145	0	0.00215602	0.288146	0.006247	1E-12	7.09E-09	5.94E-08	1.9E-07	1.78E-05	4E-12			
Functional miRNA-BS Disrupting Variants/Total Variants	146	3.9E-06	0.0000006	1E-07	-1E-07	-0.0000018	-1.5E-06	-1.9E-06	-1.2E-06	-9E-07	-0.000002	9.85469	0	0.629504
	146	0	0.22473586	0.883981	0.78212	4.702E-06	0.001743	8.54E-05	0.0084046	0.042711	2.1016E-05			
Functional ESE-BS Disrupting Variants/Total Variants	147	0.000024	0.0000018	-1.2E-06	-1.7E-06	-0.0000112	-9.4E-06	-7.4E-06	-8.1E-06	-8.7E-06	-0.0000109	16.93282	0	0.744862
	147	0	0.33135946	0.479109	0.366977	1.66E-10	4.49E-06	0.000204	5.928E-05	2.02E-05	3.9059E-08			
Functional ESS-BS Disrupting Variants/Total Variants	148	1.06E-05	0.0000021	-6E-07	-1.8E-06	-0.0000055	-4E-06	-4.5E-06	-4.6E-06	-5E-06	-0.0000055	22.44691	0	0.794668
	148	0	0.02003153	0.489613	0.045426	2.7E-11	2.07E-05	2.74E-06	1.341E-06	3.01E-07	4.219E-09			
Functional Variant/Total Novel Variants	149	0.003464	-0.0000077	0.000158	-0.00018	0.0010717	0.001236	0.001094	0.0011067	0.000699	0.0009826	45.16012	0	0.886186
	149	0	0.9517535	0.182041	0.164282	0	0	2E-12	1E-12	5.79E-07	5E-12			
Functional Variant - Prob Damaging/Total Novel Variants	150	0.002795	-0.0000448	0.000132	-9.9E-05	0.0009037	0.00107	0.000923	0.0008914	0.000686	0.0008448	51.10283	0	0.898072
	150	0	0.65758859	0.163624	0.328744	0	0	0	1E-12	3.22E-09	0			
Functional Nonsense SNPs/Total Novel Variants	151	0.002795	-0.0000448	0.000132	-9.9E-05	0.0009037	0.00107	0.000923	0.0008914	0.000686	0.0008448	51.10283	0	0.898072
	151	0	0.65758859	0.163624	0.328744	0	0	0	1E-12	3.22E-09	0			
Functional Frameshift Structural Variants/Total Novel Variants	152	0.000224	-0.0000091	3.69E-05	4.77E-05	0.0001191	0.00027	0.000159	0.000118	0.000159	0.0002377	15.268	0	0.724701
	152	0	0.80471734	0.281311	0.196736	9.658E-05	3.18E-10	4.82E-05	0.0019609	4.92E-05	1.559E-09			
Functional Insertions/Total Novel Variants	153	9.27E-05	0.0000061	1.08E-05	3.18E-05	0.0000538	0.000131	9.64E-05	0.0000698	6.77E-05	0.0001446	9.184081	0	0.612923
	153	9.63E-09	0.81090433	0.649435	0.217571	0.009122	2.87E-06	0.000339	0.008003	0.00996	5.8876E-08			
Functional Deletions/Total Novel Variants	154	0.000112	-0.0000109	1.38E-05	7.4E-06	0.0000525	0.000085	4.45E-05	0.0000461	8.19E-05	0.0000857	7.097712	4E-07	0.550308
	154	0	0.59603418	0.468488	0.717237	0.0016686	9.35E-05	0.033097	0.0273744	0.000157	2.597E-05			
Functional Rearrangements/Total Novel Variants	155	1.93E-05	-0.0000043	1.23E-05	8.5E-06	0.0000128	5.47E-05	1.82E-05	0.0000021	9.3E-06	0.0000074	8.4993	0	0.594386
	155	3.15E-05	0.57976763	0.094381	0.276813	0.0392678	1.36E-09	0.022142	0.7849992	0.235581	0.31142547			
Functional Splicing Change Variants/Total Novel Variants	156	0.000203	-0.0000077	0.000033	-2.4E-05	0.0000762	9.97E-05	6.62E-05	0.0000367	2.37E-05	0.0000482	3.777415	0.000591	0.394409
	156	0	0.80053378	0.244526	0.43909	0.0020216	0.00157	0.032245	0.2293734	0.435808	0.09086097			
Probably Damaging nscSNPs/Total Novel Variants	157	0.001191	0.0000897	0.000082	-0.00013	0.0005339	0.000474	0.000513	0.0005324	0.000325	0.0005037	25.49715	0	0.81468
	157	0	0.2600823	0.267092	0.095466	2E-12	8.94E-08	1.19E-08	4.37E-09	0.000108	2.559E-09			
Possibly Damaging nscSNPs/Total Novel Variants	158	0.000669	0.0000371	2.69E-05	-7.9E-05	0.000168	0.000166	0.000172	0.0002153	1.38E-05	0.0001379	10.50564	0	0.644295
	158	0	0.40851186	0.517451	0.080916	9.184E-06	0.000422	0.000261	8.348E-06	0.757185	0.001414			
Functional Protein motif damaging Variants/Total Novel Variants	159	0.000479	0.0000489	2.69E-05	6.26E-05	0.0002099	0.00033	0.000254	0.0002411	0.000231	0.0002295	11.83673	0	0.671141
	159	0	0.35179803	0.580058	0.234454	2.668E-06	2.39E-08	7.1E-06	1.777E-05	3.63E-05	1.1525E-05			
Functional TFBS Disrupting Variants/Total Novel Variants	160	0.000374	-0.000098	1.9E-06	-1.6E-05	0.0001192	8.42E-05	5.22E-05	0.000075	7.47E-05	0.0000368	8.722081	0	0.600608
	160	0	0.00360017	0.950608	0.632772	1.454E-05	0.011666	0.112996	0.0240543	0.024694	0.22703978			
Functional miRNA-BS Disrupting Variants/Total Novel Variants	161	0.000102	0.0000039	8.3E-06	7.8E-06	0.0000205	2.35E-05	-1.04E-05	0.0000246	2.46E-05	0.0000014	1.45506	0.180085	0.200558
	161	0	0.79480167	0.551426	0.604573	0.0858222	0.121024	0.491015	0.1045546	0.105017	0.92179917			
Functional ESE-BS Disrupting Variants/Total Novel Variants	162	0.000628	-0.0000131	0.000006	1.53E-05	0.0001211	0.000134	0.000145	0.0001322	3.53E-05	0.00007	4.262891	0.000183	0.423625
	162	0	0.77918463	0.890451	0.742373	0.0014325	0.005344	0.002582	0.0058316	0.450009	0.10870015			
Functional ESS-BS Disrupting Variants/Total Novel Variants	163	0.000279	0.0000244	0.000003	-2.4E-05	0.0000245	6.75E-05	8.9E-06	0.000008	-3.4E-05	-0.0000044	1.679153	0.107741	0.224511
	163	0	0.41699689	0.915271	0.422305	0.3013468	0.027476	0.767939	0.7894632	0.267023	0.87434201			
