# Supplementary material for: Clinical Implications of Human Population Differences in Genome-Wide Rates of Functional Genotypes
Source: Front Genet. 2012 Nov 1;3:211. doi: 10.3389/fgene.2012.00211 (PMC3485509; doi:10.3389/fgene.2012.00211)
Supplement: Supplementary Data Sheet S6 — Regression analysis results for ancestral allele-based population-specific variants: all variants. [file 32001_Schork_DataSheet6.PDF]

Ancestral Allele Based Variants: All Population Specific Variants
Row 1: Regression Coefficients
Row 2: P-values for Regression Coefficients

	Var Cat	Y-int	ASN	EUR	Overall F	p-val	R-Sqr
Coding SNPs:	1	5361.824	-3767.8235	-3736.747	173.5863	0	0.967668
	1	0	0	0			
Nonsynonymous SNPs:	2	2797	-1842.25	-1828.538	170.346	0	0.967073
	2	0	0	0			
Synonymous SNPs:	3	2528.765	-1906.0147	-1890.611	167.9309	0	0.966615
	3	0	0	0			
Nonsense SNPs:	4	36.05882	-19.558824	-17.59729	13.78554	0	0.703863
	4	0	3.18E-10	7.6E-11			
Untranslated Region SNPs:	5	7760.941	-5864.3162	-5778.403	171.4104	0	0.967271
	5	0	0	0			
Non-coding RNA SNPs:	6	212706.1	-164812.06	-161946.8	191.1975	0	0.970558
	6	0	0	0			
Intronic SNPs:	7	314429.7	-243556.46	-240293.9	183.8867	0	0.969423
	7	0	0	0			
Intergenic SNPs:	8	424185.7	-331484.96	-325862.2	187.4808	0	0.969992
	8	0	0	0			
**Total SNPs:**	9	753209.8	-585807.14	-576772.5	186.1326	0	0.969781
	9	0	0	0			
Coding Insertions:	10	60.35294	-26.227941	-28.66063	16.20064	0	0.736371
	10	0	9.99E-10	0			
In-frame Insertions:	11	6.882353	-3.0073529	-3.959276	2.815865	0.006472	0.326823
	11	0	0.0122153	0.000193			
Out-of-frame Insertions:	12	14.94118	-8.1911765	-7.171946	4.565225	8.94E-05	0.440437
	12	0	5.538E-05	4.15E-05			
Frameshift Insertions:	13	38.52941	-15.029412	-17.52941	15.71517	0	0.730423
	13	0	5.672E-09	0			
Untranslated region Insertions:	14	574.8235	-345.44853	-350.4389	97.84908	0	0.944042
	14	0	0	0			
Non-coding RNA Insertions:	15	13816.71	-8468.4559	-8308.629	81.66325	0	0.933686
	15	0	0	0			
Intronic Insertions:	16	21747.12	-13324.118	-13046.89	77.74424	0	0.930576
	16	0	0	0			
Intergenic Insertions:	17	27430	-17072.125	-16772.62	84.64839	0	0.935875
	17	0	0	0			
**Total Insertions:**	18	49889.53	-30821.779	-30248.3	82.22416	0	0.934109
	18	0	0	0			
Coding Deletions:	19	92.41176	-51.911765	-53.95023	57.07706	0	0.907757
	19	0	0	0			
In-frame Deletions:	20	11.11765	-6.6176471	-6.040724	9.809492	0	0.628431
	20	0	4.395E-08	9.24E-09			
Inter-Codon Deletions:	21	19.52941	-8.1544118	-12.52941	15.43762	0	0.7269
	21	0	1.438E-06	0			
Frameshift Deletions:	22	61.76471	-37.139706	-35.38009	37.2791	0	0.865364
	22	0	0	0			
Untranslated region Deletions:	23	796.4706	-554.84559	-537.9321	129.2766	0	0.957061
	23	0	0	0			
Non-coding RNA Deletions:	24	20082.76	-13796.765	-13312.07	144.1047	0	0.961309
	24	0	0	0			
Intronic Deletions:	25	30387.94	-20811.191	-20176.94	128.0117	0	0.956656
	25	0	0	0			
Intergenic Deletions:	26	39845.24	-27517.86	-26558.62	134.8703	0	0.958769
	26	0	0	0			
**Total Deletions:**	27	71221.35	-49005.728	-47399.2	132.5182	0	0.958068
	27	0	0	0			
Coding rearrangements:	28	66.41176	-36.036765	-35.56561	28.01169	0	0.828462
	28	0	0	0			
In-frame rearrangements:	29	58.64706	-32.897059	-32.72398	28.71471	0	0.831956
	29	0	0	0			
Frameshift rearrangements:	30	7.764706	-3.1397059	-2.841629	2.045756	0.044504	0.260747
	30	0	0.0060861	0.00397			
Untranslated region rearrangements:	31	189.4706	-122.22059	-123.8552	87.08658	0	0.937558
	31	0	0	0			
Non-coding RNA rearrangements:	32	6290.706	-4121.8309	-4059.09	97.68841	0	0.943955
	32	0	0	0			
Intronic rearrangements:	33	8849.529	-6008.6544	-5955.76	111.4194	0	0.95052
	33	0	0	0			
Intergenic rearrangements:	34	12619.06	-8448.3088	-8355.597	106.435	0	0.948323
	34	0	0	0			
**Total rearrangements:**	35	21764.12	-14641.618	-14495.19	109.2956	0	0.949607
	35	0	0	0			
**Total number of variants:**	36	896084.8	-680276.26	-668915.1	180.4187	0	0.968854
	36	0	0	0			
Conserved Element SNPs:	37	43556.24	-32675.11	-32236.54	195.9607	0	0.971253
	37	0	0	0			
TFBS SNPs:	38	12.58824	-7.8382353	-7.20362	8.453205	0	0.593074
	38	0	2.803E-07	5.77E-08			
TFBS SNPs/Total SNPs	39	0	0	0	0	1	0
	39						
miRNA-BS disrupting SNPs:	40	72.82353	-51.573529	-48.97738	55.20778	0	0.90493
	40	0	0	0			
miRNA-BS disrupting SNPs/Total SNPs	41	0.057588	0.0017868	0.003566	0.190702	0.996315	0.031833
	41	0	0.6462607	0.288172			
ESE-BS deletion SNPs:	42	204.1176	-144.61765	-140.1176	135.4	0	0.958924
	42	0	0	0			
ESE-BS deletion SNPs/Total SNPs:	43	0.055294	-0.0009191	0.002321	0.355141	0.960645	0.057698
	43	0	0.6940392	0.249716			
ESE-BS induction SNPs:	44	225.7647	-151.01471	-150.1493	96.44461	0	0.943273
	44	0	0	0			
ESE-BS induction SNPs/Total SNPs	45	0.061235	0.0072647	0.006611	3.00728	0.003999	0.341454
	45	0	0.0010548	0.000555			
ESS-BS deletion SNPs:	46	64.17647	-45.051471	-42.25339	114.3383	0	0.951722
	46	0	0	0			
ESS-BS deletion SNPs/Total SNPs	47	0.047353	0.0020221	0.00557	0.679679	0.738671	0.104894
	47	0	0.5305496	0.047285			
ESS-BS induction SNPs:	48	88.82353	-57.198529	-61.74661	99.65624	0	0.945001
	48	0	0	0			
ESS-BS induction SNPs/Total SNPs	49	0.065706	0.0155441	-0.000629	2.507109	0.014081	0.301803
	49	0	0.0007227	0.867958			
Splicing Change SNPs:	50	24.29412	-21.544118	-17.52489	22.87364	0	0.797724
	50	0	0	0			
Splicing Change SNPs/Total SNPs	51	0.021824	-0.0120735	0.001715	3.63664	0.000835	0.385374
	51	0	0.0001338	0.50516			
Protein Motif disrupting SNPs:	52	250.1765	-145.17647	-147.5611	115.7993	0	0.952302
	52	0	0	0			
Protein Motif disrupting SNPs/Total SNPs	53	0.267941	0.0279338	0.026443	2.652714	0.009761	0.31383
	53	0	0.0024691	0.000928			
Conserved Element Insertions:	54	2037.882	-1257.5074	-1247.652	113.0893	0	0.951215
	54	0	0	0			
TFBS Insertions:	55	19.82353	-12.698529	-11.0543	12.61152	0	0.68498
	55	0	7.22E-10	4.91E-10			
TFBS Insertions/Total Insertions	56	0.002529	0.0000956	0.000548	0.711555	0.709999	0.109276
	56	0	0.7633377	0.047905			
miRNA-BS disrupting Insertions:	57	1.470588	-1.0955882	-0.701358	2.387649	0.019008	0.291616
	57	5E-12	0.0007748	0.010748			
miRNA-BS disrupting Insertions/Total Insertions	58	0.011235	-0.0047353	0.003149	0.680923	0.737562	0.105066
	58	1.15E-06	0.2065301	0.327109			
ESE-BS deletion Insertions:	59	0.470588	-0.2205882	-0.316742	0.481667	0.89522	0.076678
	59	0.000385	0.3250946	0.10231			
ESE-BS deletion Insertions/Total Insertions	60	0.012	0.004625	-0.006154	0.248765	0.989329	0.041127
	60	0.017125	0.5957626	0.412101			
ESE-BS induction Insertions:	61	0.294118	-0.1691176	0.013575	0.157481	0.998349	0.026434
	61	0.009291	0.3867183	0.935381			
ESE-BS induction Insertions/Total Insertions	62	0.007706	-0.0014559	0.00591	0.199453	0.99557	0.033245
	62	0.070307	0.8447567	0.356478			
ESS-BS deletion Insertions:	63	0.117647	0.0073529	-0.117647	0.271251	0.985143	0.044678
	63	0.081903	0.9503922	0.24907			
ESS-BS deletion Insertions/Total Insertions	64	0.005588	0.0040368	-0.005588	0.311718	0.975159	0.051003
	64	0.159509	0.5629905	0.352415			
ESS-BS induction Insertions:	65	0.352941	-0.3529412	-0.352941	1.748325	0.091497	0.231618
	65	4.44E-05	0.0159963	0.005392			
ESS-BS induction Insertions/Total Insertions	66	0.016824	-0.0168235	-0.016824	1.667182	0.110809	0.223268
	66	6.38E-05	0.0185353	0.006512			
Splicing Change Insertions:	67	58.17647	-34.301471	-34.79186	29.1018	0	0.83382
	67	0	0	0			
Splicing Change Insertions/Total Insertions	68	0.458588	-0.0228382	-0.014973	0.144201	0.998869	0.024259
	68	0	0.3929605	0.514094			
Protein motif disrupting Insertions:	69	16.47059	-6.8455882	-6.778281	6.199944	2.3E-06	0.516664
	69	0	1.427E-05	9.62E-07			
Protein motif disrupting Insertions/Total Insertions	70	0.809824	0.0268015	0.085407	0.955184	0.491428	0.1414
	70	0	0.5220969	0.019852			
Conserved Element Deletions:	71	3320.471	-2305.4706	-2256.932	171.6236	0	0.96731
	71	0	0	0			
TFBS Deletions:	72	130.9412	-79.191177	-82.71041	45.92955	0	0.887878
	72	0	0	0			
TFBS Deletions/Total Deletions	73	0.006706	0.0010441	-9.05E-05	1.797916	0.081289	0.236633
	73	0	0.0045731	0.768172			
miRNA-BS disrupting Deletions:	74	5.941177	-4.9411765	-3.941177	8.799565	0	0.602728
	74	0	3.222E-08	1.95E-07			
miRNA-BS disrupting Deletions/Total Deletions	75	0.034765	-0.0178897	0.000389	1.697357	0.103225	0.226394
	75	0	0.0044786	0.940864			
ESE-BS deletion Deletions:	76	17.17647	-13.176471	-12.9457	26.54389	0	0.820677
	76	0	0	0			
ESE-BS deletion Deletions/Total Deletions	77	0.082588	-0.0300882	-0.022819	1.579521	0.13583	0.214041
	77	0	0.0086405	0.01978			
ESE-BS induction Deletions:	78	18.29412	-13.669118	-13.29412	25.40559	0	0.814136
	78	0	0	0			
ESE-BS induction Deletions/Total Deletions	79	0.087235	-0.0212353	-0.016466	1.134438	0.353354	0.163595
	79	0	0.0262956	0.044261			
ESS-BS deletion Deletions:	80	20.70588	-13.205882	-11.78281	15.19791	0	0.723782
	80	0	6E-11	1.7E-11			
ESS-BS deletion Deletions/Total Deletions	81	0.109529	0.0022206	0.020394	0.624949	0.786429	0.097269
	81	0	0.8623692	0.067296			
ESS-BS induction Deletions:	82	12.17647	-9.5514706	-9.330317	18.15917	0	0.757922
	82	0	1.7E-11	0			

Europeans relative to Asians

Var Cat	Y-int	EUR	Overall F	p-val	R-Sqr
1	1594	31.07692	0.688374	0.730898	0.106094
1	0	0.13788			
2	954.75	13.71154	0.231505	0.991944	0.038383
2	0	0.386946			
3	622.75	15.40385	0.515034	0.872661	0.081557
3	0	0.198426			
4	16.5	1.961539	0.309376	0.975827	0.05064
4	0	0.317694			
5	1896.625	85.91346	4.536115	9.57E-05	0.438861
5	0	0.000262			
6	47894	2865.308	8.927825	0	0.606188
6	0	9.18E-07			
7	70873.25	3262.519	11.62384	0	0.667123
7	0	4.47E-08			
8	92700.75	5622.712	11.74538	0	0.669429
8	0	3.92E-08			
9	167402.6	9034.683	13.31932	0	0.696642
9	0	7.65E-09			
10	34.125	-2.43269	0.176767	0.99731	0.029576
10	0	0.449349			
11	3.875	-0.95192	0.34339	0.964974	0.055896
11	6.74E-07	0.292675			
12	6.75	1.019231	0.15239	0.998565	0.025602
12	1.1E-07	0.482298			
13	23.5	-2.5	0.332452	0.968735	0.054212
13	0	0.300429			
14	229.375	-4.99038	0.080602	0.999917	0.013706
14	0	0.609049			
15	5348.25	159.8269	0.207685	0.994779	0.03457
15	0	0.412394			
16	8423	277.2308	0.249374	0.989228	0.041223
16	0	0.369322			
17	10357.88	299.5096	0.220146	0.993402	0.036568
17	0	0.398785			
18	19067.75	573.4808	0.227511	0.992479	0.037745
18	0	0.39105			
19	40.5	-2.03846	0.181606	0.99699	0.030361
19	0	0.443237			
20	4.5	0.576923	0.138712	0.999044	0.023357
20	5.58E-09	0.502572			
21	11.375	-4.375	3.821315	0.000531	0.397172
21	0	0.000737			
22	24.625	1.759615	0.207333	0.994815	0.034513
22	0	0.412788			
23	241.625	16.91346	0.954885	0.491681	0.141362
23	0	0.081507			
24	6286	484.6923	2.636558	0.010166	0.312516
24	0	0.004515			
25	9576.75	634.25	1.788839	0.083074	0.23572
25	0	0.018205			
26	12327.38	959.2404	3.055247	0.003545	0.345021
26	0	0.002343			
27	22215.63	1606.529	2.447466	0.016359	0.296754
27	0	0.006111			
28	30.375	0.471154	0.007072	1	0.001218
28	0	0.879478			
29	25.75	0.173077	0.001462	1	0.000252
29	0	0.945027			
30	4.625	0.298077	0.019218	1	0.003303
30	5.32E-06	0.802652			
31	67.25	-1.63462	0.047418	0.999993	0.008109
31	0	0.694741			
32	2168.875	62.74038	0.207733	0.994774	0.034578
32	0	0.412341			
33	2840.875	52.89423	0.103737	0.999736	0.017571
33	0	0.561886			
34	4170.75	92.71154	0.163373	0.99807	0.027396
34	0	0.466986			
35	7122.5	146.4231	0.13617	0.999118	0.022939
35	0	0.5065			
36	215808.5	11361.12	6.743675	7E-07	0.537616
36	0	1.34E-05			
37	10881.13	438.5673	5.228013	1.94E-05	0.474067
37	0	9.98E-05			
38	4.75	0.634615	0.190966	0.996294	0.031876
38	1.73E-10	0.431773			
39	0	0	0	1	0
39	0	0			
40	21.25	2.596154	0.642775	0.771129	0.099767
40	0	0.151423			
41	0.059375	0.001779	0.045592	0.999994	0.007799
41	0	0.700379			
42	59.5	4.5	0.54158	0.853368	0.085402
42	0	0.187384			
43	0.054375	0.00324	0.31985	0.972748	0.052264
43	0	0.3097			
44	74.75	0.865385	0.021286	1	0.003657
44	0	0.792542			
45	0.0685	-0.00065	0.021862	1	0.003755
45	0	0.789818			
46	19.125	2.798077	0.839758	0.592875	0.126474
46	0	0.101873			
47	0.049375	0.003548	0.209648	0.994577	0.034885
47	0	0.410206			
48	31.625	-4.54808	1.059476	0.407666	0.154454
48	0	0.066849			
49	0.08125	-0.01617	2.617468	0.010667	0.310957
49	0	0.004654			
50	2.75	4.019231	4.557717	0.000091	0.440031
50	0.001289	0.000254			
51	0.00975	0.013789	4.263987	0.000182	0.423688
51	0.001294	0.000387			
52	105	-2.38462	0.082782	0.999906	0.014072
52	0	0.604257			
53	0.295875	-0.00149	0.006782	1	0.001168
53	0	0.881961			
54	780.375	9.855769	0.030614	0.999999	0.005251
54	0	0.752469			
55	7.125	1.644231	0.494041	0.887086	0.078493
55	1.47E-09	0.207711			
56	0.002625	0.000452	0.458381	0.909745	0.073243
56	0	0.224717			
57	0.375	0.394231	0.722047	0.700471	0.110709
57	0.067367	0.128768			
58	0.0065	0.007885	0.925664	0.516647	0.137632
58	0.072525	0.08621			
59	0.25	-0.09615	0.08319	0.999904	0.01414
59	0.089123	0.603369			
60	0.016625	-0.01078	0.339786	0.966242	0.055342
60	0.042484	0.2952			
61	0.125	0.182692	0.263073	0.986772	0.043389
61	0.422357	0.35657			
62	0.00625	0.007365	0.199503	0.995566	0.033253
62	0.386384	0.421709			
63	0.125	-0.125	0.512925	0.874144	0.08125
63	0.104136	0.199336			
64	0.009625	-0.00963	0.512925	0.874144	0.08125
64	0.104136	0.199336			
65	0	0	0	1	0
65	0	0			
66	0	0	0	1	0
66	0	0			
67	23.875	-0.49038	0.010219	1	0.001759
67	0	0.85538			
68	0.43575	0.007865	0.014887	1	0.00256
68	0	0.825895			
69	9.625	0.067308	0.000745	1	0.000128
69	0	0.960743			
70	0.836625	0.058606	0.519388	0.869574	0.08219
70	0	0.196562			
71	1015	48.53846	1.127481	0.358174	0.162755
71	0	0.05888			
72	51.75	-3.51923	0.35369	0.961196	0.057476
72	0	0.285609			
73	0.00775	-0.00113	2.536726	0.013071	0.304283
73	0	0.005295			
74	1	1	2.393651	0.018724	0.292135
74	0.00069	0.006666			
75	0.016875	0.018279	2.876337	0.005558	0.331515
75	0.000601	0.003094			
76	4	0.230769	0.011739	1	0.00202
76	5.28E-05	0.845126			
77	0.0525	0.007269	0.078838	0.999925	0.013411
77	1.52E-05	0.612985			
78	4.625	0.375	0.063237	0.999973	0.010785
78	8.7E-10	0.650477			
79	0.066	0.004769	0.05342	0.999988	0.009126
79	3.45E-10	0.677048			
80	7.5	1.423077	0.380387	0.95032	0.061548
80	2.11E-10	0.268287			
81	0.11175	0.018173	0.421233	0.930673	0.067709
81	0	0.244276			
82	2.625	0.221154	0.039498	0.999997	0.006764
82	8.54E-07	0.720195			

ESS-BS induction Deletions/Total Deletions	83	0.064059	-0.0236838	-0.023059	2.06531	0.042411	0.262585	83	0.040375	0.000625	0.001285	1	0.000221
	83	0	0.0080736	0.002883				83	1.26E-06	0.948469			
Splicing Change Deletions:	84	48.47059	-33.595588	-31.93213	30.64455	0	0.840854	84	14.875	1.663462	0.192867	0.99614	0.032183
	84	0	0	0				84	0	0.4295			
Splicing Change Deletions/Total Deletions	85	0.387529	-0.0630294	-0.042683	1.156605	0.338306	0.16626	85	0.3245	0.020346	0.211843	0.994344	0.035238
	85	0	0.0186533	0.061731				85	0	0.407778			
Protein motif disrupting Deletions:	86	20.88235	-10.257353	-12.34389	13.08769	0	0.692922	86	10.625	-2.08654	0.502009	0.881702	0.079659
	86	0	1.061E-07	6E-12				86	7E-12	0.204127			
Protein motif disrupting Deletions/Total Deletions	87	0.691412	-0.0066618	0.049358	0.178291	0.997212	0.029823	87	0.68475	0.056019	0.127614	0.999336	0.021529
	87	0	0.9155315	0.361951				87	0	0.520123			
Conserved Element Rearrangements:	88	826.8235	-519.32353	-510.362	110.9032	0	0.950301	88	307.5	8.961539	0.163657	0.998055	0.027442
	88	0	0	0				88	0	0.4666			
TFBS Rearrangements:	89	31.29412	-18.669118	-17.83258	25.07922	0	0.812171	89	12.625	0.836539	0.236186	0.991283	0.039128
	89	0	0	0				89	0	0.382215			
TFBS Rearrangements/Total Rearrangements	90	0.004118	0.0008824	0.000652	1.301385	0.251513	0.183258	90	0.005	-0.00023	0.093801	0.999833	0.015915
	90	0	0.0155978	0.036638				90	0	0.581201			
miRNA-BS disrupting Rearrangements:	91	0.941177	-0.8161765	-0.402715	1.045849	0.418091	0.152771	91	0.125	0.413462	0.804215	0.625252	0.121773
	91	4.31E-06	0.0166978	0.163305				91	0.534956	0.109262			
miRNA-BS disrupting Rearrangements/Total Rearrangements	92	0.045353	-0.0349779	0.020801	0.682899	0.735798	0.105339	92	0.010375	0.055779	0.94318	0.501612	0.139872
	92	0.003201	0.1866878	0.359197				92	0.679072	0.083355			
ESE-BS deletion Rearrangements:	93	9.058824	-5.0588235	-5.366516	8.277924	0	0.588007	93	4	-0.30769	0.053564	0.999988	0.009151
	93	0	2.223E-06	1.82E-08				93	2.1E-09	0.676636			
ESE-BS deletion Rearrangements/Total Rearrangements	94	0.089235	0.0038897	-0.003389	0.055914	0.999985	0.009548	94	0.093125	-0.00728	0.086692	0.999884	0.014727
	94	0	0.7475943	0.744203				94	2E-12	0.595861			
ESE-BS induction Rearrangements:	95	8.647059	-3.7720588	-3.877828	4.07363	0.000288	0.412577	95	4.875	-0.10577	0.00324	1	0.000558
	95	0	0.0004264	3.5E-05				95	7.71E-08	0.918254			
ESE-BS induction Rearrangements/Total Rearrangements	96	0.085471	0.0272794	0.023914	0.886529	0.550919	0.132584	96	0.11275	-0.00337	0.010596	1	0.001824
	96	0	0.0621828	0.057232				96	3.2E-11	0.852766			
ESS-BS deletion Rearrangements:	97	6.235294	-4.7352941	-3.850679	12.51718	0	0.683357	97	1.5	0.884615	0.830141	0.601603	0.125207
	97	0	3E-10	1.55E-09				97	0.000701	0.103815			
ESS-BS deletion Rearrangements/Total Rearrangements	98	0.113412	-0.0489118	-0.015258	1.519193	0.155902	0.207563	98	0.0645	0.033654	1.031726	0.429068	0.15102
	98	0	0.0034967	0.275674				98	3E-05	0.070433			
ESS-BS induction Rearrangements:	99	5.235294	-3.6102941	-3.38914	5.461416	1.15E-05	0.484967	99	1.625	0.221154	0.037396	0.999998	0.006406
	99	0	2.439E-05	5.19E-06				99	0.001707	0.727433			
ESS-BS induction Rearrangements/Total Rearrangements	100	0.094471	-0.0227206	-0.014163	0.161456	0.998164	0.027083	100	0.07175	0.008558	0.0239	1	0.004104
	100	3.72E-09	0.3595342	0.505649				100	0.003994	0.780487			
Splicing Change Rearrangements:	101	7.470588	-2.5955882	-2.932127	2.032806	0.045944	0.259525	101	4.875	-0.33654	0.023902	1	0.004104
	101	0	0.016571	0.001914				101	2.46E-06	0.780477			
Splicing Change Rearrangements/Total Rearrangements	102	0.276118	0.0573824	0.063498	0.376953	0.951806	0.061026	102	0.3335	0.006115	0.002777	1	0.000479
	102	0	0.2867535	0.171327				102	7.5E-09	0.924306			
Protein motif disrupting Rearrangements:	103	19.47059	-13.095588	-9.932127	14.2827	0	0.711194	103	6.375	3.163462	2.192529	0.030949	0.274322
	103	0	1.4E-11	7.2E-10				103	2.65E-09	0.009257			
Protein motif disrupting Rearrangements/Total Rearrangements	104	0.711647	-0.0026471	0.042199	0.200665	0.99546	0.033441	104	0.709	0.044846	0.157949	0.998328	0.026511
	104	0	0.9571728	0.320921				104	0	0.474447			
Nonsense SNPs:	105	36.05882	-19.558824	-17.59729	13.78554	0	0.703863	105	16.5	1.961539	0.309376	0.975827	0.05064
	105	0	3.18E-10	7.6E-11				105	0	0.317694			
Nonsense SNPs/Total SNPs	106	0.006765	0.0036103	0.00462	5.530968	9.9E-06	0.488129	106	0.010375	0.00101	0.188954	0.996452	0.031551
	106	0	0.0004481	6.45E-07				106	0	0.434198			
Frameshift Structural Variants:	107	108.0588	-55.308824	-55.75113	38.67192	0	0.869581	107	52.75	-0.44231	0.003529	1	0.000608
	107	0	0	0				107	0	0.914696			
Frameshift Structural Variants/Total Variants	108	0.493588	0.0110368	0.023027	0.423946	0.929242	0.068115	108	0.504625	0.01199	0.123202	0.999432	0.0208
	108	0	0.5126746	0.114744				108	0	0.527403			
Frameshift Insertions:	109	38.52941	-15.029412	-17.52941	15.71517	0	0.730423	109	23.5	-2.5	0.332452	0.968735	0.054212
	109	0	5.672E-09	0				109	0	0.300429			
Frameshift Insertions/Total Insertions	110	0.646299	0.0534038	0.011878	0.395274	0.943578	0.063803	110	0.699703	-0.04153	0.439505	0.920733	0.070439
	110	0	0.1299845	0.692792				110	0	0.234405			
Frameshift Deletions:	111	61.76471	-37.139706	-35.38009	37.2791	0	0.865364	111	24.625	1.759615	0.207333	0.994815	0.034513
	111	0	0	0				111	0	0.412788			
Frameshift Deletions/Total Deletions	112	0.666865	-0.0596913	0.018231	1.380159	0.212393	0.192219	112	0.607173	0.077922	1.851213	0.071516	0.24195
	112	0	0.0263698	0.422459				112	0	0.016371			
Frameshift Rearrangements:	113	7.764706	-3.1397059	-2.841629	2.045756	0.044504	0.260747	113	4.625	0.298077	0.019218	1	0.003303
	113	0	0.0060861	0.00397				113	5.32E-06	0.802652			
Frameshift Rearrangements/Total Rearrangements	114	0.11763	0.0334828	0.037024	0.602097	0.805599	0.094047	114	0.151112	0.003541	0.003863	1	0.000666
	114	4E-12	0.1790721	0.085187				114	5.9E-08	0.91077			
Splicing Change Variants:	115	138.4118	-92.036765	-87.181	57.676	0	0.908627	115	46.375	4.855769	0.355299	0.960584	0.057722
	115	0	0	0				115	0	0.284525			
Splicing Change Variants/Total Variants	116	0.100824	0.0178015	0.026253	3.212646	0.002391	0.35646	116	0.118625	0.008452	0.240245	0.990682	0.039774
	116	0	0.0147919	5.79E-05				116	0	0.378179			
Probably Damaging nscSNPs:	117	784.8235	-466.44853	-457.6697	92.54759	0	0.941026	117	318.375	8.778846	0.257839	0.98775	0.042563
	117	0	0	0				117	0	0.361369			
Probably Damaging nscSNPs/Total nscSNPs	118	0.280294	0.0534559	0.057475	19.91319	0	0.774435	118	0.33375	0.004019	0.072076	0.99995	0.012274
	118	0	2.8E-11	0				118	0	0.628615			
Possibly Damaging nscSNPs:	119	484.7059	-309.70588	-315.552	131.5577	0	0.957775	119	175	-5.84615	0.509795	0.876332	0.080794
	119	0	0	0				119	0	0.200695			
Possibly Damaging nscSNPs/Total nscSNPs	120	0.173294	0.0102059	0.001398	1.66458	0.111487	0.222997	120	0.1835	-0.00881	1.883937	0.066078	0.245179
	120	0	0.0029218	0.623996				120	0	0.015488			
Protein motif damaging Variants:	121	307	-175.375	-176.6154	123.5228	0	0.955151	121	131.625	-1.24038	0.012806	1	0.002203
	121	0	0	0				121	0	0.838334			
Protein motif damaging Variants/Total Variants	122	0.303471	0.0330294	0.036068	3.917936	0.00042	0.403165	122	0.3365	0.003039	0.022234	1	0.003819
	122	0	0.000849	3.45E-05				122	0	0.788083			
TFBS Disrupting Variants:	123	202.7059	-120.83088	-122.4751	52.51537	0	0.900541	123	81.875	-1.64423	0.048681	0.999992	0.008323
	123	0	0	0				123	0	0.690914			
TFBS Disrupting Variants/Total Variants	124	0.001	0.001	0.000846	26.75909	0	0.821862	124	0.002	-0.00015	0.401732	0.940503	0.064777
	124	0	0	0				124	0	0.255388			
miRNA-BS Disrupting Variants:	125	81.17647	-58.426471	-54.02262	55.21824	0	0.904947	125	22.75	4.403846	1.671904	0.10959	0.223759
	125	0	0	0				125	0	0.022256			
miRNA-BS Disrupting Variants/Total Variants	126	0.051941	0.0016838	0.003751	0.281577	0.982905	0.0463	126	0.053625	0.002067	0.085448	0.999891	0.014519
	126	0	0.6167729	0.196904				126	0	0.598504			
ESE-BS Disrupting Variants:	127	483.8235	-331.69853	-326.0543	142.7852	0	0.960965	127	152.125	5.644231	0.393884	0.944228	0.063592
	127	0	0	0				127	0	0.260038			
ESE-BS Disrupting Variants/Total Variants	128	0.119765	0.0043603	0.006774	1.316912	0.243366	0.18504	128	0.124125	0.002414	0.139646	0.999016	0.023511
	128	0	0.1305763	0.007306				128	0	0.501142			
ESS-BS Disrupting Variants:	129	197.8235	-133.69853	-132.8235	147.3852	0	0.962137	129	64.125	0.875	0.02121	1	0.003644
	129	0	0	0				129	0	0.792905			
ESS-BS Disrupting Variants/Total Variants	130	0.121765	0.0086103	0.00362	0.58213	0.821882	0.091212	130	0.130375	-0.00499	0.24013	0.990699	0.039756
	130	0	0.0673364	0.366207				130	0	0.378293			
**Total Likely Functional Novel Variants:**	131	2337.824	-1471.5735	-1463.977	140.4062	0	0.96033	131	866.25	7.596154	0.053636	0.999988	0.009163
	131	0	0	0				131	0	0.676433			
**Total Genotypes (not just novel)**	132	6010608	-403707.82	-324872.4	34.8663	0	0.857376	132	5606900	78835.38	1.088963	0.385681	0.158074
	132	0	0	0				132	0	0.063258			
synonymous to nonsynonymous rate	133	0.903288	-0.2505418	-0.243618	92.94851	0	0.941265	133	0.652746	0.006924	0.065099	0.999969	0.011099
	133	0	0	0				133	0	0.645725			
Functional Variant/Total Variants	134	0.000389	-0.0002343	-0.000235	138.5763	0	0.959827	134	0.000155	-8E-07	0.017623	1	0.003029
	134	0	0	0				134	0	0.810852			
Functional Variant - Prob Damaging/Total Variants	135	0.000308	-0.0001849	-0.000184	127.7127	0	0.956558	135	0.000123	7E-07	0.01775	1	0.003051
	135	0	0	0				135	0	0.810185			
Functional Nonsense SNPs/Total Variants	136	0.000006	-0.0000031	-2.8E-06	11.7318	0	0.669173	136	2.9E-06	3E-07	0.241615	0.990473	0.039992
	136	0	3.399E-09	8.94E-10				136	0	0.376831			
Functional Frameshift Structural Variants/Total Variants	137	0.000018	-0.0000086	-8.8E-06	34.66552	0	0.856668	137	9.4E-06	-2E-07	0.033158	0.999999	0.005685
	137	0	0	0				137	0	0.742746			
Functional Insertions/Total Variants	138	6.4E-06	-0.0000022	-2.7E-06	13.16921	0	0.694241	138	4.2E-06	-5E-07	0.477474	0.897912	0.076061
	138	0	1.441E-07	4E-12				138	0	0.21541			
Functional Deletions/Total Variants	139	1.03E-05	-0.0000059	-5.6E-06	33.44367	0	0.852206	139	4.4E-06	2E-07	0.132643	0.999214	0.022358
	139	0	0	0				139	0	0.512039			
Functional Rearrangements/Total Variants	140	1.3E-06	-0.0000005	-4E-07	1.62337	0.122735	0.218684	140	8E-07	0	0.009052	1	0.001558
	140	0	0.0151346	0.009147				140	2.36E-06	0.863801			
Functional Splicing Change Variants/Total Variants	141	0.000023	-0.0000148	-0.000014	53.56133	0	0.902293	141	8.3E-06	7E-07	0.268149	0.985776	0.04419
	141	0	0	0				141	0	0.352002			
Probably Damaging nscSNPs/Total Variants	142	0.000131	-0.0000738	-0.000073	86.37494	0	0.937076	142	5.68E-05	8E-07	0.058846	0.999981	0.010044
	142	0	0	0				142	0	0.662032			
Possibly Damaging nscSNPs/Total Variants	143	8.06E-05	-0.0000494	-5.09E-05	127.4988	0	0.956489	143	3.12E-05	-1.5E-06	1.047792	0.416595	0.153012
	143	0	0	0				143	0	0.068333			
Functional Protein motif damaging Variants/Total Variants	144	5.11E-05	-0.0000276	-2.81E-05	118.6959	0	0.953412	144	2.35E-05	-6E-07	0.089144	0.999868	0.015137
	144	0	0	0				144	0	0.59072			
Functional TFBS Disrupting Variants/Total Variants	145	3.37E-05	-0.0000191	-1.96E-05	50.69206	0	0.897331	145	1.46E-05	-5E-07	0.129955	0.999281	0.021915
	145	0	0	0				145	0	0.516331			
Functional miRNA-BS Disrupting Variants/Total Variants	146	1.35E-05	-0.0000094	-8.7E-06	52.36863	0	0.90029	146	4.1E-06	7E-07	1.475238	0.172145	0.202775
	146	0	0	0				146	0	0.031384			
Functional ESE-BS Disrupting Variants/Total Variants	147	8.05E-05	-0.0000533	-5.27E-05	141.6046	0	0.960653	147	2.71E-05	6E-07	0.138443	0.999052	0.023313
	147	0	0	0				147	0	0.502985			
Functional ESS-BS Disrupting Variants/Total Variants	148	3.29E-05	-0.0000215	-2.15E-05	143.0919	0	0.961046	148	1.14E-05	0	3.7E-06	1	6E-07
	148	0	0	0				148	0	0.997225			
Functional Variant/Total Pop Spec Variants	149	0.002611	0.0014026	0.001236	166.3178	0	0.966302	149	0.004014	-0.00017	1.586113	0.133784	0.214743
	149	0	0	0				149	0	0.025831			
Functional Variant - Prob Damaging/Total Pop Spec Variants	150	0.00207	0.0011335	0.001033	138.3195	0	0.959756	150	0.003203	-0.0001			
	150	0	0	0				150	0	0.133128	0.705606	0.715384	0.108461
Functional Nonsense SNPs/Total Pop Spec Variants	151	0.00207	0.0011335	0.001033	138.3195	0	0.959756	151	0.003203	-0.0001			
	151	0	0	0				151	0	0.133128	0.705606	0.715384	0.108461
Functional Frameshift Structural Variants/Total Pop Spec Variants	152	0.000121	0.0001235	0.000109	23.62756	0	0.802906	152	0.000245	-1.5E-05			
	152	0	0	0				152	0	0.402116	0.217039	0.993766	0.036071
Functional Insertions/Total Pop Spec Variants	153	4.33E-05	0.0000656	4.88E-05	17.47585	0	0.750815	153	0.000109	-1.7E-05			
	153	0	0	4.6E-11				153	0	0.095597	0.872286	0.563596	0.130733
Functional Deletions/Total Pop Spec Variants	154	6.89E-05	0.0000453	4.74E-05	11.97359	0	0.673673	154	0.000114	2.1E-06			
	154	0	2.854E-08	1.01E-10				154	0	0.82878	0.01439	1	0.002475
Functional Rearrangements/Total Pop Spec Variants	155	8.8E-06	0.0000126	1.27E-05	3.212855	0.002389	0.356475	155	2.14E-05	1E-07			
	155	0.000101	0.0014137	0.000215				155	1.8E-06	0.981147	0.000172	1	2.96E-05
Functional Splicing Change Variants/Total Pop Spec Variants	156	0.000154	0.0000605	7.04E-05	6.792135	7E-07	0.539395	156	0.000215	9.9E-06			
	156	0	4.003E-05	1.06E-07				156	0	0.600675	0.084436	0.999897	0.014349
Probably Damaging nscSNPs/Total Pop Spec Variants	157	0.000876	0.0005993	0.000565	86.94694	0	0.937464	157	0.001475	-3.4E-05			
	157	0	0	0				157	0	0.447675	0.178082	0.997226	0.029789
Possibly Damaging nscSNPs/Total Pop Spec Variants	158	0.000542	0.0002691	0.000203	67.66226	0	0.921048	158	0.000811	-6.6E-05			
	158	0	0	0				158	0	0.000371	4.292221	0.000171	0.4253
Functional Protein motif damaging Variants/Total Pop Spec Variants	159	0.000344	0.0002656	0.00023	43.34244	0	0.881976	159	0.00061	-3.6E-05			
	159	0	0	0				159	0	0.172127	0.581473	0.822409	0.091119
Functional TFBS Disrupting Variants/Total Pop Spec Variants	160	0.000226	0.0001534	0.000127	30.01095	0	0.838038	160	0.000379	-2.6E-05			
	160	0	0	0				160	0	0.138271	0.686988	0.73214	0.105903
Functional miRNA-BS Disrupting Variants/Total Pop Spec Variants	161	9.05E-05	0.000015	2.91E-05	4.094529	0.000274	0.413818	161	0.000106	1.41E-05			
	161	0	0.0318273	5.15E-06				161	0	0.105859	0.820235	0.610621	0.123898
Functional ESE-BS Disrupting Variants/Total Pop Spec Variants	162	0.000541	0.0001645	0.000154	26.45465	0	0.820181	162	0.000705	-1.1E-05			
	162	0	0	0				162	0	0.62313	0.074406	0.999942	0.012666
Functional ESS-BS Disrupting Variants/Total Pop Spec Variants	163	0.000221	0.0000757	6.52E-05	10.42582	0	0.642545	163	0.000297	-1.1E-05			
	163	0	9.075E-09	8.58E-09				163	0	0.498734	0.141229	0.998967	0.023771
