# Supplementary material for: Clinical Implications of Human Population Differences in Genome-Wide Rates of Functional Genotypes
Source: Front Genet. 2012 Nov 1;3:211. doi: 10.3389/fgene.2012.00211 (PMC3485509; doi:10.3389/fgene.2012.00211)
Supplement: Supplementary Data Sheet S7 — False positive variants associated with the identification of a homozygous CF mutation if that homozygous CF mutation had a pathogenicity score greater than the average Polyphen2, SIFT or average Polyphen2/SIFT scores for 567 known CF mutations. [file 32001_Schork_DataSheet7.PDF]

PolyPhen Score > 0.769 (N=567)

Indiv	Source	CGI EUR 8	CGI ASN 8	CGI AFR 8
EUROPEAN	STSI	78/361	99/361	140/361
AFRICAN AMERICAN	CGI	111/284	100/284	66/284
MEXICAN	CGI	92/363	91/363	162/363
EAST INDIAN	CGI	79/326	68/326	136/326
PUERTA RICAN	CGI	61/319	80/319	125/319

SIFT Score > 0.891 (N=567)

Indiv	Source	CGI EUR 8	CGI ASN 8	CGI AFR 8
EUROPEAN	STSI	168/784	191/784	256/784
AFRICAN AMERICAN	CGI	228/680	216/680	114/680
MEXICAN	CGI	183/752	194/752	267/752
EAST INDIAN	CGI	181/773	158/773	243/773
PUERTA RICAN	CGI	135/712	133/712	215/712

Average Score > 0.83 (N=567)

Indiv	Source	CGI EUR 8	CGI ASN 8	CGI AFR 8
EUROPEAN	STSI	69/306	82/306	121/306
AFRICAN AMERICAN	CGI	100/250	90/250	55/250
MEXICAN	CGI	86/312	82/312	146/312
EAST INDIAN	CGI	67/273	54/273	119/273
PUERTA RICAN	CGI	53/270	66/270	104/270
